# Labeling AI-generated media online

**DOI:** 10.1093/pnasnexus/pgaf170

**Published:** 2025-05-28

**Authors:** Chloe Wittenberg, Ziv Epstein, Gabrielle Péloquin-Skulski, Adam J Berinsky, David G Rand

**Affiliations:** Sloan School of Management, Massachusetts Institute of Technology, Cambridge, MA 02142, USA; Stanford Institute for Human-Centered AI, Stanford University, Palo Alto, CA 94305, USA; Media Lab, Massachusetts Institute of Technology, Cambridge, MA 02139, USA; Department of Political Science, Massachusetts Institute of Technology, Cambridge, MA 02139, USA; Department of Political Science, Massachusetts Institute of Technology, Cambridge, MA 02139, USA; Sloan School of Management, Massachusetts Institute of Technology, Cambridge, MA 02142, USA; Department of Brain and Cognitive Sciences, Massachusetts Institute of Technology, Cambridge, MA 02139, USA

**Keywords:** generative artificial intelligence, misinformation, labeling, social media, survey experiments

## Abstract

Recent advancements in generative AI have raised widespread concern about the use of this technology to spread audio and visual misinformation. In response, there has been a major push among policymakers and technology companies to label AI-generated media appearing online. It remains unclear, however, what *types* of labels are most effective for this purpose. Here, we evaluate two (potentially complementary) strategies for labeling AI-generated content online: (i) a *process-based* approach, aimed at clarifying how content was made and (ii) a *harm-based* approach, aimed at highlighting content's potential to mislead. Using two preregistered survey experiments focused on misleading, AI-generated images (total *n* = 7,579 Americans), we assess the consequences of these different labeling strategies for viewers' beliefs and behavioral intentions. Overall, we find that all of the labels we tested significantly decreased participants' belief in the presented claims. However, in both studies, labels that simply informed participants that content was generated using AI tended to have little impact on respondents' stated likelihood of engaging with their assigned post. Together, these results shed light on the relative advantages and disadvantages of different approaches to labeling AI-generated media online.

**Significance Statement**

Concerns about the ability of generative AI to amplify the spread of misinformation have catalyzed substantial investments in systems for labeling AI-generated media online. Nevertheless, it remains unclear how such labels should be designed and applied. Using two online survey experiments, focused on misleading, AI-generated images, we distinguish *process-based* labels (explaining how content was made) from *harm-based* labels (highlighting content's potential to mislead). Across studies, labeling of any kind decreases the perceived credibility of presented content. However, identifying content as “AI-generated” has little impact on individuals' stated likelihood of engaging with it (across a range of self-reported behaviors). These results suggest the inferences people draw from labels applied to AI-generated media may depend on what language is used.

## Introduction

The past several years have been marked by a precipitous rise in both the availability and sophistication of generative AI. Although proponents of generative AI emphasize the value of this technology across a range of settings (1, 2), many policymakers and members of the public have expressed anxiety about its growing influence (3, 4). Much of this concern is rooted in fears that generative AI will accelerate the spread of false and misleading content (5–7); as generative AI tools become increasingly advanced, people can more easily—and rapidly—fabricate high-quality images, audio, and video of events that never took place. Both experts and commentators alike have speculated that this surge of generative AI could create a “wave of AI-powered disinformation” (8) and further “blur lines between fiction and reality” (9), a danger that some reports have characterized as the “biggest short-term threat to the global economy” (10).

One commonly proposed response to this challenge involves visibly labeling AI-generated media (e.g. by displaying textual or graphical markers alongside the relevant content). Companies such as Meta (11), Google (12, 13), and TikTok (14, 15) have all recently unveiled strategies for appending labels to AI-generated content appearing on their platforms or created using their products. Moreover, efforts to regulate generative AI frequently contain provisions related to labeling (16–20). For example, the California AI Transparency Act, passed in September 2024, includes provisions around “clear, conspicuous” disclosures for AI-generated images, videos, and audio (21), and the EU Artificial Intelligence Act, which came into force in August 2024, likewise mandates transparent labeling of certain types of AI-generated media (22).

This mounting interest in and attention to AI labeling comports with existing work suggesting that content warnings can decrease individuals' vulnerability to deception. Theoretically, labeling has the potential to address crucial gaps in lay understanding and knowledge, while still giving people agency over the content they consume (23). Though the public tends to be broadly familiar with the concept of AI (3, 24), many people report limited exposure to and direct experience with generative AI technologies (25, 26) and often struggle to discern whether images, audio, and video have been created using AI (e.g. (27–30); for studies of AI-generated text, see also (31, 32)). Labels may therefore provide valuable information that individuals could not otherwise intuit on their own. Empirically, labeling has also shown previous success in related settings; labeling content that fact-checkers have flagged as false generally reduces viewers' belief in (33–38) and likelihood of sharing (36, 39–41) this content, even in cases where people are predisposed to discount labels as biased ((42); for a review, see Ref. (43)). But these established strategies may or may not work for AI-generated content (44, 45)—particularly audio and visual media (46, 47), which often seem especially credible and realistic (48, 49) and tend to attract outsized engagement (28).

How, then, should AI-generated content be labeled? There are two distinct objectives that AI labeling might seek to address (50). First, labels could spotlight the *process* by which media were made, with the goal of informing users that content may have been created or modified using AI. In line with this approach, AI labeling proposals—among both policymakers (51) and technology companies (15, 52)—often apply to *all* forms of AI-generated media, regardless of their subject matter or potential to mislead. Alternatively, because much of the impetus to label AI-generated content arises from concerns about its ability to deceive (28, 53), labeling programs could instead focus on the smaller subset of misleading, AI-generated content that poses an especially high risk of *harm* (54).

Critically, while these process- and harm-based labeling strategies are not mutually exclusive, they may give rise to notably different designs (e.g. language, graphics), implementation (e.g. methods for detecting relevant content), and evaluation (e.g. metrics used to define and benchmark success). On the one hand, because process-based labeling programs tend to implicate a broad array of content—both misleading and not—such efforts might seek to inform viewers about how content was made without influencing downstream behaviors (e.g. sharing or commenting). On the other hand, more targeted efforts to mitigate the persuasiveness and reach of AI-generated misinformation might aim to reduce both belief in and likelihood of engaging with this content, given the potential damages such content could inflict.

Despite these conceptual distinctions, however, process- and harm-based signals may not always be interpreted as expected. Efforts to flag false or misleading content can help viewers better appraise content's (in)accuracy (43, 55) but may be less informative about why this content is suspect (e.g. because it was digitally altered, see Ref. (56)). Conversely, people may use provenance indicators to intuit a message's credibility, even in cases where such cues convey only limited information about the content's veracity (57, 58). Indeed, news articles identified as AI-generated tend to be perceived as less trustworthy than articles attributed to human authors (59), even when the stories are in fact true (60, 61). Most notably, recent work (62) finds that labeling news headlines as AI-generated reduces their perceived accuracy and likelihood of being shared—irrespective of whether the underlying claims are true or false—because people assume the story was entirely fabricated using AI.

Nevertheless, this prior research on AI disclosures largely examines the domain of *text*, despite the fact that many extant labeling proposals have prioritized *visual* media (11, 63). Although both modalities merit investigation, they vary in both their usage and means of deception (64). Whereas concerns about AI-generated text tend to be rooted in fears that this text may seem less suspicious than traditional “fake news” (because of its language, tone, or style; see Ref. (65)), concerns about AI-generated images instead emphasize their ability to provide seemingly probative evidence of events that never took place (66). AI labels may therefore operate in fundamentally different ways across visual versus textual media—leaving open key questions about whether, and under what conditions, different types of labeling approaches shape individuals' appraisals of and responses to AI-generated images.

Here, we take up these questions. Using two preregistered survey experiments, we evaluate the relative impact of different types of AI labels, applied to real-world cases of visual misinformation (total *n* = 7,579 Americans). In both studies, respondents were shown a social media post containing an image that journalists or fact-checkers had previously flagged as both AI-generated and misleading. Some respondents viewed an unlabeled version of the post, whereas others viewed a version of the post containing one of several warning labels, designed to convey process- and/or harm-based cues. To improve generalizability, we examined a diverse set of posts (14 in the first experiment, 29 in the second) varying in their topic (e.g. political vs. nonpolitical), subject (e.g. religious leaders, celebrities, politicians), and level of societal importance (for additional information, see SI Appendix, Table S2).

In the first study, conducted in October 2023 (Experiment 1, *n* = 3,223), respondents were randomized to one of five conditions. Respondents in the control group were shown an unlabeled version of their assigned post. The remaining respondents were shown a version of the post with one of four labels appended: (i) an “AI-Generated” (AIG) label indicating that the post contained “media that was generated using artificial intelligence,” (ii) an “Artificial” label indicating that the post contained “artificial content that has been edited or digitally altered,” (iii) a “Manipulated” label indicating that the post contained “media that was altered, manipulated, or fabricated,” or (iv) a “False” label indicating that the post contained “media that has been reviewed by independent fact-checkers.” We modeled these four treatments on policy language and real-world labels already in place on social media platforms, in order to enhance external validity and examine designs that companies might realistically adopt. Following Ref. (50), we also aimed to provide a range of cues about the process by which the content was created, as well as the potential for the content to mislead (see also SI Appendix, Fig. S1).

**Fig. 1.**
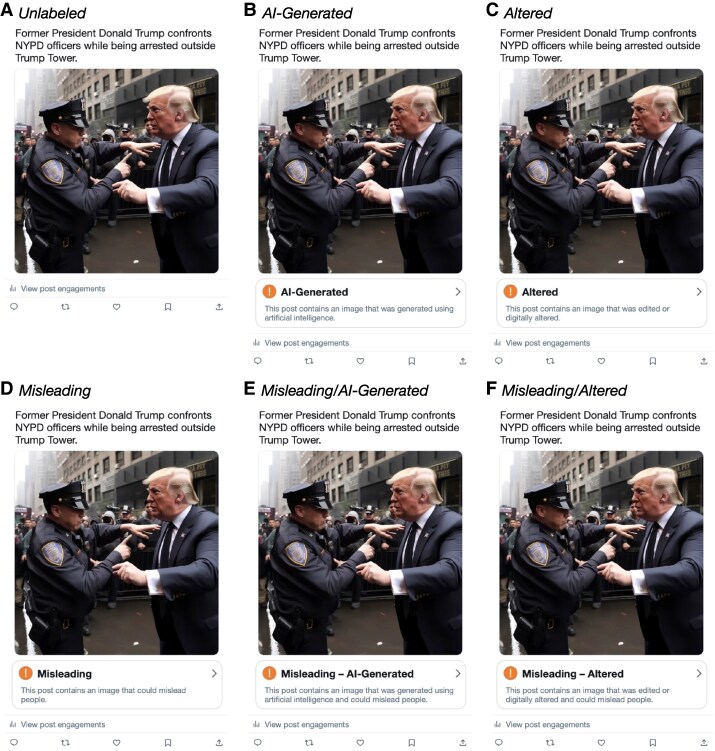
Example stimuli for each of the six conditions in Experiment 2. The labels in Experiment 1 had the same format and placement but used slightly different language (see SI Appendix, Section 1.3). For all stimuli, we used AI-generated images that had been previously posted online; in this case, the image was created using Midjourney, Version 4. Image from X post by @EliotHiggins at https://x.com/EliotHiggins/status/1637927681734987777 [accessed 2025 April 24].

The second experiment, administered in December 2023 (Experiment 2, *n* = 4,356), followed a similar approach as the first, with one major change: rather than adapting our labels from a wide range of existing exemplars, we systematically manipulated the content of the labels using a 3 × 2 factorial design. The first factor indicated whether respondents received a *process* cue, in the form of either a broad statement that the image was “edited or digitally altered” or a specific disclosure that the image was “generated using artificial intelligence,” whereas the second indicated whether respondents viewed a harm-based *veracity* cue conveying that the image “could mislead people.” In addition to facilitating a more direct comparison of process and harm-based strategies, this design allows us to juxtapose these two approaches against “hybrid” labels that combine both sets of signals. Figure 1 provides an example of how these labels appeared in Experiment 2 (see also SI Appendix, Fig. S2 and Table S3).

After viewing their assigned post, respondents first reported their likelihood of engaging with the content (e.g. via sharing or “liking” the post). Then, after completing a distractor task intended to direct their attention away from the post—and, where applicable, the associated label—respondents rated their belief in the post's core claims (see SI Appendix, Table S4) and, in Experiment 2, their perceptions of the credibility of the image. We chose to measure both beliefs and (self-reported) behavior because they are related but conceptually distinct phenomena; for instance, individuals' choice of whether to interact with content online is only partially a function of its perceived accuracy and may reflect other factors (such as its personal relevance or entertainment value; see (67)). Consequently, a change in one's beliefs may not necessarily yield a commensurate shift in engagement, or vice versa, and (different types of) warning labels may exert different influences on these two outcomes. Lastly, respondents assigned to a labeled post evaluated several aspects of the label itself, including the extent to which it provided different types of information (e.g. about how the content was made or whether it was true or false). The exact wording of these items (all measured using five-point scales) can be found in SI Appendix, Section 1.4. Following our preanalysis plan, we analyzed responses to these items using linear regression models with stimulus fixed effects and heteroskedastic-robust standard errors; in all cases, estimated treatment effects and 95% CI are expressed as standard deviations of the outcome scale.

## Results

Overall, we find that labels reduce the believability of the presented content. In both studies, respondents who were shown a labeled post express significantly less belief in the core claims implied by the AI-generated image, compared to respondents in the unlabeled control group (Exp. 1: −0.21 SD, 95% CI: [−0.29, −0.12]; Exp. 2: −0.25 SD [−0.33, −0.17]). In the second experiment, which tracked perceptions of the images themselves, we likewise find that respondents who were shown a warning label rate their assigned image as significantly less credible, relative to respondents in the control group [−0.34 SD (−0.42, −0.26)]. Warning labels also influence individuals' stated likelihood of engaging with AI-generated media, though the effects on engagement intentions tend to be somewhat weaker than those for beliefs. Across conditions, respondents are moderately less likely to say they would share (Exp. 1: −0.15 SD [−0.24, −0.07]; Exp. 2: −0.11 SD [−0.20, −0.03]), “like” or favorite (Exp. 1: −0.17 SD [−0.26, −0.08]; Exp. 2: −0.09 SD [−0.17, −0.001]), and seek out more information about (Exp. 1: −0.11 SD [−0.19, −0.02]; Exp. 2: −0.08 SD [−0.17, −0.003]) labeled vs. unlabeled posts, though the estimated effect of labeling on commenting and replying is not statistically significant in either experiment (Exp. 1: −0.07 SD [−0.16, 0.02]; Exp. 2: −0.03 SD [−0.11, 0.06]; for a full summary of the aggregate results, see SI Appendix, Sections 2.1 and 4.3).

### Effect of labeling on beliefs

**Fig. 2.**
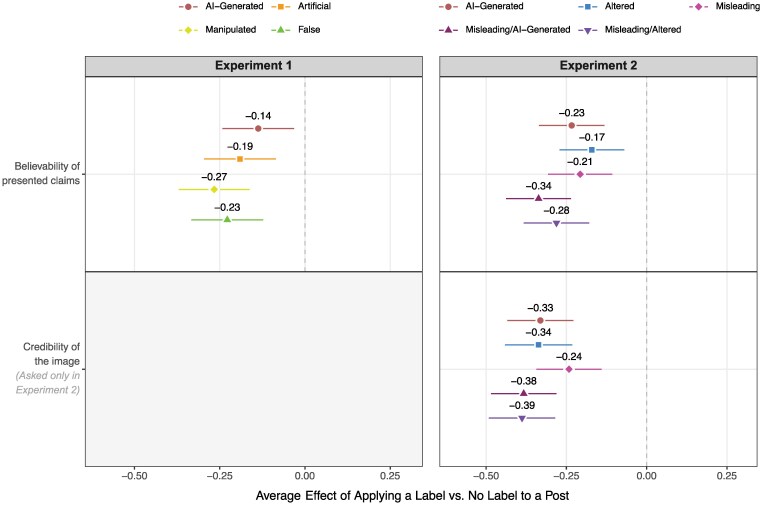
Average treatment effect of assignment to a labeled vs. unlabeled post on *beliefs,* disaggregated by labeling condition. Effects are expressed in units of standard deviation and are estimated using linear regression models with stimulus fixed effects. 95% CI are based on robust standard errors. In all cases, negative values indicate that respondents assigned to a given label were less likely to believe the presented information, compared to respondents in the control group.

How, though, do these effects vary *across* labels? We begin by examining the impact of different labels on participants' beliefs. In Fig. 2, we disaggregate the estimated treatment effects by labeling condition (e.g. “AI-Generated,” “False”). Across the board, labels reduce the believability of the presented content; respondents shown a labeled vs. unlabeled post express significantly more skepticism about the post's core claims and ascribe significantly less credibility to the image, regardless of the exact label to which they were assigned. However, the magnitude of this effect is not entirely uniform across groups. In Experiment 1, exposure to the process-based “AI-Generated” label has a modest, negative effect on individuals' stated belief in the presented claims [−0.14 SD (−0.24, −0.03)]. By comparison, the effects trend somewhat larger for labels containing language typically associated with misleading content (50), including the “Manipulated” label (−0.27 SD [−0.37, −0.16]; compared to AIG: −0.13 SD [−0.24, −0.02]) and “False” label (−0.23 SD [−0.33, −0.12]; compared to AIG: −0.09 SD [−0.20, 0.02]), though the difference between labels is not statistically significant in the latter case (see also SI Appendix, Section 4.3 for a summary of results after adjusting for multiple comparisons).

In Experiment 2, we further probe differences between labels by systematically varying the presence or absence of process- and harm-based cues. Overall, we find that both sets of cues transmit signals about content's reliability (for additional details, see SI Appendix, Tables S7-S8). In regression models interacting indicators of exposure to these two types of treatments, the provision of each cue, on average, reduces respondents' belief in the post's claims (AIG: −0.18 SD [−0.25, −0.11]; Altered: −0.12 SD [−0.19, −0.05]; Misleading: −0.14 SD [−0.20, −0.08]) and the perceived credibility of the image (AIG: −0.23 SD [−0.31, −0.16]; Altered: 0.24 SD [−0.31, −0.17]; Misleading: −0.11 SD [−0.17, −0.05]). In short, exposure to either a process or harm-based cue decreases the believability of the post and its associated image. However, there is no evidence that the inclusion of one type of cue magnifies the (negative) impact of the other. On the contrary, the interaction terms are positively vs. negatively signed for both beliefs (Misleading × AIG: 0.10 SD [−0.04, 0.25]; Misleading × Altered: 0.10 SD [−0.04, 0.24]) and perceived credibility (Misleading × AIG: 0.19 SD [0.05–0.33]; Misleading × Altered: 0.19 SD [0.05–0.34]). In other words, we do not find that the effect of exposure to a process-based cue is larger when the label also includes a harm-based cue (or vice versa); when it comes to “hybrid” labels, the whole does not seem to be greater than the sum of its parts.

Nevertheless, such hybrid labels may still mitigate the persuasiveness of AI-generated misinformation. As shown in the right panel of Fig. 2, treatments that blend process- and harm-based cues tend to have sizable, negative effects on viewers' belief in the presented claims (Misleading/AIG: −0.34 SD [−0.44, −0.24]; Misleading/Altered: −0.28 SD [−0.38, −0.18]) and the perceived credibility of the image (Misleading/AIG: −0.38 SD [−0.48, −0.28]; Misleading/Altered: 0.39 SD [−0.49, −0.28]). Taken together, however, the results of our two studies point to a more general pattern: labeling of *any* kind (including process-based, harm-based, or hybrid labels) reduces the believability of posts containing AI-generated images.

### Effect of labeling on engagement intentions

**Fig. 3.**
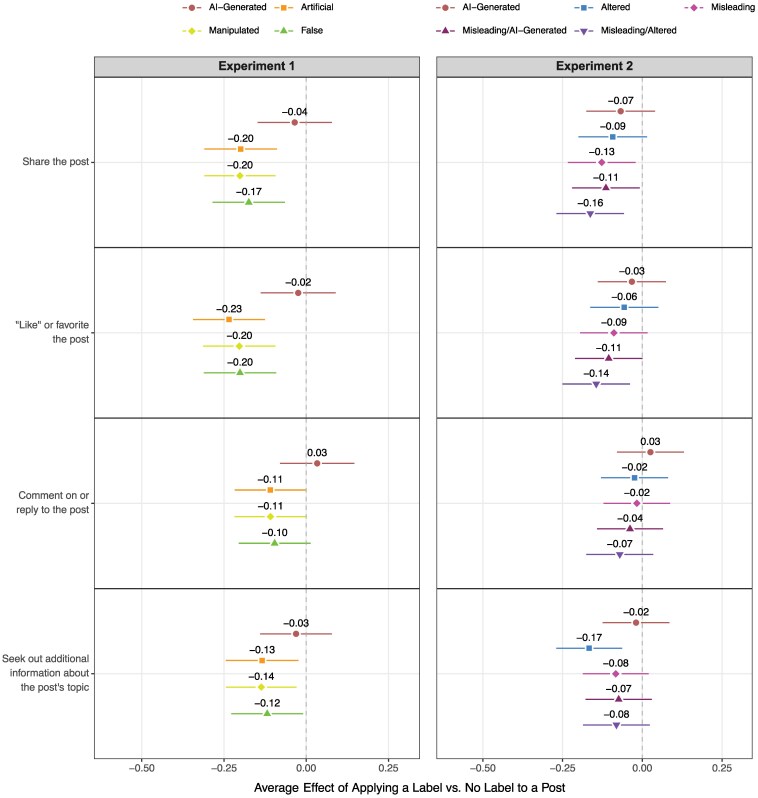
Average treatment effect of assignment to a labeled vs. unlabeled post on *engagement intentions*, disaggregated by labeling condition. Effects are expressed in units of standard deviation and are estimated using linear regression models with stimulus fixed effects. 95% CI are based on robust standard errors. In all cases, negative values indicate that respondents assigned to a given label were less likely to say they would engage with the presented information, compared to respondents in the control group.

Next, we consider the impact of each label on participants' likelihood of interacting with AI-generated media. In contrast to the effects for beliefs, AI-centered labels do *not* meaningfully alter engagement intentions in either study (see Fig. 3). In Experiment 1, exposure to the “AI-Generated” label does not significantly affect individuals’ stated willingness to share [−0.04 SD (−0.15, 0.08)], “like” or favorite [−0.02 SD (−0.14, 0.09)], comment on or reply to [0.03 SD (−0.08, 0.15)], or seek out new information [−0.03 SD (−0.14, 0.08)] about their assigned post. Meanwhile, the three labels that do not directly reference AI all discernibly reduce self-reported sharing, “liking”/favoriting, and information-seeking.

In Experiment 2, the estimated effects for engagement tend to be smaller and less variable across the board (and, in many cases, are not statistically significant after adjusting for multiple comparisons; see SI Appendix, Section 4.3). However, process-based labels again appear to exert minimal influence on intended behavior. Exposure to the broad “Altered” label decreases people's interest in seeking out information about the post's topic [0.17 SD (−0.27, −0.06)], relative to the control group, but only slightly (and insignificantly) lowers their willingness to partake in various on-platform activities (sharing: −0.09 SD [−0.20, 0.01]; “liking”/favoriting: −0.06 SD [−0.16, 0.05]; commenting/replying: −0.02 SD [−0.13, 0.08]). Moreover, in line with Experiment 1, assignment to the standalone “AI-Generated” label has—at most—a small, insignificant impact on engagement intentions across all four outcome variables (sharing: −0.07 SD [−0.18, 0.04]; “liking”/favoriting: −0.03 SD [−0.14, 0.07]; commenting/replying: 0.03 SD [−0.08, 0.13]; information-seeking: −0.02 SD [−0.12, 0.08]).

Across studies, we therefore find that process-based labels reduce people's likelihood of believing the claims presented in a post without necessarily shaping their stated propensity to engage with this content. This seeming disconnect between beliefs and behaviors when it comes to AI disclosures is notable, given that the design of the “AI-Generated” label most closely resembles the current labeling strategies of several major technology companies (15, 52). If the goal of labeling AI-generated content is to shape viewers' beliefs without shifting engagement patterns, this limited behavioral impact of process-based labels may in fact be desirable. However, if labeling is intended to discourage viewers from interacting with unreliable or inauthentic content, process cues may be insufficient on their own.

### Subjective evaluations

**Fig. 4.**
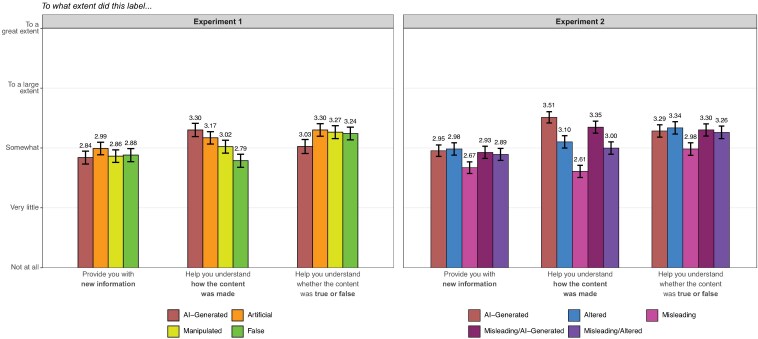
Average ratings of the informativeness of different types of labels. 95% CI on the mean are displayed. Note that these items were not asked of respondents in the unlabeled control group. A full summary of differences between these labels, including distributions of ratings on each measure, can be found in SI Appendix, Section 2.4.

Finally, although concerns about AI-generated misinformation often motivate calls for greater investment in labeling systems, the stated aim of AI labeling tends to be much simpler: to provide greater transparency about how content was created (11, 63). And, as summarized in Fig. 4, the labels we tested vary widely in this regard (see also SI Appendix, Figs. S7–S14). As expected—and in line with their intended purpose—process-based labels tend to provide more information about content's provenance. In both experiments, labels that explicitly identify images as AI-generated (i.e. the “AI-Generated” and “Misleading/AI-Generated” treatments) receive significantly higher average ratings for the extent to which they helped people understand how the content was made, compared to labels that predominantly focus on the veracity of the content, such as the “False” label in Experiment 1 (0.38 SD [0.27–0.49]) and the “Misleading” label in Experiment 2 (AIG: 0.65 SD [0.55–0.75]; Misleading/AIG: 0.53 SD [0.43–0.63]).

Similar, albeit muted, patterns also emerge for more generic process cues. In particular, labels divulging that content was edited or modified without explicitly identifying it as AI-generated (i.e. the “Artificial” and “Manipulated” labels in Experiment 1, the “Altered” labels in Experiment 2) seem to occupy a middle ground between conventional AI and fact-checking labels. Compared to the “AI-Generated” label, these labels tend to be less informative about how the content was made in both Experiment 1 (artificial: −0.10 SD [−0.21, 0.01]; manipulated: −0.21 SD [−0.32, −0.10]) and Experiment 2 (Altered: −0.30 SD [−0.40, −0.20]; Misleading/Altered: −0.37 SD [−0.47, −0.26]), though the differences in some cases are substantively small and statistically insignificant. However, they are significantly more informative, on average, than veracity-centered labels, including the “False” label in Experiment 1 (Artificial: 0.28 SD [0.17–0.39]; Manipulated: 0.17 SD [0.06–0.28]) and the “Misleading” label in Experiment 2 (Altered: 0.35 SD [0.24–0.45]; Misleading/Altered: 0.28 SD [0.18–0.38]). Together, these results suggest that process-based labels serve their aim of illuminating content's provenance, particularly when they specify the exact means of content creation (i.e. the use of AI).

By contrast, we observe fewer differences in the extent to which respondents say various labels helped them infer content's veracity. In Experiment 1, respondents report that the “AI-Generated” label was significantly less helpful than the others in conveying whether the presented content was true or false (Artificial: −0.20 SD [−0.31, −0.08]; Manipulated: −0.17 SD [−0.28, −0.05]; False: −0.15 SD [−0.26, −0.04]). However, this pattern does not emerge in Experiment 2, regardless of whether we examine the standard “AI-Generated” label (Altered: −0.03 SD [−0.13, 0.08]; Misleading: 0.21 SD [0.10, 0.31]; Misleading/Altered: 0.02 SD [−0.09, 0.12]) or the hybrid “Misleading/AI-Generated” label (Altered: −0.02 SD [−0.12, 0.09]; Misleading: 0.22 SD [0.12–0.33]; Misleading/Altered: 0.03 SD [−0.08, 0.13]). And respondents, on average, indicate that *all* the labels we tested at least somewhat helped them understand whether the content was true or false (see also SI Appendix, Figs. S12 and S14). These results add nuance to our earlier findings by suggesting that, when shown a warning label, individuals may draw conclusions about content's veracity (and adjust their beliefs accordingly), even in cases where the label does not explicitly flag the content as misleading.

## Discussion

Across two large-scale experiments, we show that labeling can meaningfully shape viewers' attitudes and behavior. On the whole, applying warning labels to posts containing misleading, AI-generated images reduces individuals' belief in and self-reported likelihood of sharing this content. Amid growing concern about generative AI's ability to catalyze the spread of deceptive or fraudulent content, our studies thus provide empirical evidence that labeling can shift the public's awareness of and receptivity to AI-generated images. But not all labels are created equal; instead, different types of labels convey different types of information. Most notably, AI disclosures, which indicate that content was created using AI, tend to elucidate how content was created without necessarily shifting individuals' likelihood of engaging with this content. As such, it may not be simply a matter of *whether* to label AI-generated content, but also a question of *how*. Rather than treat labeling as a one-size-fits-all solution, both scholars and practitioners should carefully consider the objectives that labeling is intended to accomplish and engineer solutions accordingly. Whereas process-based labels may be most appropriate in cases where the goal is simply to inform users about content's provenance, harm-based or “hybrid” labels may be better-suited to the specific case of AI-generated misinformation.

However, several limitations of these results bear mentioning. Although our use of survey experiments afforded several key benefits, it left open questions about how AI labels might perform in the real world. Here, we measured engagement intentions using self-reported items. Although we expect such survey items to be correlated with actual behavior (68, 69), they may not fully replicate everyday patterns of engagement. Relatedly, to limit participants' exposure to potentially deceptive content, we showed them a single post containing AI-generated media, but this content is typically situated within a much broader media ecosystem. Efforts to replicate our results in a more realistic setting, emulating the structure of a typical social media feed, may therefore furnish important insights. In this article, we also scoped our analysis to AI-generated misinformation—specifically, images that fact-checkers had previously flagged as misleading. Yet much of the AI-generated content in circulation today is meant for entertainment and creative expression, with little intent or capacity to deceive wide swaths of the public (70). Because AI labels are often applied to *all* detected images—not just those containing false claims—there is an urgent need for research clarifying whether, and in what ways, the effects of labeling vary for content that is vs. is not misleading. With these considerations in mind, studies incorporating a more diverse set of media (ideally in a field vs. survey setting) may enhance our understanding of how, and when, labeling shapes personal and societal outcomes.

Furthermore, our experimental results evince variability in how the public interprets and responds to different types of labels. In devising these labels, we sought to include a representative mix of options that platforms might reasonably consider—or, in some cases, have already adopted. However, we examined only a small set of candidate labels, leaving open the possibility that other approaches may be more (or less) effective than the ones used here. Both experiments also held constant the visual design of the labels: a small, relatively unobtrusive banner appearing below the image. In free-response comments, some respondents admitted they failed to spot this label at first glance. Such inattentiveness may only intensify when content is embedded in a larger social media feed or list of search results (71). More prominent interventions (e.g. interstitial screens that initially obscure the relevant media) may be able to overcome some of these limitations (72), despite several drawbacks of their own (73, 74). The impact of different labels may also extend beyond the metrics we tracked here. Although we sought to include a wide range of attitudinal and behavioral outcomes, we did not explore perceptions of the *poster* vs. the *post*. It could be the case that different types of labels engender more or less positive sentiment toward the user who created or shared the content, based on the inferences audiences draw about the user's intentions.

The long-term consequences of labeling likewise merit additional investigation. In our studies, we used distractor tasks to induce distance between the presentation of the posts and the elicitation of factual beliefs and found that labeling continued to exert a meaningful effect on beliefs even after a brief delay. However, given the cross-sectional nature of our design, we cannot ascertain whether, and how, the effects of labeling decay over time; longitudinal studies tracking responses to labeling over an extended time frame may be helpful in this regard. Likewise, there remain outstanding questions of temporal validity. Our studies were conducted at a time when many members of the public were broadly unfamiliar with generative AI; it is possible that consumers may come to distinguish different labels more clearly in a world where generative AI is more widely used and understood. In a similar vein, the present work, which exposed people to a single post containing an unfamiliar label, cannot probe potential dosage effects. Past research suggests that labels may have diminishing returns over time and across repeated exposures (75, 76). This possibility requires careful consideration, given its direct policy implications—for example, regarding the breadth of content to label (e.g. whether to label all AI-generated media vs. only posts that have been deemed especially “high-risk,” as well as focusing just on images as opposed to video or audio).

In addition, labels may not be equally effective across all individuals. As shown in SI Appendix, Section 3.3, we find some evidence of heterogeneity across subpopulations (e.g. by digital literacy, age), though the differences tend to be small and inconsistent across studies. However, it is important to recognize that our surveys were solely fielded in the United States with English-language posts. Although past work uncovers few cross-national differences in the *comprehensibility* of labels (50), individuals' *responses* to these labels may nonetheless vary widely across settings. Given the global scope of generative AI, there is a pressing need for additional work examining the effects of labeling outside the United States.

Lastly, our experiments elide the crucial question of which pieces of media to label. Here, we could be confident that the content we presented was both AI-generated and misleading. In practice, establishing provenance and veracity is much more difficult (70, 77). Ongoing efforts to improve the traceability of online media may ease some of these challenges, but a large swath of content may still evade detection. This under-provision of labels could have unintended consequences, beyond simply leaving a subset of AI-generated media unlabeled. Most notably, the addition of warning labels to a limited subset of cases might inadvertently boost the credibility of relevant content that goes unlabeled (an “implied authenticity” effect, in line with (36); though see (62)). From this perspective, a fragmented approach to labeling may have the perverse consequence of increasing trust in altered media by implying that unlabeled content has been verified as authentic. At the same time, previous research suggests that the misapplication of warning labels to reliable content could degrade trust in authentic information (78), whether such false positives are due to imperfect detection systems or fraudulent self-disclosure (79). Given these challenges, it is imperative for future work to assess how our results extrapolate to more complex, ambiguous media environments.

Even setting aside these limitations, labeling may not be a panacea, particularly when confronting the emerging threat of AI-generated misinformation. Though we find that warning labels can (at least temporarily) reduce individuals' belief in and engagement with manipulated media, our effect sizes are relatively modest in absolute terms—and, as noted above, may be even smaller in real-world settings where warning labels are less salient. If the end goal is combatting the spread of misinformation, labeling may benefit from being paired with other complementary enforcement mechanisms (e.g. removal or demotion of violating posts or account penalties for offending users). As generative AI tools continue to rapidly improve and evolve, technology companies face increased pressure to establish systems for mitigating the risks of these tools for individuals and society at large. In this article, we provide a model of how to design and test different labeling interventions and, in doing so, highlight important factors to bear in mind when developing these guardrails.

## Materials and methods

The hypotheses, sample recruitment process, and analysis plan for both experiments were preregistered prior to data collection (https://osf.io/7zgfm/?view_only=086e546010c144fb9749363398c8829c for Experiment 1; https://osf.io/d74a3?view_only=7bf7b5539a1f49c3b6b95210984e3c5e for Experiment 2). All respondents provided informed consent, and both experiments were deemed exempt by the Massachusetts Institute of Technology Committee on the Use of Humans as Experimental Subjects (protocol nos. E-5359 and E-5487). Due to our in-depth debriefing process, described below, respondents in the first experiment were not eligible to participate in the second.

### Survey samples

Our first survey experiment (Experiment 1) was administered online in October 2023, based on a preregistered sample size of 3,000 complete cases. Respondents were recruited via Lucid Marketplace, an online opt-in panel provider that uses quota sampling to obtain a nonprobability sample matching the US national distributions for age, gender, race/ethnicity, and geographic region. Overall, 4,427 respondents entered the survey, and 4,166 consented to participate. 3,396 of these respondents passed two trivial attention checks designed to detect random responding and were thus eligible to participate in the study. Of these respondents, 3,223 began the experimental task and are included in the final sample for Experiment 1.

We conducted a follow-up study (Experiment 2), again using Lucid Marketplace, in December 2023, with a preregistered sample size of 4,000 complete cases. Given the nature of our experimental design, individuals who participated in Experiment 1 were ineligible to participate in Experiment 2. In total, 6,817 respondents began the survey, and 6,091 consented to participate. Although not specified in our preanalysis plan, we excluded two cases where the same respondent completed the survey multiple times, retaining their first recorded response in both cases. Among the remaining respondents, 4,674 passed the same attention checks as in Experiment 1 and were therefore permitted to take part in the study. 4,425 of these respondents reached the experimental portion of the study; our final sample consists of 4,356 respondents who provided ratings for at least one of the dependent variables. Sample demographics are reported in SI Appendix, Table S1.

### Experimental procedure

After completing a series of pretreatment covariates (including personal demographics, political identification, social media use, and a five-item digital literacy scale), respondents were randomly assigned to view a screenshot of a social media post, drawn from a larger stimulus set. Each post contained an image that journalists or professional fact-checkers had previously identified as both AI-generated and misleading. These images were drawn from a diverse list of AI-generated media that had circulated on the Internet in the months prior to the study; we included 14 images in Experiment 1 and 29 in Experiment 2 (including the same 14 as in Experiment 1). In addition to the AI-generated image, each post included a text-based caption adapted from the original post in which the image first appeared online. In a number of cases, however, we adjusted the wording of this caption to make the claims clearer and more easily measurable using close-ended survey items.

For their assigned post, respondents were then randomized to one of several experimental conditions (for counts by treatment condition, see SI Appendix, Tables S5 and S6). In Experiment 1, respondents could be assigned to one of five groups. As described in the main text, respondents in the control group viewed a version of the post without a warning label attached. Respondents in the remaining conditions were instead shown a version of the post that displayed one of four warning labels (for exact wording, see SI Appendix, Fig. S1). Experiment 2 used a 3 × 2 factorial design, wherein we manipulated both whether respondents were exposed to one of two “process” cues explicating the process by which the image was created and whether they were assigned to a harm-based “veracity” cue flagging the image as misleading (see SI Appendix, Table S3 and Fig. S2).

After viewing the post, respondents first reported their likelihood of *engaging* with the presented content. Specifically, they were asked how likely they would be to (i) share, (ii) “like” or favorite, (iii) comment on or reply to, and (iv) seek out additional information about the post. In Experiment 1, to explore potential mechanisms, respondents were then asked to explain their rating for the sharing intentions item in their own words. Next, respondents in both experiments completed a series of distractor tasks, designed to shift respondents' attention away from the content of the post—and, where relevant, the attached label—before measuring their beliefs. In Experiment 1, the distractor tasks took the form of several measures of emotions and logical reasoning, including a six-item scale adapted from the Positive and Negative Affect Schedule (80), a measure of “need for cognition” (81), and a four-item Cognitive Reflection Test (82, 83). In Experiment 2, we instead included 19 gender-matched items from the Portrait Values Questionnaire (84), framed as measuring respondents' personality and values.

After completing the distractor tasks, respondents then indicated their *belief* in the post's core claims—for instance, whether they thought that Donald Trump had actually confronted NYPD officers while being arrested in New York, or whether they thought Joe Biden had actually converted to Buddhism. Respondents in Experiment 2 next rated five *characteristics* of the AI-generated image in their presented post, including the extent to which they thought it was (i) believable, (ii) accurate, (iii) authentic, (iv) manipulated, and (v) innovative, recoded such that higher ratings in all cases indicated more positive assessments of the image. Following the preanalysis plan, we constructed a summary measure of image credibility by taking a simple mean of responses to the first four attributes (Cronbach's *α*  *=* 0.79), in addition to evaluating each of these items independently (see SI Appendix, Fig. S6).

Lastly, respondents in each of the labeling conditions were asked to share their opinions about the label to which they were assigned, for use in exploratory analyses. First, respondents appraised several *attributes* of the label, summarized in SI Appendix, Section 2.4. In addition, they were asked about the *types of information* the label conveyed—namely, the extent to which it (i) provided them with new information, (ii) helped them understand how the content was made, and (iii) helped them understand whether the content was true or false. Respondents in Experiment 2 were finally asked to describe, in their own words, how they interpreted the label. All respondents were then extensively debriefed, as described below. The outcome variables were all measured using five-point Likert-style scales, and the exact question wording can be found in SI Appendix, Section 1.4.

### Analytical strategy

Following our preanalysis plans, we estimate treatment effects using linear regression models with heteroskedastic-robust standard errors (“HC2” variant). We fit all of our models using the estimatr package in R (85). In both experiments, we examine two quantities of interest. First, we compare respondents assigned to view labeled vs. unlabeled content, pooling our different labeling treatments together (see SI Appendix, Fig. S3). To do so, we regress our outcome variables on an indicator of whether respondents were assigned to one of the labeling conditions (coded as 1) or the unlabeled control group (coded as 0).

Next, we estimate the effects for each individual label (e.g. “AI-Generated” or “False”; for average ratings by label, see SI Appendix, Figs. S4 and S5). To do so, we run models that include dummy variables for each of the unique labeling conditions. For the main model specifications, we set the control condition as the reference group but modify this baseline category, as necessary, to facilitate direct comparisons between labels. Additionally, for Experiment 2, we estimate models that interact indicators of (i) the *process* cue respondents received (i.e. “AI-Generated,” “Altered,” or no cue) and (ii) the *veracity* cue respondents received (i.e. “Misleading” or no cue). In both sets of models, we include stimulus fixed effects, given that respondents were randomly assigned to view a single post from the broader stimulus set. We also re-estimate all quantities using Bayesian linear mixed-effect models that allow the intercept and treatment effects to vary across posts and obtain substantively identical results (SI Appendix, Figs. S28 and S29 and Table S14). In addition, following the preanalysis plan, we assess the robustness of our results to differential attrition (SI Appendix, Figs. S30–S33 and Tables S15–S18) and after adjusting for multiple comparisons (SI Appendix, Figs. S34–S36). Altogether, we observe limited variation in drop-out rates across experimental conditions. When such differences do emerge, they tend to be relatively small in magnitude and contained to the “Misleading/Altered” label in Experiment 2; as such, it is unlikely that our key results are solely the product of differential attrition.

### Treatment effect heterogeneity

To explore heterogeneous treatment effects across content types and respondent subpopulations, we fit a series of moderator models, described further in SI Appendix, Section 3 (Figs. S15–S27 and Tables S9–S13). All respondent-level variables, including *age*, *digital literacy*, *partisanship*, *social media use*, and survey *attentiveness*, were collected pretreatment (see SI Appendix, Section 1.4). To measure post-level attributes, we used a two-pronged approach. For each experiment, we first used baseline ratings among respondents in the control group to benchmark the believability of the unlabeled posts. To supplement the available data, we also collected external ratings of each post using a separate survey (deemed exempt by the MIT COUHES, protocol no. E-5504).

For this survey, respondents (*n* = 525; 1,546 total observations) were again recruited via Lucid Marketplace in December 2023, using the same quota sampling and screening procedures as in our prior experiments. To ensure respondents' perceptions were uncontaminated by prior exposure to one of our labeling treatments, we prevented subjects from both experiments from participating in this survey. After providing informed consent and completing two attention checks, each participant was randomly assigned to view three (unlabeled) social media posts, drawn from the 29 stimuli included in our experiments. For each post, participants completed four sets of questions. First, they were asked to rate eleven characteristics of the image, capturing perceptions of credibility, interestingness, and informativeness. Second, to gauge social salience, participants indicated how important it was for people to know about the information in the post. Third, participants indicated their belief that the events shown in the post actually happened and their prior familiarity with the post. Finally, participants were asked to classify whether the post was political and, if so, whether it favored one party over another. The full wording of these survey items can be found in SI Appendix, Section 3.1, and a more detailed description of our analytic strategy can be found in SI Appendix, Sections 3.2 and 3.3.

### Ethical considerations

We took a number of steps to minimize the potential consequences of exposure to AI-generated misinformation over the course of our studies, especially among respondents assigned to the control group (who were not initially informed that the presented content was synthetic). First, we thoroughly debriefed respondents at the end of each survey about the purpose of the experiment, during which time we also provided links to detailed fact-checking information about the specific image respondents were shown. Following (27), we adopted an active debriefing strategy, wherein respondents had to explicitly declare (in writing) that they understood the content they were shown was fake.

Second, we consciously selected experimental stimuli that we anticipated would pose limited risk of direct harm to viewers. Rather than add to the growing supply of AI-generated misinformation currently in circulation, we chose to focus on existing images that had already been covered in the mainstream media and/or reviewed by professional fact-checkers. In addition, we did not include any images we thought might be especially persuasive and/or detrimental for viewers' well-being, such as posts that were related to active international conflicts, could incite concerns about national security, contained serious allegations of personal misconduct, or included discriminatory language or imagery.

Finally, given particular concerns about the ability for AI-generated misinformation to sway electoral outcomes, we sought to balance our political stimuli by partisanship by including an approximately equal number of posts depicting Democratic and Republican figures. In addition, though much of the AI-generated misinformation reviewed by fact-checkers relates to Donald Trump and Joe Biden, we included a wide range of other political elites (e.g. Rand Paul, Cory Booker), in recognition of the electoral climate at the time of fielding. By taking these steps, we sought to balance the potential risks associated with exposing individuals to misinformation against the potential benefits of the research to individuals and society at large.

## Supplementary Material

pgaf170_Supplementary_Data

## Data Availability

All materials, data, and code needed to replicate the analyses for both experiments are available on the Open Science Framework: https://osf.io/f8gqp/ (86).

## Preprint

This manuscript was previously posted as a preprint: https://osf.io/preprints/psyarxiv/b238p.
