# Supplementary material for: Labeling AI-generated media online
Source: PNAS Nexus. 2025 May 28;4(6):pgaf170. doi: 10.1093/pnasnexus/pgaf170 (PMC12166545; doi:10.1093/pnasnexus/pgaf170)
Supplement: pgaf170_Supplementary_Data [file pgaf170_supplementary_data.pdf]

Supporting Information:

"Labeling AI-Generated Media Online"

Contents

1 Experimental Design **S-1**
1.1 Sample Demographics . . . S-1
1.2 Social Media Posts . . . S-2
1.3 Labeling Treatments . . . S-3
1.4 Survey Instrumentation . . . S-5

2 Overall Results **S-13**
2.1 Aggregate Effects of Labeling . . . S-13
2.2 Differences Across Labels . . . S-14
2.3 Interactive Models . . . S-18
2.4 Evaluations of the Label . . . S-20

3 Treatment Effect Heterogeneity **S-29**
3.1 External Ratings Task . . . S-29
3.2 Post-Level Moderators . . . S-33
3.3 Individual-Level Moderators . . . S-41

4 Robustness Checks **S-50**
4.1 Multi-Level Models . . . S-50
4.2 Balance and Attrition Checks . . . S-55
4.3 Correction for Multiple Comparisons . . . S-63

1 Experimental Design

1.1 Sample Demographics

Table S1 summarizes self-reported sample demographics for Experiments 1-2, as well as the external ratings task in which participants were asked to evaluate several aspects of the (unlabeled) stimuli (see SI Appendix, Section 3.1). All reported variables were measured pre-treatment for the experiments, whereas these items appeared at the end of the survey for the ratings task. Missing data or cases where respondents indicated "Prefer not to say" are excluded. For measures of political identification, "leaners" are classified as partisans/ideologues.

**Variable**	**Category**	**Exp. 1**	**Exp. 2**	**Ratings Task**
Gender	Male	48.4%	46.5%	48.7%
	Female	51.0%	52.8%	50.7%
	Other Gender Identity	0.6%	0.7%	0.6%
Age	18-24	11.7%	11.5%	12.2%
	25-34	17.6%	17.9%	17.9%
	35-44	15.9%	15.8%	17.1%
	45-54	16.0%	16.1%	15.6%
	55-64	17.1%	17.3%	16.4%
	65+	21.7%	21.4%	20.8%
Race/Ethnicity	White	63.9%	62.8%	-
	Black	11.8%	12.6%	-
	Hispanic/Latino	14.0%	14.2%	-
	Other Race/Ethnicity	10.3%	10.5%	-
Education	High School or Less	29.5%	31.5%	28.3%
	Some College	24.9%	24.2%	23.8%
	College Degree	32.0%	30.1%	30.7%
	Post-Graduate Degree	13.5%	14.2%	17.2%
Income	<$50K	51.5%	51.8%	46.4%
	$50-100K	28.4%	29.9%	30.8%
	>$100K	20.1%	18.3%	22.8%
Party ID	Independent	17.2%	20.4%	16.6%
	Democrat	45.1%	43.2%	45.5%
	Republican	37.8%	36.4%	37.9%
Ideology	Moderate	24.1%	28.5%	-
	Liberal	31.2%	31.4%	-
	Conservative	44.7%	40.1%	-
Device	Mobile	67.7%	57.9%	68.4%
	Tablet	1.5%	2.3%	1.9%
	Desktop	30.8%	39.8%	29.7%

Table S1: Sample demographics for Experiment 1 ($n = 3,223$), Experiment 2 ($n = 4,356$), and the image rating task ($n = 525$). Percents may not add up to 100 due to rounding. Note that several demographics items (race/ethnicity and ideological self-placement) were not included in the ratings task due to space constraints.

1.2 Social Media Posts

Table S2 provides an outline of the 29 posts included in our experiments; of these posts, 14 were included in both Experiments 1-2, and 15 were newly added in Experiment 2. We sourced these posts by locating mainstream media coverage and fact-checking articles focused on AI-generated images. Each image was accompanied by a brief, text-based caption that provided context for the events shown in the image. Where possible, we took these captions directly from the original posts in which the images appeared online, though in some cases we adjusted the language to facilitate measurement of our dependent variables. Each post also included a standard engagement bar, designed to mimic the format of posts appearing on 𝕏 (Twitter), but the posts were at no point identified as coming from a particular platform.

Image ID	Caption	Included in Exp. 1?
beached_shark	1,500-pound great white shark found on Outer Banks Beach in North Carolina. A team of onlookers is doing their best to push it back into the ocean.	✓
biden_bubble	NASA and the White House have designed an innovative new suit to protect Joe Biden from falls.	✓
biden_buddhism	Joe Biden announced today that he's converting to Buddhism.	
biden_celebrate	Photos show Joe Biden and Kamala Harris having a dance party in the White House to celebrate Donald Trump's indictment.	✓
booker_dress	Sen. Cory Booker (D-NJ) criticized for wearing tight pink shorts at the Capitol after a change to the Senate's dress code policy.	✓
cruise_doubles	Tom Cruise pictured with stunt doubles at the Mission Impossible 7 premiere after-party.	
dalai_arrest	BREAKING: Dalai Lama, the spiritual leader of Tibet, arrested and led away by police.	
hybrid_turtle	New species found in Santa Clara, Peru believed to be a prehistoric hybrid of a turtle and an octopus.	✓
johnson_arrest	BREAKING: Former UK Prime Minister Boris Johnson arrested in London.	
lobster_caught	Puerto Rican fishermen have caught the world's largest lobster, weighing over 300 pounds.	
melania_arrest	Melania Trump shouted in protest after being surrounded by police during her husband's arrest in Manhattan.	
moon_landing	CONFIRMED: Stanley Kubrick faked the "moon landing." Space is made in a Hollywood studio.	✓
musk_aoc	BREAKING: Elon Musk spotted out and about with AOC.	
musk_barra	BREAKING: Elon Musk now dating General Motors CEO Mary Barra.	✓
musk_robot	Elon Musk and his company are in the final stages of making a "robot wife."	
obama_pregnancy	Michelle Obama unveils glowing pregnancy photos after announcing she and Barack are expecting a third child.	✓
paris_trash	This is what Paris looks like today. The entrance to the Louvre is blocked by garbage after mass protests against police brutality.	✓
paul_dress	Sen. Rand Paul (R-KY) showed up to work at the Capitol barefoot in a red bathrobe after Senate dress code change.	✓
pence_pride	Former Vice President Mike Pence expresses support for the LGBTQ+ community, joins a local Pride parade.	✓
putin_arrest	Breaking News: Russian President Vladimir Putin has been arrested.	
putin_collapse	Vladimir Putin suffered a heart attack. Authorities say the Russian president is in critical condition after collapsing during a late-night meeting.	
royal_reunion	Prince William welcomes Prince Harry home at King Charles's coronation! Brothers finally reconcile after long feud.	
satan_pope	Pope Francis criticized after meeting with satanic priests in Bergamo, Italy.	
satan_school	An elementary school in Virginia just started a publicly funded after-school program to teach kids about Satanism.	✓
satan_target	They're Targeting Our Children: Target is now selling children's clothes with satanic imagery.	
trump_arrest	Former President Donald Trump confronts NYPD officers while being arrested outside Trump Tower.	✓
trump_crying	Former President Donald Trump seen crying in courtroom.	✓

Image ID	Caption	Included in Exp. 1?
trump_fauci	Donald Trump and Anthony Fauci are much closer than you'd think! Photos show Trump embracing the former White House chief medical advisor.	
walmart_tipping	Walmart is now asking for tips at self-checkout!	

Table S2: Description of the AI-generated images included in our experiments. 14 of the 29 images, tagged above, were included in both studies. The remainder of the images were solely included in Experiment 2. For each image, we provided a link to a fact-checking resource at the end of the study as part of our debriefing materials.

1.3 Labeling Treatments

Experiment 1

In the first study, respondents could be assigned to one of four labeling conditions or an unlabeled control group. The wording of each label can be found in Figure S1. In all cases, these labels appeared directly under the AI-generated image, following the example shown in Figure 1. We selected these four labels to include a diverse but representative mix of process- and harm-based labels. To enhance their ecological validity, we also based the wording and appearance of these labels on existing labeling policies and programs currently in place at leading social media companies. Finally, we sought to include a range of process- and harm-based cues (and combinations thereof). At one end of the spectrum, the "AI-Generated" label was purely focused on how the content was created—and specifically called out the use of generative AI. The "Artificial" label, by comparison, was intended to convey a more generic process cue while also providing some signal about the content's potential to mislead (following prior research; see [1]). Conversely, the "Manipulated" label was expected to primarily convey the content's misleadingness, while also referencing the process by which it was created (for additional information, see again [1]). Lastly, the "False" label served as a traditional fact-checking label, focused solely on the content's veracity.

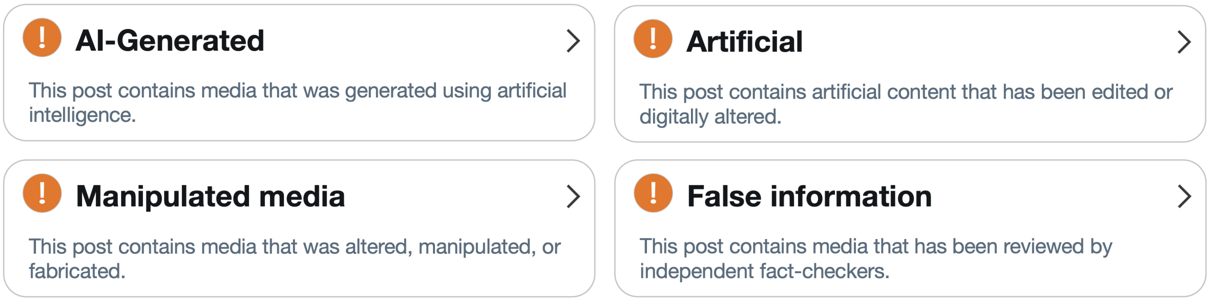

Figure S1: Wording of the labels used in Experiment 1. In all cases, these labels appeared immediately under the AI-generated image and above a standard engagement bar.

Experiment 2

As described in the main manuscript, the second study employed a 3 × 2 fully-crossed factorial design, wherein respondents could be assigned to one of six experimental conditions. The first factor of this design corresponded to the presence or absence of a *process* cue—namely, whether respondents received a *specific* cue that the image was "generated using artificial intelligence," a *broad* cue that the image had been "edited or digitally altered," or no information about the process by which the image was made. The second factor of this design corresponded to the presence or absence of a harm-based *veracity* cue—namely, whether or not respondents received a cue that the image "could mislead people."

This approach yielded the six experimental conditions summarized in Table S3. As in Experiment 1, respondents in the control group were shown a version of the post without any label applied. The remaining respondents were shown one of the five labels in Figure S2. We consider the "AI-Generated" and "Altered" labels to be purely process-based, as they solely convey information about the technical means by which the content was produced. In contrast, we consider the "Misleading" label purely harm-based, in that it only notes that the content could be misleading. Finally, the "Misleading/AI-Generated" and "Misleading/Altered" treatments are what we term "hybrid" labels, in that they combine both sets of cues.

		Veracity cue	
		**No veracity cue**	**Misleading cue**
Process cue	**No process cue**	Unlabeled	Misleading
	**Specific cue**	AI-Generated	Misleading/AI-Generated
	**Broad cue**	Altered	Misleading/Altered

Table S3: Description of the 3 × 2 factorial design in Experiment 2.

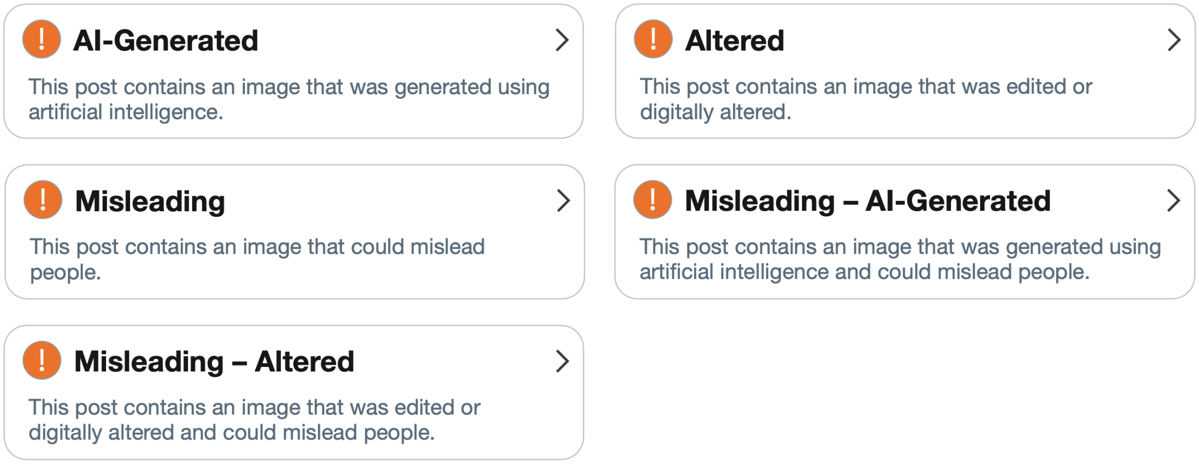

Figure S2: Wording of the labels used in Experiment 2. In all cases, these labels appeared immediately under the AI-generated image and above a standard engagement bar.

1.4 Survey Instrumentation

Pre-Treatment Items

Here, we provide the exact question wording for several pre-treatment covariates used in the exploratory moderator analyses summarized in Section 3.3. Except where otherwise noted, all questions had the same wording and were presented in the same order for Experiments 1-2.

- **Age:** although we used a measure of respondent age (in years) provided by Lucid to assess treatment effect heterogeneity, we validated this external measure using respondents' self-reported year of birth in the survey.
  - "In what year were you born?" *(Selected from a dropdown menu of years)*
- **Partisanship:** we assessed party identification using a standard two-stage branching item, which we re-coded into both a three- and seven-point measure (the former of which classified "leaners" as partisans).
  - Initial item: "Generally speaking, do you consider yourself a..."

    (1 = Democrat, 2 = Republican, 3 = Independent, 4 = Other (please specify))
  - Strength: *(Asked only of respondents who self-identified as a Democrat or Republican)* "Would you call yourself a strong [Democrat/Republican] or a not very strong [Democrat/Republican]?"

    (1 = Strong [Democrat/Republican], 2 = Not very strong [Democrat/Republican])
  - Lean: *(Asked only of respondents who self-identified as Independent or another party)* "Do you think of yourself as closer to the Republican Party or to the Democratic Party?"

    (1 = Closer to the Republican Party, 2 = Closer to the Democratic Party, 3 = Neither)
- **Social media use:**
  - Experiment 1: For the first experiment, we included a two-stage measure of social media use:
    - **Use:** "Which of the following social media sites do you **ever** use? *(Please select all that apply)*"

      (1 = Facebook, 2 = X (Twitter), 3 = Instagram, 4 = WhatsApp, 5 = Snapchat, 6 = YouTube, 7 = TikTok, 8 = Reddit, 9 = Other (please specify), 10 = None of the above; order of first eight options randomized)
    - **Frequency:** "About how frequently do you visit each of the following social media sites?"

      (1 = Never, 2 = A few times a month, 3 = About once a week, 4 = Several times a week, 5 = About once a day, 6 = Several times a day; asked only for sites respondents indicated using in the previous item)
  - Experiment 2: "Which of the following social media sites do you **regularly** use (at least once a week)? *(Please select all that apply)*"

  (1 = Facebook, 2 = X (Twitter), 3 = Instagram, 4 = WhatsApp, 5 = Snapchat, 6 = YouTube, 7 = TikTok, 8 = Reddit, 9 = Other (please specify), 10 = None of the above; order of first eight options randomized)
  - Large majorities of respondents in both experiments (88.2% and 90.7%, respectively) reported regularly visiting at least one social media site (weekly or more often).

- **Sharing behavior:** "Which of the following types of content would you consider sharing on social media (if any)? *(Please select all that apply)*"

  (1 = Political news, 2 = Sports news, 3 = Celebrity news, 4 = Science/technology news, 5 = Business news, 6 = Other (please specify), 7 = None of the above; order of first five options randomized)

  - For the analyses in Section 3.3, we re-coded this measure into a binary indicator of whether respondents said they would share any of the five types of news on this list.
  - Note: Responses in the "Other" category spanned a wide range of content types but commonly included things like personal news (e.g., updates about family, pets), religion and spirituality, and hobbies (e.g., cooking, crafting, or gardening)

- **Digital literacy:** we created a measure of digital literacy by calculating the proportion of correct answers (indicated with a *) to five close-ended questions assessing respondents' familiarity with computers, technology, and social media.

  - Instructions: "Now, we're interested in what you know about technology and social media."
  - Facebook Algorithm: "To the best of your knowledge, how are decisions made about what stories to show people on Facebook?"

    (1 = Based on a computer analysis of what stories might interest you, 2 = By editors and journalists who work for news outlets, 3 = By editors and journalists who work for Facebook, 4 = At random; order of options randomized)*
  - QR Code: "To the best of your knowledge, which of the following best describes what a 'QR Code' is?"

    (1 = An image made up of squares that can be scanned, 2 = A junk message sent to lots of people on the Internet, 3 = A four-number code that makes your device more secure, 4 = A website that can be edited by a group of people, 5 = A programming language for web pages, 6 = None of the above; order of first five options randomized)*
  - Download: "To the best of your knowledge, which of the following best describes what 'download' means?"

    (1 = A way to save files to your phone or computer, 2 = A way to change who can see the content you post online, 3 = A way to send a message from your phone to another device, 4 = A way to search for information (like news) on the Internet, 5 = A way to create something new from existing images, 6 = None of the above; order of first five options randomized)*
  - Tagging: "To the best of your knowledge, which of the following best describes what 'tag' means in the context of social media?"

(1 = A way to mention a specific individual, business, or account, 2 = A piece of information that circulates rapidly on the Internet, 3 = An image with text on it that describes a joke or idea, 4 = A sequence of instructions telling a computer what to do, 5 = A file that looks like a printed document or image, 6 = None of the above; order of first five options randomized)*

- Password: "To the best of your knowledge, which of the following four passwords is the most secure?"

  (1 = WTh@5Z, 2 = Boat1234, 3 = intro*48, 4 = 123456; order of options randomized)*

- **Attentiveness:** we constructed a measure of survey attentiveness based on the proportion of correct responses (indicated with a *) to two instructional manipulation checks embedded pre-treatment.

  - Interest screener: "People are very busy these days, and many do not have time to follow what goes on in the government. We are testing whether people read questions. To show you've read this much, please answer both 'extremely interested' and 'very interested.'"

    (1 = Not at all interested, 2 = Slightly interested, 3 = Moderately interested, 4 = Very interested, 5 = Extremely interested*)*

  - Web screener: "When a big news story breaks, people often go online to get up-to-the-minute details about what is going on. We want to know which websites people trust to get this information. We also want to know if people are paying attention to this question. To show that you've read this much, please ignore the question and select ABC News website and The Drudge Report as your two answers.

    When there is a big news story, which is the one news website you would visit first? *(Please only choose one)*"

    (1 = New York Times website, 2 = Huffington Post, 3 = Washington Post website, 4 = CNN.com, 5 = FoxNews.com, 6 = MSNBC.com, 7 = The Drudge Report, 8 = Google News, 9 = ABC News website*, 10 = CBS News website, 11 = NBC News website, 12 = Yahoo! News, 13 = The Associated Press (AP) website, 14 = Reuters website, 15 = National Public Radio (NPR) website, 16 = USA Today website, 17 = New York Post Online, 18 = None of these websites)*

Post-Treatment Items

After viewing their assigned image, respondents were asked to complete four sets of close-ended items, in the following order. Except where otherwise noted, the wording of these items was the same in both experiments.

- Engagement intentions: "If you saw this post online, how likely would you be to..."
  (1 = Not at all likely, 2 = Slightly likely, 3 = Moderately likely, 4 = Very likely, 5 = Extremely likely; order of statements randomized)
  - Share the post
  - "Like" or favorite the post
  - Comment on or reply to the post
  - Seek out additional information about the post's topic

Rather than measure beliefs immediately post-exposure, we first had respondents complete a series of distractor tasks, presented as measuring either their emotions and logical reasoning (in Experiment 1) or their personality and values (in Experiment 2).

- Experiment 1:
  - **Emotions:** "Now, we'd like to know more about your current emotions. Please indicate to what extent you feel the following emotions **right now**—that is, at the present moment."
    (1 = Not at all, 2 = Slightly, 3 = Moderately, 4 = Very, 5 = Extremely; order of emotions randomized)
    - Negative: Nervous, Distressed, Angry
    - Positive: Interested, Excited, Enthusiastic
  - **Need for Cognition:** "How well does the following statement describe you: 'I would rather do something that requires little thought than something that is sure to challenge my thinking abilities.'"
    (1 = Very untrue, 2 = Slightly untrue, 3 = Neither true nor untrue, 4 = Slightly true, 5 = Very true; order of response options randomly flipped)
  - **Cognitive Reflection Test:** "In the next part of the survey, you will be asked several questions. Please do your best to answer as accurately as possible."
    - "The ages of Mark and Adam add up to 28 years total. Mark is 20 years older than Adam. How many years old is Adam? *(Please enter a number)*"
    - "If it takes 10 seconds for 10 printers to print out 10 pages of paper, how many seconds will it take 50 printers to print out 50 pages of paper? *(Please enter a number)*"
    - "If you're running a race, and you pass the person in second place, what place are you in? *(Please enter a number and do **not** enter any letters)*"

  - "Emily's father has three daughters. The first two are named April and May. What is the third daughter's name?"

- Experiment 2:

  - **Preamble:** "**In the next part of the survey, we want to learn more about your personality and values.**

    We will briefly describe different people. Please read each description and think about how much that person is or is not like you. These descriptions will appear across the next **four** pages of the survey."

    - Respondents were asked to answer 19 different questions about their values; the order of these items was randomized both within and across four screens.
    - These items were gender-matched based on respondents' stated gender identity earlier in the survey. In cases where respondents did not self-report a gender, we used they/them pronouns throughout.

  - **Items:** "How much are the following people like you?" All of the answers then started with "It is important to [him/her/them]..."

    (1 = Not like me at all, 2 = Not like me, 3 = A little like me, 4 = Moderately like me, 5 = Like me, 6 = Very much like me)

    - To form [his/her/their] views independently.
    - To make [his/her/their] own decisions about [his/her/their] life.
    - To always to look for different things to do.
    - To have a good time.
    - To have ambitions in life.
    - That people do what [he/she/they] say[s] they should.
    - To have the power that money can bring.
    - That no one should ever shame [him/her/them].
    - To avoid disease and protect [his/her/their] health.
    - That [his/her/their] country is secure and stable.
    - To maintain traditional values and ways of thinking.
    - Never to violate rules or regulations.
    - To avoid upsetting other people.
    - Never to think [he/she/they] deserve[s] more than other people.
    - To care for nature.
    - That the weak and vulnerable in society be protected.
    - To be tolerant toward all kinds of people and groups.
    - To take care of people [he/she/they] [is/are] close to.
    - That people [he/she/they] know[s] have full confidence in [him/her/them].

Finally, respondents completed a second set of outcome variables focused on the believability of the image/post and the quality of the label.

- Belief in the presented claim: "To the best of your knowledge, **[insert claim]**?"
  (1 = Definitely did not happen, 2 = Probably did not happen, 3 = Not sure if did or did not happen, 4 = Probably happened, 5 = Definitely happened; order of response options randomly flipped)
  - The wording of the belief item was tailored to the displayed post. Table S4 provides the exact wording for each stimulus.
- Image attributes: Respondents in Experiment 2 were then re-shown their original post (with the label appended, where applicable), in order to probe perceptions of the image.
  - **Preamble:** "Earlier in the survey, we showed you the following social media post. We are curious to hear more about what you thought of this post."
  - **Attributes:** "To what extent is the image in this post..."
    (1 = Not at all, 2 = Slightly, 3 = Moderately, 4 = Very, 5 = Extremely; order of statements randomized)
    - Believable
    - Accurate
    - Authentic
    - Manipulated *(Reverse-coded)*
    - Innovative
- Evaluation of the label: Respondents assigned to a labeling condition were then asked to assess the quality of the label using two sets of items.
  - **Preamble:** "As you may have noticed, the post you were shown included a warning label indicating that the post contained **[insert description of label]**. We are interested in hearing your thoughts about this label."
    - The language for this preamble varied across labels (e.g., "AI-generated content" or "content that could be misleading"). Each question was also accompanied by a screenshot of the associated label.
  - **Attributes:** "In your opinion, to what extent was this label..."
    (1 = Not at all, 2 = Slightly, 3 = Moderately, 4 = Very, 5 = Extremely; order of statements randomized)
    - Clear
    - Informative
    - Helpful
    - Trustworthy
    - Biased *(Reverse-coded)*
    - Easy to understand *(Exp. 2 only)*

– **Information provision:** “To what extent did this label...”

(1 = Not at all, 2 = Very little, 3 = Somewhat, 4 = To a large extent, 5 = To a great extent; order of statements randomized)

- Help you understand how the content was made
- Provide you with new information
- Help you understand whether the content was true or false

In addition to these close-ended questions, we also included one open-ended probe in each study. In Experiment 1, immediately after the engagement intentions items, we asked respondents to explain in their own words why they provided the rating they did for the sharing measure. In Experiment 2, by contrast, we moved the open-ended probe to the end of the survey and instead asked respondents to say more about how they interpreted the label.

- **Experiment 1:** “On the previous page, you indicated that you would be [**insert rating**] to share the post online. Why did you provide this rating?

  *(Please be as specific as possible. We ask that you write **at least 25** characters of text.)*”

- **Experiment 2:** “Finally, we want to hear more about how you interpreted this label. What thoughts came to mind as you reviewed the label?

  *(Please be as specific as possible. We ask that you write **at least 25** characters of text.)*”

**Image ID**	**Belief Item** *(To the best of your knowledge...)*	**Included in Experiment 1?**
beached_shark	Did a 1,500-pound great white shark recently wash up on a beach in North Carolina?	✓
biden_bubble	Did NASA and the White House design a suit to protect Joe Biden from falls?	✓
biden_buddhism	Did Joe Biden convert to Buddhism?	
biden_celebrate	Did Joe Biden and Kamala Harris have a dance party in the White House to celebrate Trump’s indictment?	✓
booker_dress	Did Cory Booker wear a bright pink outfit to the Capitol in response to recent changes in the Senate’s dress code?	✓
cruise_doubles	Did Tom Cruise use stunt doubles for the latest Mission Impossible movie?	
dalai_arrest	Was the Dalai Lama recently arrested?	
hybrid_turtle	Was a prehistoric hybrid of a turtle and octopus recently discovered in Peru?	✓
johnson_arrest	Was former U.K. Prime Minister Boris Johnson recently arrested?	
lobster_caught	Did fishermen recently catch the world’s largest lobster in Puerto Rico?	
melania_arrest	Did Melania Trump confront police officers during her husband’s arrest in New York?	
moon_landing	Was the U.S. moon landing faked?	✓
musk_aoc	Did Elon Musk date U.S. Rep. Alexandria Ocasio-Cortez?	
musk_barra	Did Elon Musk date Mary Barra, the CEO of General Motors?	✓
musk_robot	Has Elon Musk’s company developed a “robot wife”?	
obama_pregnancy	Did Michelle Obama announce that she is pregnant with a third child?	✓
paris_trash	Did trash piles block the entrance to the Louvre in Paris following mass protests against police brutality?	✓
paul_dress	Did Rand Paul wear pajamas to the Capitol in response to recent changes in the Senate’s dress code?	✓
pence_pride	Did Mike Pence march in a local Pride parade in support of LGBTQ+ rights?	✓
putin_arrest	Was Russian President Vladimir Putin recently arrested?	
putin_collapse	Did Russian President Vladimir Putin recently have a heart attack?	
royal_reunion	Did Prince William and Prince Harry reconcile at King Charles’s coronation?	

Image ID	**Belief Item** *(To the best of your knowledge...)*	Included in Experiment 1?
satan_pope	Did Pope Francis meet with satanic priests in Italy?	
satan_school	Did a Virginia elementary school start an after-school program to teach children about Satanism?	✓
satan_target	Has Target started selling children's clothing with satanic imagery?	
trump_arrest	Did Donald Trump confront NYPD officers while being arrested outside Trump Tower?	✓
trump_crying	Did Donald Trump cry while appearing in court?	✓
trump_fauci	Did Donald Trump embrace Anthony Fauci, the former White House chief medical advisor?	
walmart_tipping	Has Walmart started requesting tips at self-checkout kiosks?	

Table S4: Exact wording of the belief item for each of the 29 experimental stimuli. This item always took the form, "To the best of your knowledge..." followed by the statements in the table. In cases where posts appeared in both Experiment 1 and 2, the question wording was held constant across studies.

2 Overall Results

2.1 Aggregate Effects of Labeling

Figure S3 plots the effects of exposure to labeling of any kind, relative to the unlabeled control group. In all cases, negative values indicate, in line with our pre-registered hypotheses, that respondents exposed to a labeled versus unlabeled post are less likely to believe and say they would engage with AI-generated misinformation. All treatment effects are expressed as standard deviations of the (five-point) outcome scale. On the whole, the results are directionally similar across studies but, in some cases, vary slightly in their magnitude (see also Figures 2 and 3). Though we cannot definitively attribute these differences to a single cause, it is possible that they reflect differences in the wording of the labels (Section 1.3), the posts included in the stimulus set (Section 1.2), the timing of the study, the composition of the sample (Section 1.1), or some combination of these and other factors. Replicating these results with a broader array of content and sample of labels may therefore be informative.

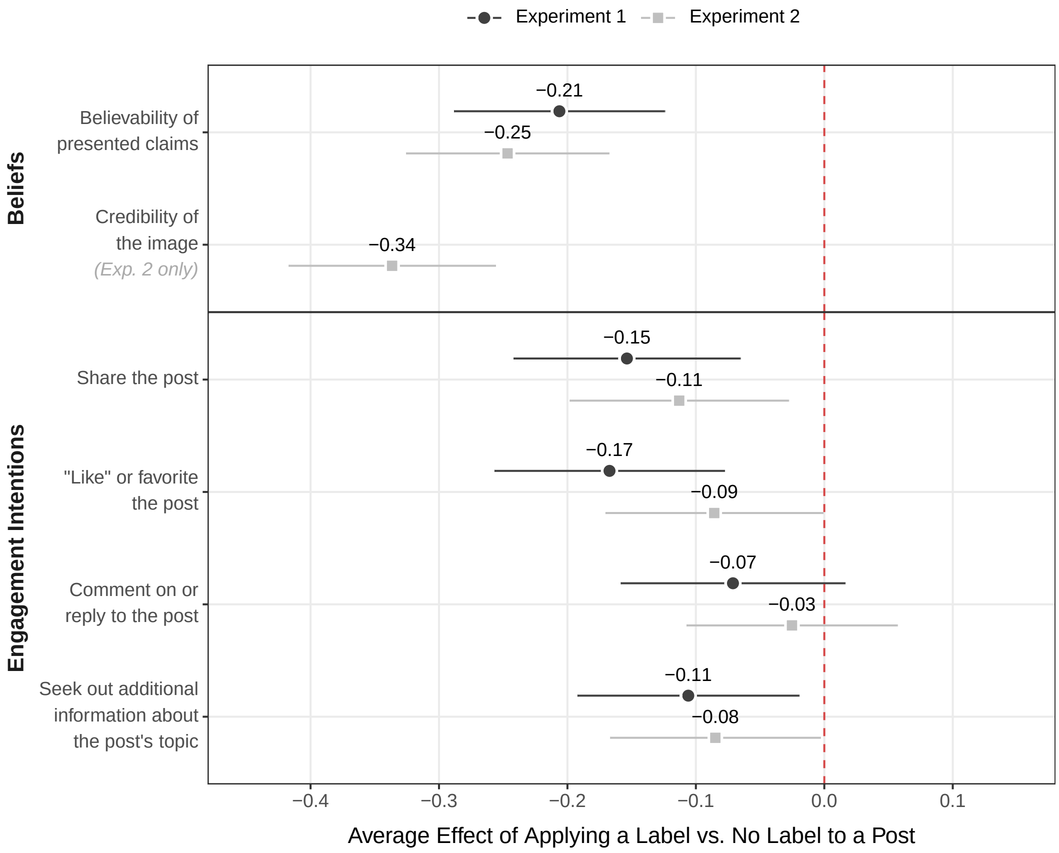

Figure S3: Average treatment effect of assignment to a labeled versus unlabeled post across experiments. Effects are expressed in units of standard deviation and are estimated using linear regression models with stimulus fixed effects. 95% confidence intervals are based on robust standard errors. In all cases, negative values indicate that respondents assigned to a label (of any kind) were less likely to believe or say they would engage with the presented information, compared to respondents in the control group. The perceived credibility of the image was only measured in Experiment 2.

2.2 Differences Across Labels

Counts by Condition

In Tables S5 and S6, we report the number of responses received in each experimental condition, for each of the outcome variables reported in the main text. Respondents were allocated to treatment conditions using simple random assignment, with equal probability of assignment to each of the conditions (not constraining the size of the treatment groups to be exactly equal). The wording of each outcome variable can be found in SI Appendix, Section 1.4. Note that respondents in the unlabeled control group were not asked any of the questions related to evaluations of the label (i.e., the `label_learn_*` variables). For a discussion of differential attrition, see Section 4.2. For readability, we use the following shorthand to designate each outcome variable:

- `engage_1`: Self-reported likelihood of sharing the post
- `engage_2`: Self-reported likelihood of "liking" or favoriting the post
- `engage_3`: Self-reported likelihood of commenting on or replying to the post
- `engage_4`: Self-reported likelihood of seeking out additional information about the post's topic
- `belief`: Belief in the post's claims (supported by the AI-generated image)
- `credible`: Perceived credibility of the image (based on four attributes, summarized in Section 1.4; *Experiment 2 only*)
- `label_learn_1`: Extent to which the label helped respondents understand how the content was made
- `label_learn_2`: Extent to which the label provided respondents with new information
- `label_learn_3`: Extent to which the label helped respondents understand whether the content was true or false

Condition	engage_1	engage_2	engage_3	engage_4	belief	label_learn_1	label_learn_2	label_learn_3
Control	634	632	633	632	606	-	-	-
AI-Generated	625	624	625	624	602	595	595	597
Artificial	633	636	635	635	609	603	603	604
Manipulated	668	667	667	667	641	641	640	640
False	649	651	648	649	622	615	614	615

Table S5: Count of responses received for each outcome variable, disaggregated by experimental condition, in Experiment 1. Note that respondents in the unlabeled control group were not asked any of the `label_learn_*` items. These items, along with the belief measure, were also asked after a distractor task.

Condition	engage_1	engage_2	engage_3	engage_4	belief	credible	label_learn_1	label_learn_2	label_learn_3
Control	679	679	675	680	656	650	-	-	-
AI-Generated	735	737	736	738	711	707	699	701	702
Altered	720	726	727	727	695	690	682	681	683
Misleading	715	717	717	715	695	688	684	683	684
Misleading/AIG	746	746	749	750	732	731	726	724	726
Misleading/Altered	719	718	721	725	699	694	691	691	691

Table S6: Count of responses received for each outcome variable, disaggregated by experimental condition, in Experiment 2. Note that respondents in the unlabeled control group were not asked any of the `label_learn_*` items. These items, along with the belief and credibility measures, were also asked after a distractor task.

Average Ratings

Figure S4 plots average ratings for the belief items, disaggregated by experimental condition, and Figure S5 plots average ratings for the measures of engagement intentions. As these plots indicate, respondents are generally more likely to express belief in the post's claims, rather than say they would engage with the post. Indeed, respondents in the unlabeled control group, on average, say they would be only slightly likely to share, "like"/favorite, or comment on/reply to the presented information. As shown in Section 3.3, we observe significantly higher rates of self-reported engagement among respondents who indicated pre-treatment that they would consider sharing various types of news content on social media, though the effects of labeling are not markedly different when comparing sharers and non-sharers. However, it is important to note that exposure to labeling reduces, but does not fully extinguish, viewers' belief in the presented claims. The full wording of the response scales is reported in Section 1.4.

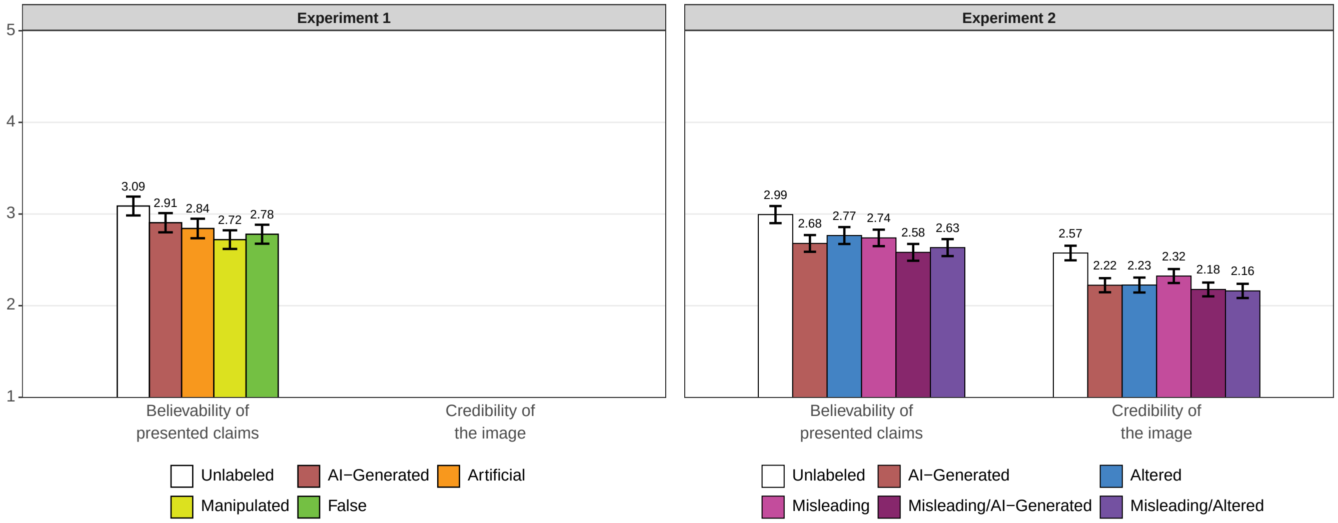

Figure S4: Average ratings of beliefs and perceived credibility across the two experiments. 95% confidence intervals on the mean are displayed. Both items were measured using five-point scales; the belief item ranged from "Definitely did not happen" to "Definitely happened," whereas the credibility index ranged from "Not at all" to "Extremely" credible, based on a simple mean of ratings of the extent to which the content was believable, accurate, authentic, and manipulated (where the final item was reverse-coded). The perceived credibility of the image was only measured in Experiment 2.

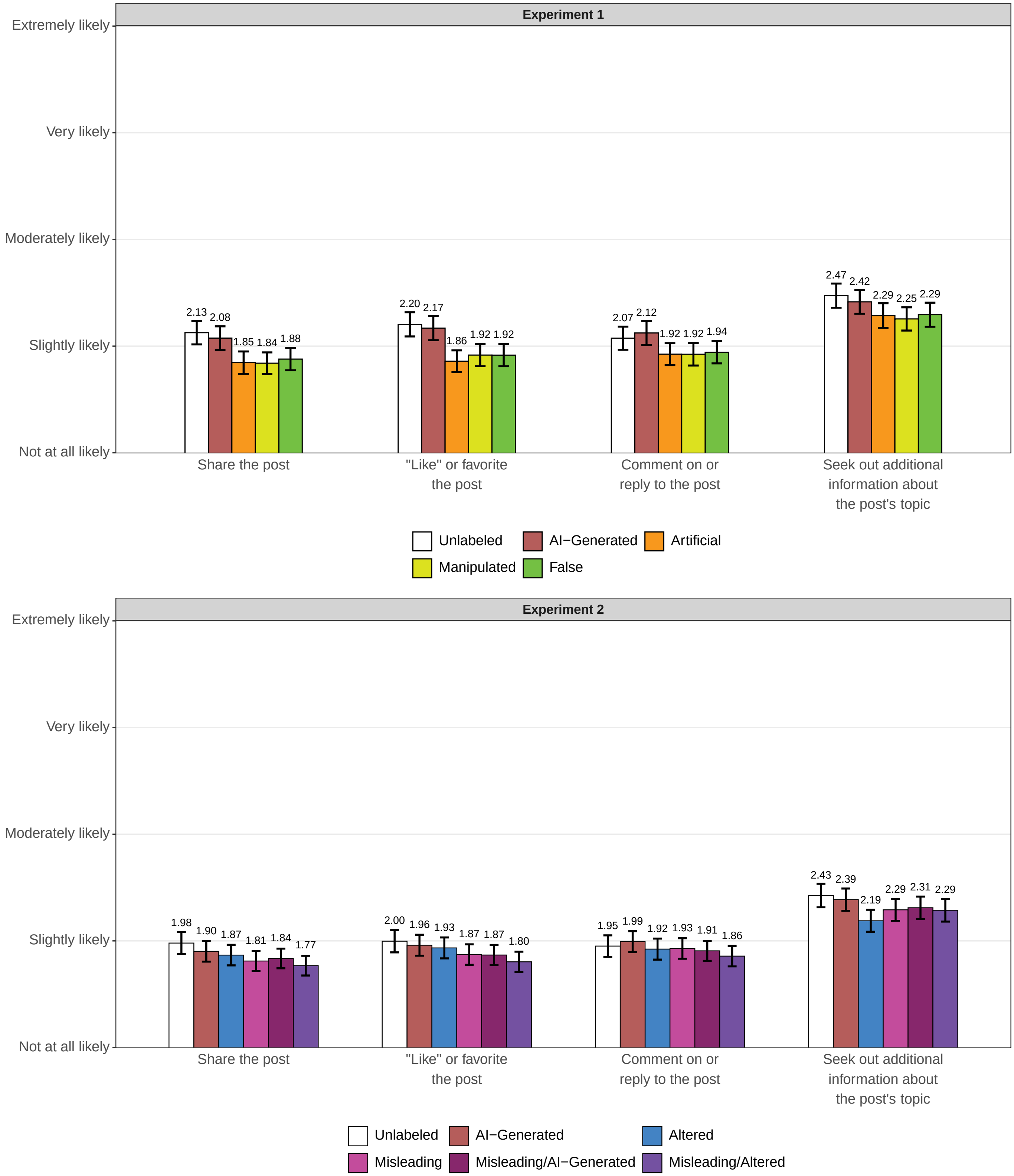

Figure S5: Average ratings of engagement intentions across the two experiments. 95% confidence intervals on the mean are displayed. All items were measured using five-point scales ranging from “Not at all likely” to “Extremely likely.”

Figure S6 presents treatment effects for the sub-components of the credibility index, as well as perceptions of innovativeness, in Experiment 2. Note that the standard deviation of the credibility index is smaller than for each of the individual items, which is reflected in the reported effect sizes, which are expressed in standard deviations of the outcome scale. Notably, the more muted effect of the "Misleading" label on the summary measure of credibility in Figure 2 seems to be more apparent for perceived *authenticity* and *manipulation*, perhaps because this label was the only one of the five that conveyed no information about the fact that the image had been digitally altered. Additionally, we find no evidence that labeling makes people view the images as more *innovative*, even when the labels convey that the content was AI-generated (and therefore that it was created using a relatively novel technology).

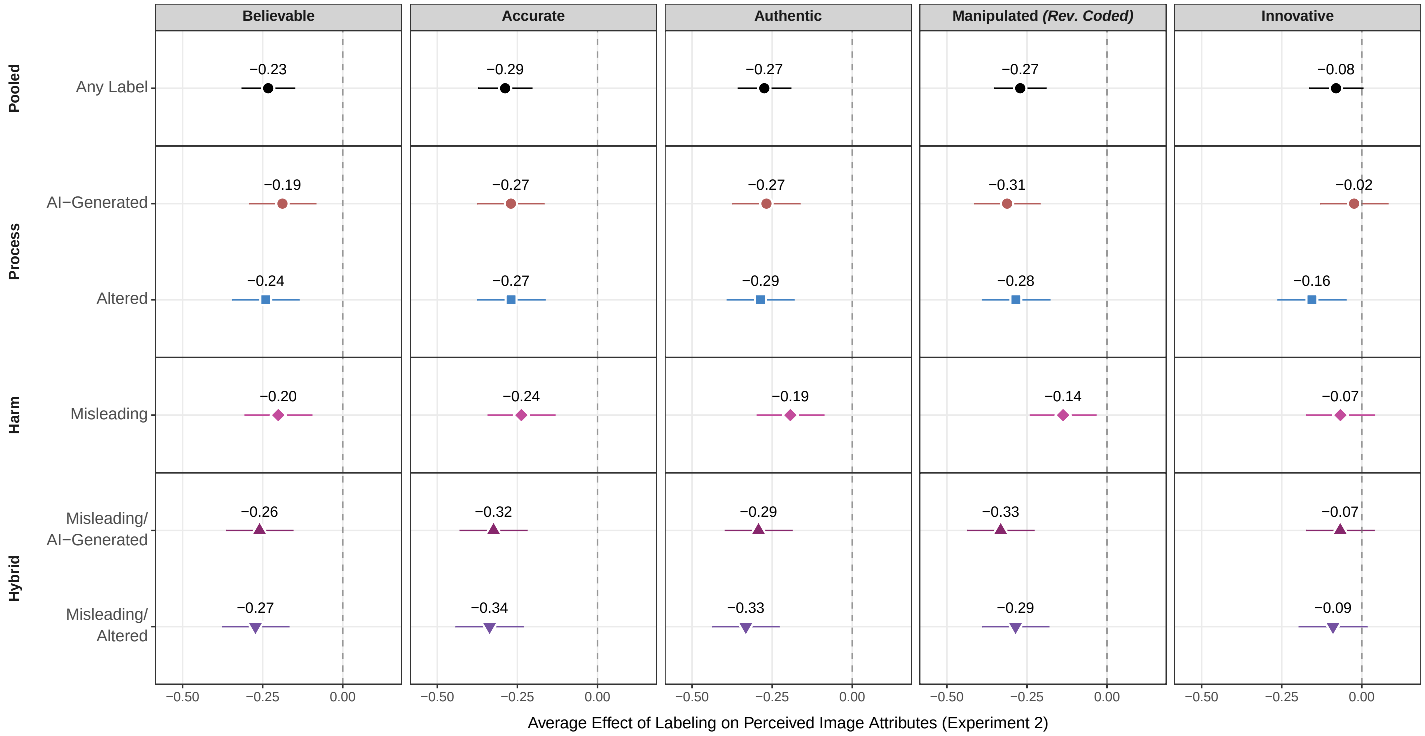

Figure S6: Average treatment effect of assignment to a labeled versus unlabeled post on perceived image attributes, disaggregated by labeling condition. Effects are expressed in units of standard deviation and are estimated using linear regression models with stimulus fixed effects. 95% confidence intervals are based on robust standard errors. In all cases, negative values indicate that respondents assigned to a given label were less likely to ascribe credibility or innovativeness to the presented image, compared to respondents in the control group. The measure of perceived manipulation is reverse-coded, such that higher ratings indicate that respondents viewed the image as *less* manipulated. Note that these items were only asked of respondents in Experiment 2.

2.3 Interactive Models

In Experiment 2, we systematically manipulate the presence or absence of two types of cues (*process* and harm-based *veracity* cues), using a 3 × 2 factorial design. This design allows us to assess the value of presenting these two sets of cues both alone and in concert. Specifically, Table S7 reports the estimated coefficients from models that interact dummy variables for assignment to a process cue (`ai` or `altered`) with a dummy variable indicating assignment to a veracity cue (`misleading`). In all cases, the dependent variables are standardized, and stimulus fixed effects are included. To facilitate interpretation of the coefficients, we center-code each of our dummy variables to have a mean of `0`. Because of this set-up, negative coefficients for the base terms indicate that assignment to a given type of cue, on average, decreases respondents' stated belief in and likelihood of engaging with AI-generated content. Negative coefficients for the interaction terms, by contrast, indicate that the provision of one cue amplifies the (negative) effect of the other on beliefs or engagement intentions. A version of these models with standard `1/0` dummy coding is also available in Table S8; the coefficients in this table directly map onto the disaggregated treatment effects presented in Figures 2-3.

When it comes to our measures of beliefs—including belief in the post's core claims and the perceived credibility of the image—we find, on average, that exposure to both types of cues decreases the believability of the presented content. However, these patterns are not consistently evident for engagement intentions. In particular, informing respondents that content is AI-generated has, on average, no discernible effect on their self-reported likelihood of sharing, "liking"/favoriting, commenting on/reply to, or seeking out more information about the post. Moreover, we observe minimal evidence of a multiplicative effect across our main outcome variables; in fact, for multiple measures, the interaction terms are instead positively signed. These results suggest that both process and veracity cues can shift individuals' beliefs and perceptions of message credibility, but the whole is not necessarily greater than the sum of its parts.

	Beliefs	Credibility	Share	Like/Favorite	Comment/Reply	Seek Info
AI-Generated	−0.181*	−0.235*	−0.027	−0.024	0.002	−0.005
	(0.036)	(0.036)	(0.038)	(0.037)	(0.037)	(0.037)
Altered	−0.122*	−0.240*	−0.064+	−0.056	−0.039	−0.081*
	(0.036)	(0.037)	(0.037)	(0.037)	(0.037)	(0.037)
Misleading	−0.139*	−0.113*	−0.080*	−0.083*	−0.043	−0.017
	(0.030)	(0.030)	(0.030)	(0.030)	(0.031)	(0.030)
AI-Generated × Misleading	0.104	0.190*	0.081	0.016	−0.047	0.029
	(0.072)	(0.073)	(0.075)	(0.075)	(0.074)	(0.074)
Altered × Misleading	0.097	0.190*	0.057	0.001	−0.029	0.169*
	(0.072)	(0.074)	(0.075)	(0.075)	(0.075)	(0.074)
Num. Obs.	4,188	4,160	4,314	4,323	4,325	4,335
Stimulus Fixed Effects?	✓	✓	✓	✓	✓	✓
R^2	0.101	0.080	0.022	0.026	0.017	0.036
R^2 Adj.	0.094	0.073	0.014	0.019	0.010	0.029

+ $p < 0.1$, * $p < 0.05$

Table S7: Estimates from models regressing each outcome on dummy variables for *process* and *veracity* cues. All dummy variables are center-coded (i.e., de-meaned) to facilitate interpretation of the base terms. All models incorporate stimulus fixed effects, and the dependent variables are standardized.

	Beliefs	Credibility	Share	Like/Favorite	Comment/Reply	Seek Info
AI-Generated	−0.233*	−0.331*	−0.068	−0.033	0.025	−0.020
	(0.052)	(0.052)	(0.055)	(0.054)	(0.054)	(0.053)
Altered	−0.171*	−0.337*	−0.093+	−0.057	−0.024	−0.167*
	(0.051)	(0.053)	(0.055)	(0.054)	(0.054)	(0.053)
Misleading	−0.207*	−0.242*	−0.127*	−0.089+	−0.017	−0.083
	(0.051)	(0.052)	(0.054)	(0.054)	(0.053)	(0.053)
AI-Generated × Misleading	0.104	0.190*	0.081	0.016	−0.047	0.029
	(0.072)	(0.073)	(0.075)	(0.075)	(0.074)	(0.074)
Altered × Misleading	0.097	0.190*	0.057	0.001	−0.029	0.169*
	(0.072)	(0.074)	(0.075)	(0.075)	(0.075)	(0.074)
Num. Obs.	4,188	4,160	4,314	4,323	4,325	4,335
Stimulus Fixed Effects?	✓	✓	✓	✓	✓	✓
R^2	0.101	0.080	0.022	0.026	0.017	0.036
R^2 Adj.	0.094	0.073	0.014	0.019	0.010	0.029

+ $p < 0.1$, * $p < 0.05$

Table S8: Estimates from models regressing each outcome on dummy variables for *process* and *veracity* cues. All dummy variables are coded as `1` if a respondent received that cue, `0` otherwise. All models incorporate stimulus fixed effects, and the dependent variables are standardized.

2.4 Evaluations of the Label

To aid in exploratory analyses, we asked respondents assigned to a labeling condition to provide their opinions about the label they were shown. First, we asked respondents in both experiments to rate the label along three dimensions:

- **Clarity:** Clear, Easy to understand *(Exp. 2 only)*
- **Utility:** Informative, Helpful
- **Credibility:** Trustworthy, Biased

In all cases, the scale ranges from "Not at all" to "Extremely," where higher ratings indicate that respondents were more likely to ascribe that trait to their assigned label. As such, higher ratings on this scale correspond to more positive ratings for all of the measures—except for whether the label was "Biased" (given the negative valence of this item).

Figure S7 summarizes average ratings on these items, disaggregated by labeling condition, and Figure S8 plots the average treatment effect of assignment to the "AI-Generated" label, relative to another label, for these same measures. Respondents by and large rate the labels as moderately clear and easy to understand. But in Experiment 1, we find that respondents view the "AI-Generated" label as marginally less useful (based on the extent to which it was perceived as both "informative" and "helpful") and trustworthy than the other three. In Experiment 2, the effects are somewhat more mixed. On the one hand, the "AI-Generated" label is viewed as slightly clearer and easier to understand, compared both to the "Misleading" label and, interestingly, the hybrid "Misleading/AI-Generated" label. On the other hand, as in Experiment 1, this label tends to be perceived as less trustworthy than the two "Altered" labels.

Figures S9 plots analogous effects for a measure of the different *types of information* each label provided, as a complement to the average ratings shown in Figure 4. As noted in the main text, respondents in Experiment 1 find the AI label more informative about how the content was made, compared to the other labels, but less informative about whether it was true or false. In Experiment 2, labels containing process cues—including "hybrid" treatments—all tend to be perceived as similarly informative about content's veracity, but the standalone AI label continues to be seen as most informative about how the content was made.

Label Attributes

Figure S7 summarizes average ratings of label attributes for each treatment condition. Figure S8 then presents the estimated treatment effect of assignment to the "AI-Generated" condition, relative to one of the other labels, for these same measures; in all cases, positive values indicate that respondents were, on average, *more* likely to ascribe a given attribute to the AI label, whereas negative values indicate that respondents were *less* likely to ascribe the attribute to the AI label. Note that we only measured perceptions of the extent to which the label was "easy to understand" in Experiment 2.

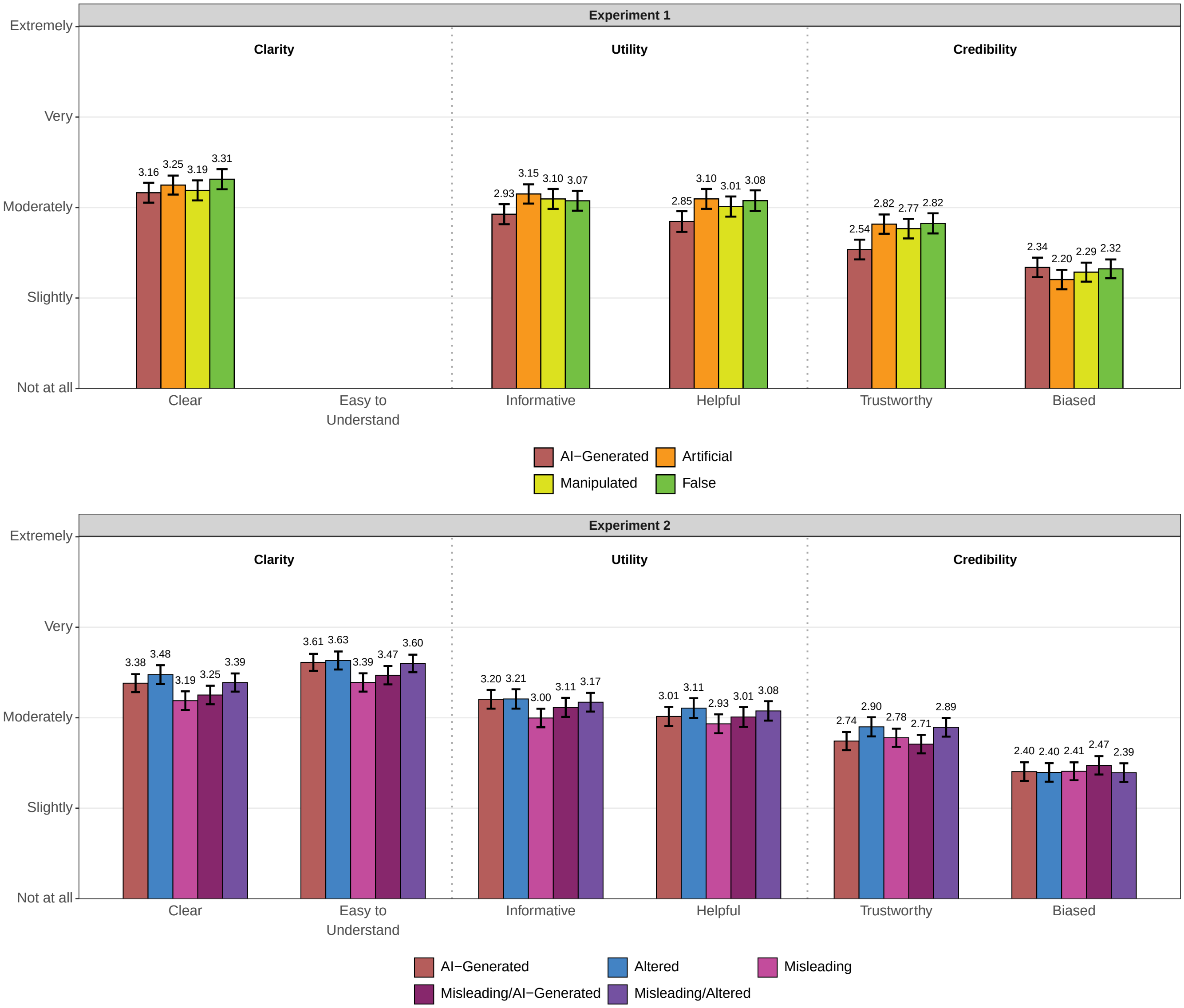

Figure S7: Average ratings of perceived label attributes across the two experiments. 95% confidence intervals on the mean are displayed. All items were measured using five-point scales, where higher ratings indicate that respondents were more likely to ascribe a given trait to their assigned label. Respondents were only asked about the extent to which the label was "easy to understand" in Experiment 2.

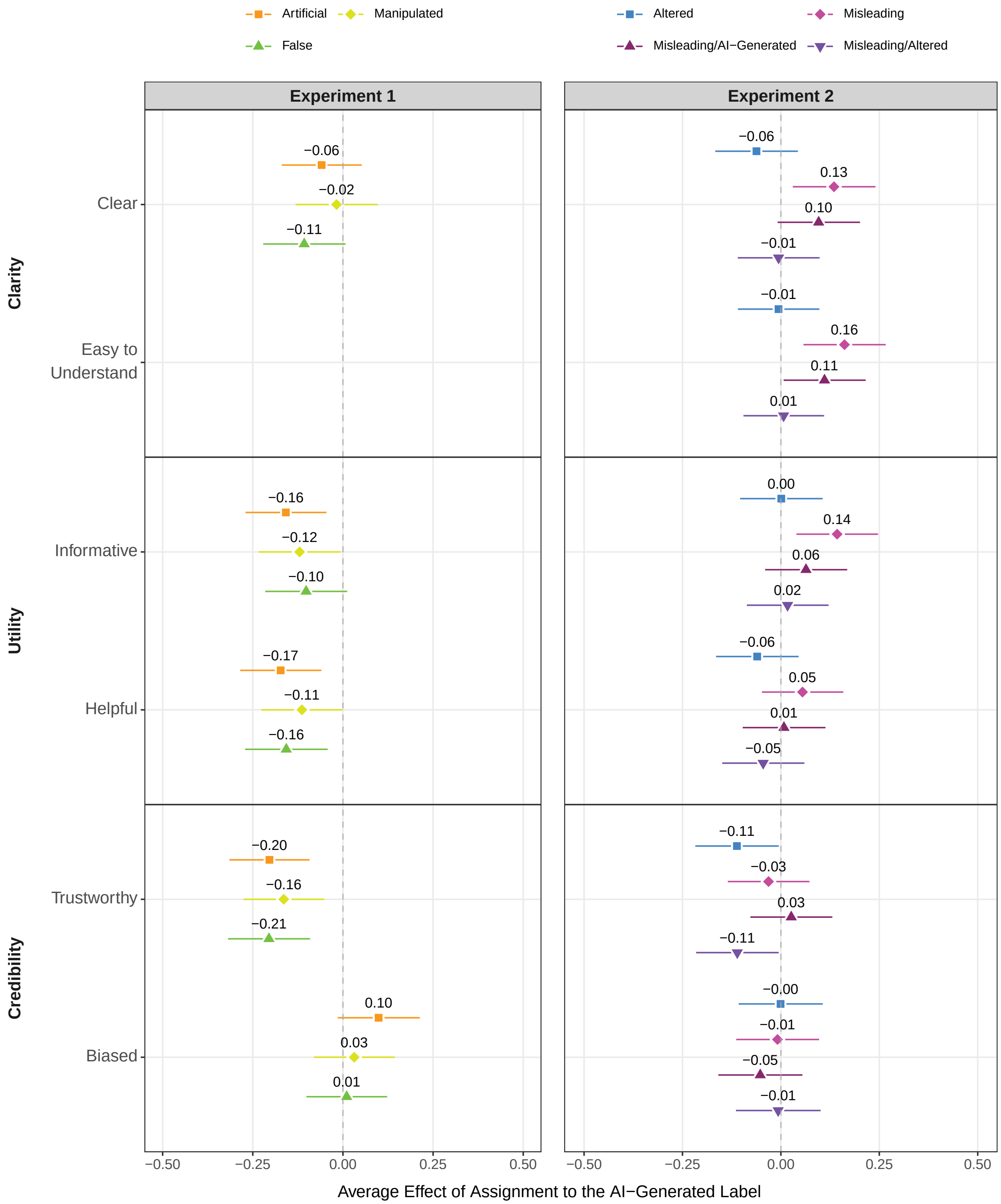

Figure S8: Average treatment effect of assignment to the **"AI-Generated"** label, relative to another label, on perceptions of label attributes. Effects are expressed in units of standard deviation and are estimated using linear regression models with stimulus fixed effects. 95% confidence intervals are based on robust standard errors. In all cases, negative values indicate that respondents were, on average, less likely to ascribe a given attribute to the "AI-Generated" label, relative to another label. Note that the measure of perceived bias is negatively valenced, such that lower ratings on this item indicate more *positive* perceptions of the AI label.

Sources of Information

Figure S9 plots the average treatment effect of assignment to the "AI-Generated" label, relative to another label, on perceptions of the label's informativeness, including the extent to which it (i) provided viewers with *new information*, (ii) helped them understand how the content was *made*, and (iii) helped them understand whether the content was *true* or *false*. In all cases, positive values indicate that the AI label was viewed as more informative than another label along that dimension, whereas negative values indicate that the AI label was seen as less informative.

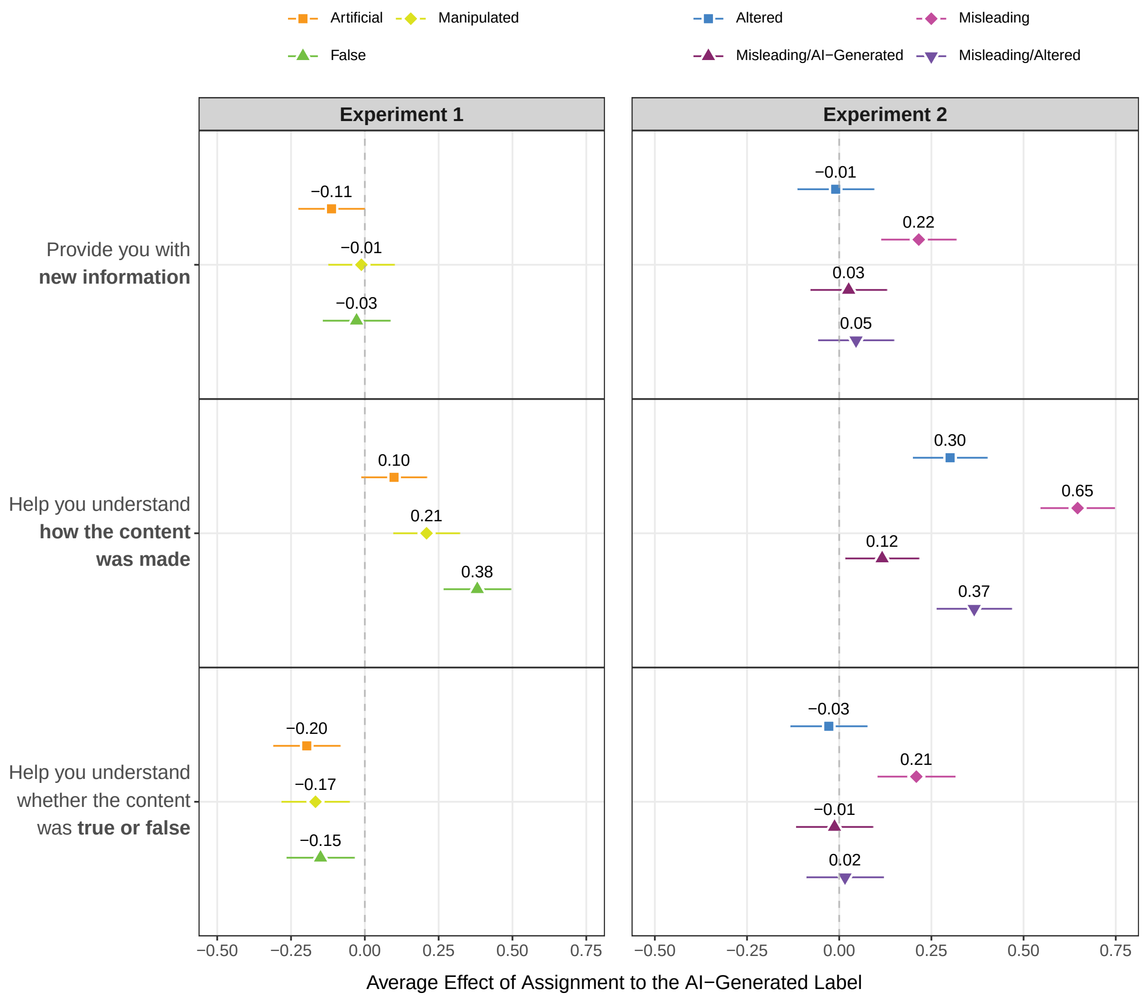

Figure S9: Average treatment effect of assignment to the **"AI-Generated"** label, relative to another label, on perceptions of informativeness. Effects are expressed in units of standard deviation and are estimated using linear regression models with stimulus fixed effects. 95% confidence intervals are based on robust standard errors. In all cases, negative values indicate that respondents were, on average, less likely to view the "AI-Generated" label as informative, relative to another label.

Comparison to "Misleading" Label

Figure S10 instead plots the average treatment effect of assignment to the "Misleading" label, relative to another condition, in Experiment 2. The left-hand panel examines different characteristics of the label, including perceptions of clarity; utility; and credibility, and the right-hand panel assesses different types of information provision. For all but the measure of bias, positive values indicate that respondents, on average, held more positive perceptions of the "Misleading" label, relative to another option, whereas negative values indicate that respondents held more negative perceptions of this label.

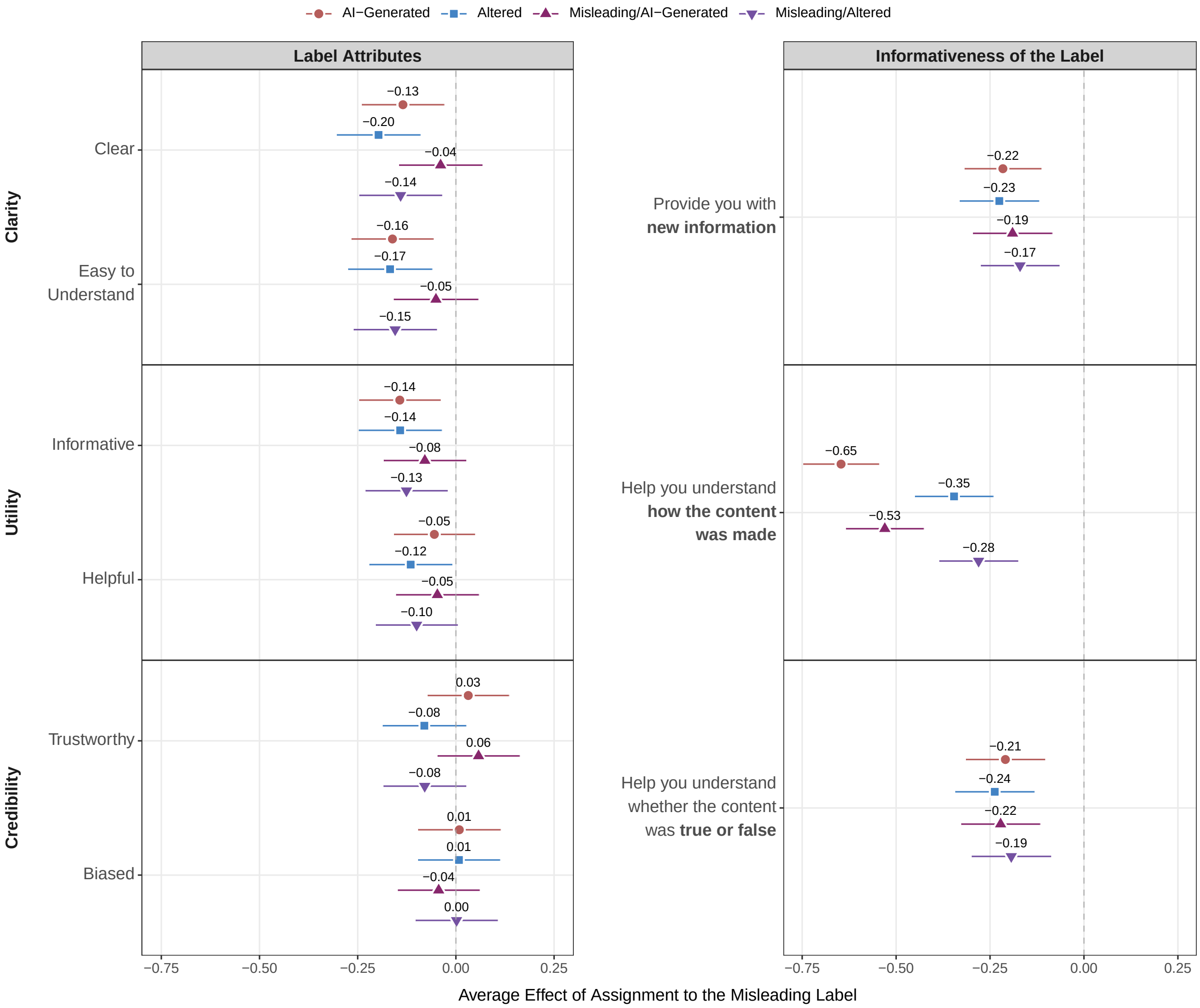

Figure S10: Average treatment effect of assignment to the **"Misleading"** label, relative to another label, in Experiment 2. The left panel plots results for perceived label attributes, whereas the right panel plots results for informativeness. Effects are expressed in units of standard deviation and are estimated using linear regression models with stimulus fixed effects. 95% confidence intervals are based on robust standard errors. In all cases, negative values indicate that respondents were, on average, less likely to ascribe a given characteristic to the "Misleading" label, relative to another label.

Overall, the results in Figure S10 suggest that the "Misleading" label in Experiment 2 may not have been sufficiently clear or informative when presented in isolation. In particular, respondents assigned to this label give significantly lower ratings, on average, for the extent to which this label provided them with new information, helped them understand how the content was made, and helped them understand whether the content was true or false. These concerns are further corroborated by respondents' open-ended comments. Though many respondents indicated that the "Misleading" label helped them better gauge the reliability of the image, a sizable number of comments expressed confusion about what, exactly, was misleading about the image. For instance, one participant wrote, "When I viewed this label I wanted to know more about why it was misleading," and another noted, "You see in big bold letters 'Misleading,' but it doesn't give you any details [about] why/what [is] misleading. The label leaves you hanging." Together, these comments highlighted several potential opportunities for improvement, including:

- **More decisive language:** First, some respondents deemed the language too *imprecise*. For example, one commenter stated, "The wording of [the label] just feels very vague to me, especially with the use of 'could,' and I think more direct language could be better to get the point across." Another likewise said that they "would have preferred it be more specific and state whether in fact the image was misleading or not vs. 'may' mislead or 'could' mislead. The warning would be more helpful by stating matter of factly if it's true or not."
  - Several respondents also mentioned that they would have preferred a "False" label (such as the one in Experiment 1), saying things like, "I think this label of 'Misleading' does not go near[ly] far enough. This post is obviously not true. The label needs to be FALSE, not Misleading, and needs to be labeled as manipulated" and "Why not just call it false or falsified?" (see also [2, 3]).
- **Additional explanation:** Some respondents asked for more *detailed explanations* about why the content was misleading, saying things like, "If you are going to tell people that something is misleading you have to explain WHY it is misleading and what about it could be misunderstood," and "The label was very vague. It did not ... specify what content was being possibly misleading to people. It was not clearly shown what the image was misleading people about. More details need to be provided" (in line with [4–6]).
  - Some respondents specifically requested the ability to click into the label to review additional resources. One respondent lamented, "I wish there was more that could be looked at. Perhaps if it was actually able to be opened, we could at least know about WHICH PART of the picture was dee[me]d misleading." Another likewise said, "It isn't clear what could be misleading" but recommended being able to "find out more by clicking."
- **Clarifying the label's source:** Finally, some respondents noted that they wanted more information about the *source* of the label (following [7–9]). For instance, one respondent wrote, "My greatest concern is WHO determines the accuracy of the content. It would be greatly helpful to know who made that determination and the motive for the posting," and another asked, "Who would post that label and why?"

- Summarizing many of the broader themes in the open-ended comments, one respondent also wrote, “The label is valueless because it doesn’t clarify who is saying the content is misleading; it doesn’t say how the content is misleading; it doesn’t say what is misleading; and it doesn’t offer any explanation for why some people could be mis[led] and others not.”

Distribution of Ratings

Finally, Figures S11 to S14 plot the distribution of label evaluations—including both perceptions of various label attributes and sources of information—disaggregated by treatment condition. Notably, while a sizable portion of respondents indicated that their assigned label was, for instance, “very” or “extremely” helpful, many also said that it was “not at all” helpful. These patterns may reflect differing levels of familiarity with certain technologies, as well as differing abilities to discern synthetic media absent labeling. Indeed, in their open-ended comments, a number of respondents in both studies indicated that they had clocked the image’s manipulation even before they read the label. From this perspective, a label may not be as informative for those who are already aware of the risks of AI and mindful of the content they engage with—but this does not necessarily imply that labels lack utility across the board.

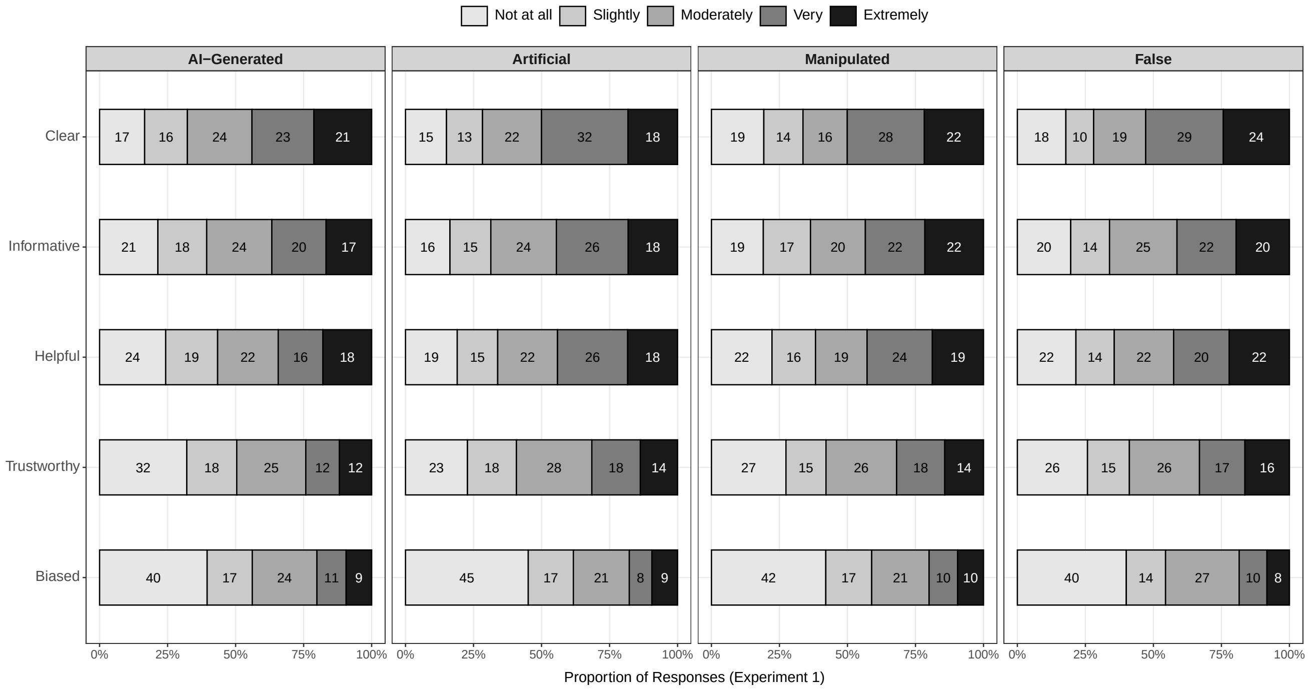

Figure S11: Distribution of ratings in Experiment 1 for label *attributes*, disaggregated by labeling condition. In all cases, the scale ranged from 1-5, where 1 = “Not at all” and 5 = “Extremely”; for the measure of bias, this indicates that higher ratings are associated with *more* perceived bias. Note that these items were not asked of respondents in the unlabeled control group.

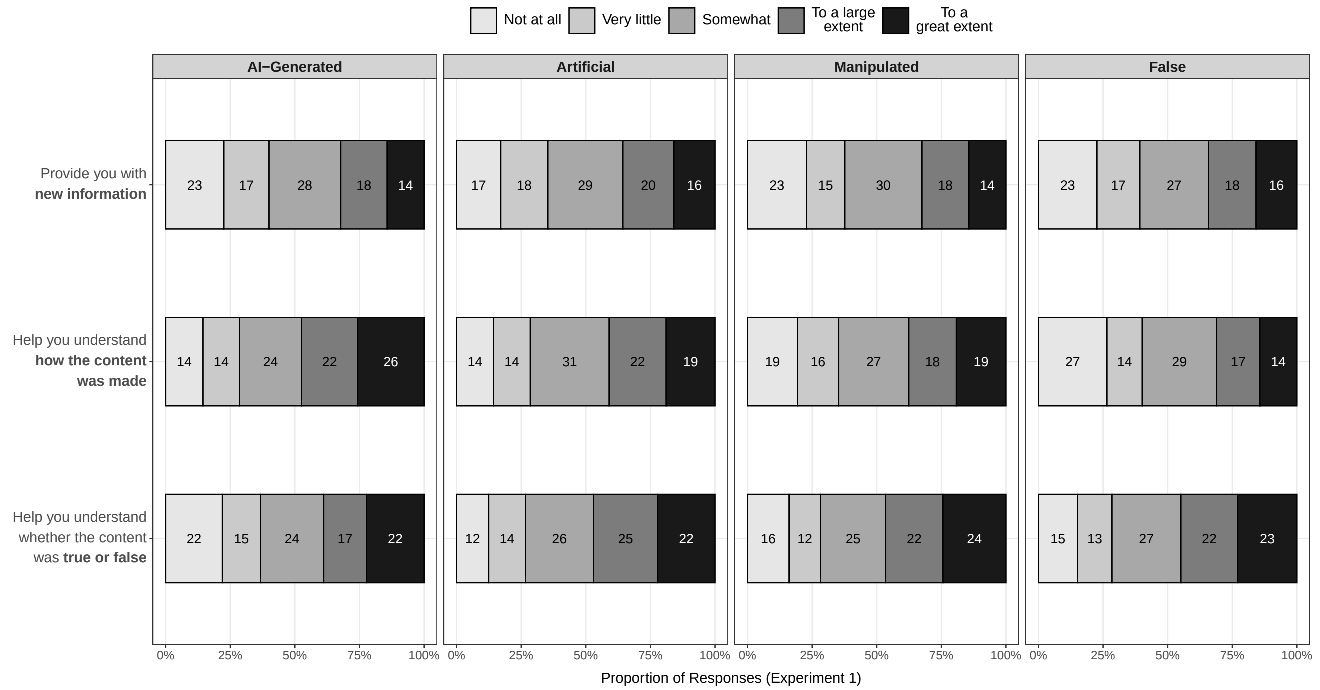

Figure S12: Distribution of ratings in Experiment 1 for sources of *information*, disaggregated by labeling condition. In all cases, the scale ranged from 1-5, where 1 = "Not at all" and 5 = "To a great extent," where higher ratings indicate that the label was perceived as more informative. Note that these items were not asked of respondents in the unlabeled control group.

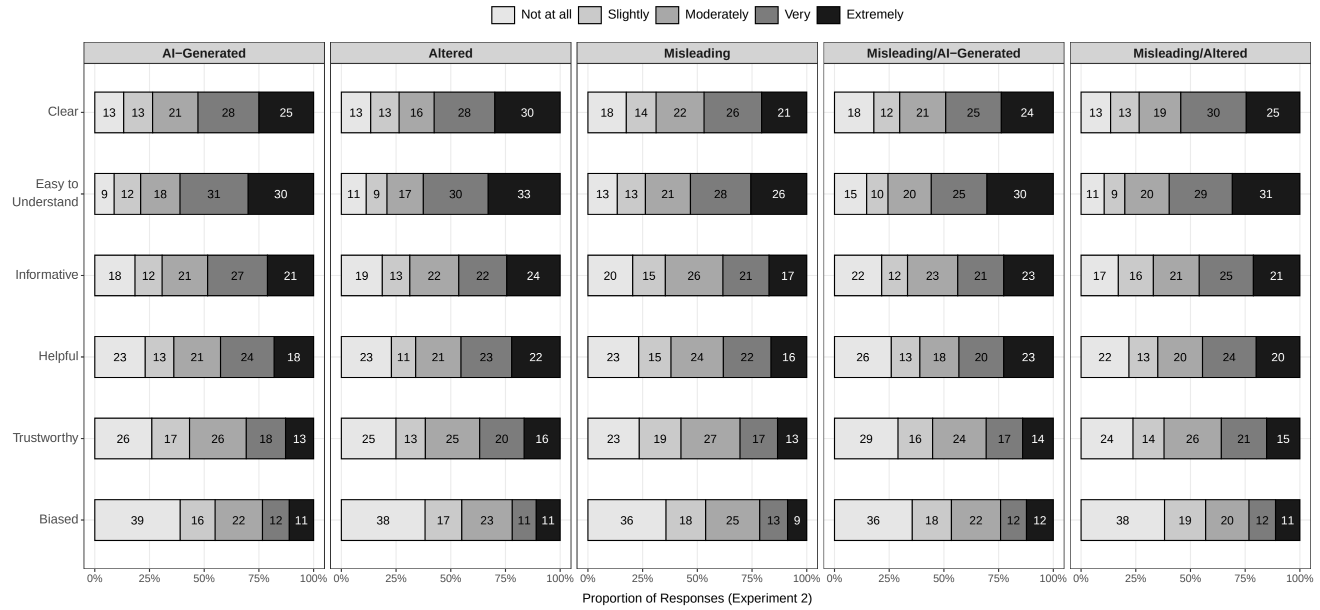

Figure S13: Distribution of ratings in Experiment 2 for label *attributes*, disaggregated by labeling condition. In all cases, the scale ranged from 1-5, where 1 = "Not at all" and 5 = "Extremely"; for the measure of bias, this indicates that higher ratings are associated with *more* perceived bias. Note that these items were not asked of respondents in the unlabeled control group.

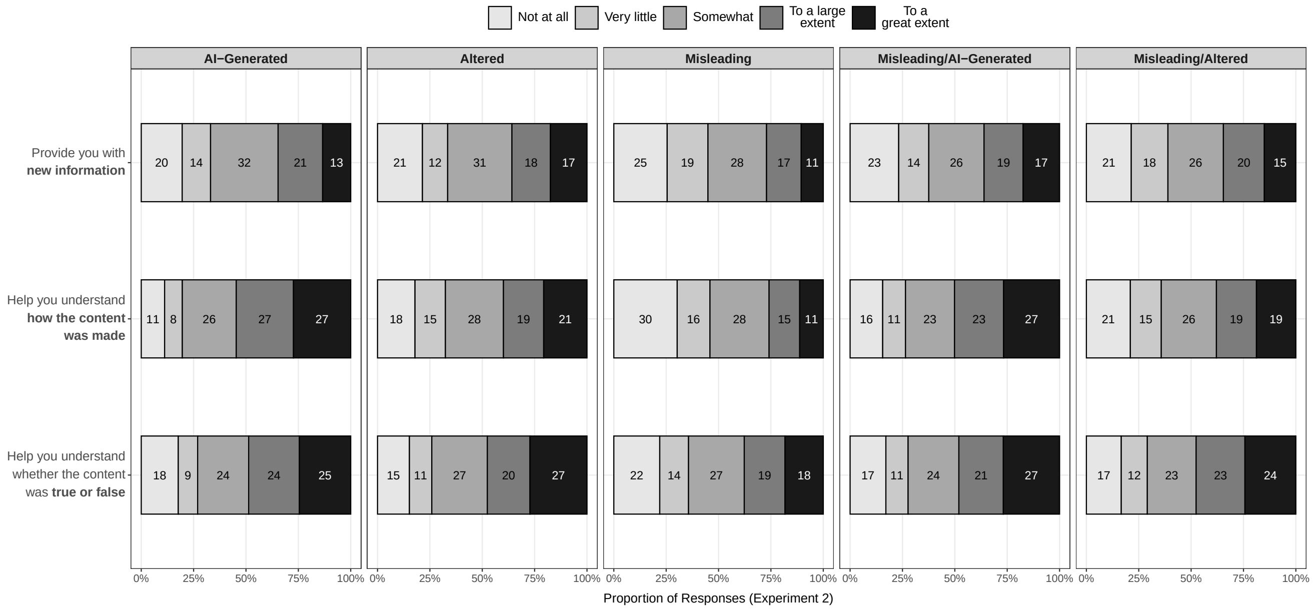

Figure S14: Distribution of ratings in Experiment 2 for sources of *information*, disaggregated by labeling condition. In all cases, the scale ranged from 1-5, where 1 = "Not at all" and 5 = "To a great extent," where higher ratings indicate that the label was perceived as more informative. Note that these items were not asked of respondents in the unlabeled control group.

3 Treatment Effect Heterogeneity

3.1 External Ratings Task

As a supplement to our main experiments, we also collected data in December 2023 about the posts in our stimulus set (n = 525; 1,546 total observations). After providing informed consent and completing the same attention checks as our experiments, participants in this study reviewed a detailed set of instructions describing each component of the ratings task. Specifically, participants were informed that they would be asked to review three social media posts and answer four sets of questions about each post, including:

1. Rating several characteristics of the **image** shown in the post (e.g., how interesting or surprising it is).
2. Evaluating whether the information in the post is **important** for people to know.
3. Assessing how **believable** the post is, as well as whether they've **seen** or **heard anything** about it before.
4. Indicating whether the posts pertains to **politics** and, if so, whether its content is more favorable to **Democrats** or **Republicans**.

As part of this process, participants were required to complete a comprehension check to confirm their understanding of the instructions, though they were not prohibited from completing the study if they failed this task. Overall, 73% of participants correctly answered the comprehension check on the first try. Participants who provided an incorrect answer were asked to review the instructions again; 29% of these participants passed the comprehension check on their second try. In total, 81% of participants passed the comprehension check across these two attempts.

Image Attributes

After finishing the comprehension check, participants were then asked to review three posts, randomly selected from the 29 stimuli included in our experiments. Each post was assigned to approximately 50 raters (range: 52-55). Because the goal of the ratings task was to assess baseline characteristics of the posts, absent labeling, participants always viewed the *unlabeled* version of the post, equivalent to what the control group saw in the experiments. Participants were first asked to rate eleven attributes of the image, designed to assess perceived *credibility*, *interestingness*, and *informativeness* (where starred items were reverse-coded):

- "To what extent is the image in this post...?"
  (1 = Not at all, 2 = Slightly, 3 = Moderately, 4 = Very, 5 = Extremely; order of attributes randomized within and across two screens)
  - **Credibility:** Believable, Authentic, Accurate, Deceptive*, Fake*
  - **Interestingness:** Surprising, Creative, Interesting, Boring*, Amusing
  - **Informativeness:** Informative

Societal Importance

Participants were then asked about the extent to which the information in the post was *important* for people to know. Note that this item was designed to assess perceptions of societal versus personal importance.

- "To what extent is the information shown in this post important for people to know?"
  (1 = Not at all important, 2 = Slightly important, 3 = Moderately important, 4 = Very important, 5 = Extremely important)

Baseline Beliefs

To measure baseline *belief* in and *familiarity* with the post, we then asked participants to indicate whether they thought the events shown in the post really took place and whether they had previously seen or heard anything about the post. Overall, relatively few people indicated prior familiarity with the posts; indeed, the median post was familiar to only 12% of participants. Higher levels of familiarity tended to be associated with posts containing misinformation about real-world events (e.g., Trump's arrest, protests in Paris, or King Charles's coronation). Thus, our estimates of familiarity are, if anything, likely to be inflated, as participants may have been reporting whether they knew about the broader event in question (as opposed to indicating that they had seen the exact image or claim before). However, even in the most extreme cases, less than one-third of respondents reported having seen or heard anything about the post (maximum of 29%), suggesting the posts were likely not recognizable to many experimental respondents, even though they had circulated online in recent months.

- **Believability:** "To the best of your knowledge, did the events shown in this post actually happen?"
  (1 = Definitely did not happen, 2 = Probably did not happen, 3 = Not sure if did or did not happen, 4 = Probably happened, 5 = Definitely happened; order of response options randomly flipped)
- **Familiarity:** "Have you seen or heard anything about this post before?"
  (1 = Yes, 0 = No, 88 = Not sure)

Political Slant

Finally, participants indicated whether they thought the posts were *political* or not. If they identified the post as political, they were then asked to gauge the *slant* of the post—namely, whether it was more favorable to Democrats or Republicans (or favored neither party).

- **Political:** "In your opinion, is this post **political** or **non-political**?"
  (1 = Political, 0 = Non-political, 88 = Not sure)
- **Slant:** *(If participants selected "Political" above)* "In your view, is the content of this post more favorable to **Democrats** or **Republicans**?

  Please select one option below. If you think the post relates to American politics but does

not favor either Democrats or Republicans, please select 'Not favorable to one party over the other.' If you think the post is unrelated to American politics, however, please select 'Not applicable.'"

*(1 = More favorable to **Democrats**, 2 = More favorable to **Republicans**, 3 = Not favorable to **one party over the other**, 77 = Not applicable, 88 = Not sure)*

– Given the complexity of this construct, the comprehension check at the beginning of the survey related to this item.

– We also included a button that, if clicked, allowed participants to review several examples while answering the relevant prompts.

  • "A post may be favorable toward Republicans if it supports a position commonly associated with the Republican Party or depicts Republicans in a positive light."
  • "In addition, a post may be favorable to Republicans if it opposes a position commonly associated with the Democratic Party or depicts Democrats in a negative light."
  • "Finally, it is possible that a post may not favor one party over another or may not be related to American politics."

As summarized in Table S9, we classified posts as political if a plurality of participants reported that they were political, as opposed to indicating they were non-political or saying they were unsure. Notably, several posts about the personal lives of political figures (e.g., `musk_aoc` and `obama_pregnancy`) were largely deemed non-political, despite featuring partisan politicians. In addition, participants often disagreed about the slant of the presented content. For instance, for the `biden_buddhism` post, which alleged that Joe Biden had recently converted to Buddhism, 40.5% of participants who indicated the post was political said it was more favorable to Democrats, whereas 37.8% said it was more favorable to Republicans. This lack of consensus likely reflects some ambiguity about the tone and veracity of the posts; namely, people might perceive a post as favorable to Democrats if they believe it is sincere but favorable to Republicans if they view it as satire or commentary.

Given the limited number of political posts and the level of disagreement regarding their slant, we therefore opted not to assess heterogeneity in labeling effects based on their political congruence (in a departure from a pre-registered exploratory analysis). Future work should investigate this topic further using a wider variety of political posts whose slant is less ambiguous and/or whose slant is directly manipulated as part of the experimental design.

Image ID	Coded as Political?	Political	Non-Pol.	Unsure	# Slant Ratings	Favors Dems	Favors Reps	No Slant/ Unsure
beached_shark		9.3	85.2	5.6	5	-	-	-
biden_bubble	✓	66.7	21.6	11.8	34	17.6	58.8	23.5
biden_buddhism	✓	69.8	13.2	17.0	37	40.5	37.8	21.6
biden_celebrate	✓	69.8	15.1	15.1	37	59.5	21.6	18.9
booker_dress	✓	49.1	36.4	14.5	27	22.2	63.0	14.8
cruise_doubles		5.8	88.5	5.8	3	-	-	-
dalai_arrest	✓	49.0	26.5	24.5	24	25.0	12.5	62.5
hybrid_turtle		5.7	88.7	5.7	3	-	-	-

Image ID	Coded as Political?	Political	Non-Pol.	Unsure	# Slant Ratings	Favors Dems	Favors Reps	No Slant/ Unsure
johnson_arrest	✓	63.0	13.0	24.1	34	14.7	23.5	61.8
lobster_caught		7.5	86.8	5.7	4	-	-	-
melania_arrest	✓	58.5	22.6	18.9	31	51.6	25.8	22.6
moon_landing		28.3	58.5	13.2	15	-	-	-
musk_aoc		22.6	60.4	17.0	12	-	-	-
musk_barra		14.8	72.2	13.0	8	-	-	-
musk_robot		11.5	78.8	9.6	6	-	-	-
obama_pregnancy		32.1	50.9	17.0	17	35.3	17.6	47.1
paris_trash	✓	64.0	20.0	16.0	32	15.6	25.0	59.4
paul_dress	✓	66.7	20.4	13.0	36	11.1	36.1	52.8
pence_pride	✓	70.9	18.2	10.9	39	30.8	25.6	43.6
putin_arrest	✓	66.0	22.0	12.0	33	24.2	15.2	60.6
putin_collapse	✓	55.8	26.9	17.3	29	17.2	13.8	69.0
royal_reunion		34.0	52.8	13.2	18	50.0	11.1	38.9
satan_pope		32.7	48.1	19.2	17	35.3	5.9	58.8
satan_school	✓	46.3	42.6	11.1	25	32.0	36.0	32.0
satan_target		31.4	58.8	9.8	16	18.8	37.5	43.8
trump_arrest	✓	82.7	13.5	3.8	43	53.5	25.6	20.9
trump_crying	✓	76.4	10.9	12.7	42	59.5	16.7	23.8
trump_fauci	✓	61.5	21.2	17.3	32	21.9	43.8	34.4
walmart_tipping		25.5	50.9	23.6	14	-	-	-

Table S9: Classification of the political slant of posts in the ratings task. Posts are categorized as "political" when a plurality of participants selected this option (as opposed to "non-political" or "not sure"). If participants indicated that a post was political, they were then asked whether its content was more favorable to the Democratic Party, Republican Party, or neither party; they also had the option of indicating "Not applicable" for posts not about American politics (e.g., posts about international affairs) or "unsure" when they were uncertain about the slant. We only report the distribution of slant ratings for posts that received more than 15 responses to this measure.

3.2 Post-Level Moderators

To explore potential moderators of labeling effects, we first estimate a series of linear regression models that interact an indicator of assignment to a labeled versus unlabeled post (designated as `label` below) with a vector of respondent- and post-level characteristics. For these models, we exclude the stimulus fixed effects used in our main analyses, as the post-level characteristics do not vary within stimuli. We input all of these variables simultaneously in order to mitigate potential confounding; for instance, political images tend to be seen as more socially important but less credible and original than non-political images. However, the results are substantively similar if we instead estimate separate models for each moderator variable.

```
library(estimatr)

# Example moderator model
mod <- lm_robust(dv ~ label * (age + diglit + party + survey_attent + share +
                              belief_post + int_post + import_post + pol_post),
                 data = df)
```

In total, we examine four sets of post-level covariates, all designated using the `*_post` suffix. First, to create the `belief_post` variable, we subset our data to the control group and take an average of responses to the belief outcome (i.e., measure respondents' belief in the core claims implied by the post's image, in the absence of labeling). For the remaining variables, we use responses from the rating task to construct our estimates. For the `pol_post` variable, we categorize each post as political if a plurality of participants in the rating task indicated it was political (as opposed to reporting that it was either non-political or that they were unsure). Note that there is some uncertainty in these estimates that is not currently propagated through to the linear regression models; as such, the results below should be interpreted as suggestive and exploratory.

- `belief_post`: the baseline *believability* of the post, absent labeling; based on average ratings of respondents in the control group of each study, where higher scores indicate that the post's claims were more believable.

- `int_post`: how *interesting* the image was; measured as an average of five attributes collected during the rating task: the extent to which the (unlabeled) image was (i) surprising, (ii) creative, (iii) interesting, (iv) amusing, and (v) boring *(reverse-coded)*. Higher ratings on this measure indicate that the post's image was seen as more novel and entertaining.

- `import_post`: the post's societal *importance*, based on average ratings in the rating task, where higher ratings indicate that the information in the post was seen as more important for people to know.

- `pol_post`: whether the post was classified as *political* in the ratings task.

Figure S15 summarizes the estimated coefficients from the resulting models. Note that, although these models include both respondent- and post-level covariates, we solely display the results for the post-level variables here; results for the respondent-level covariates can be

found below in Section 3.3. Positive (negative) values for the base terms indicate that the post-level variable is positively (negatively) correlated with beliefs/sharing intentions in the control group. In addition, given that the overall effects of labeling are negative for both beliefs and sharing intentions, positive (negative) values for the interaction terms indicate that the effects of labeling are expected to be weaker (stronger) as that attribute increases. The scores for believability, importance, and interestingness are standardized, and the dummy variable for political versus non-political posts is center-coded, such that the mean in all cases is equal to zero.

(a) Experiment 1

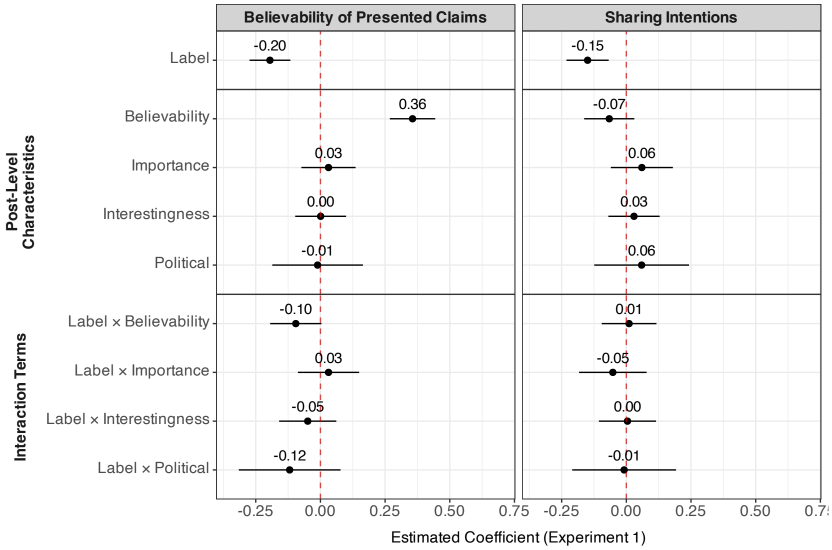

(b) Experiment 2

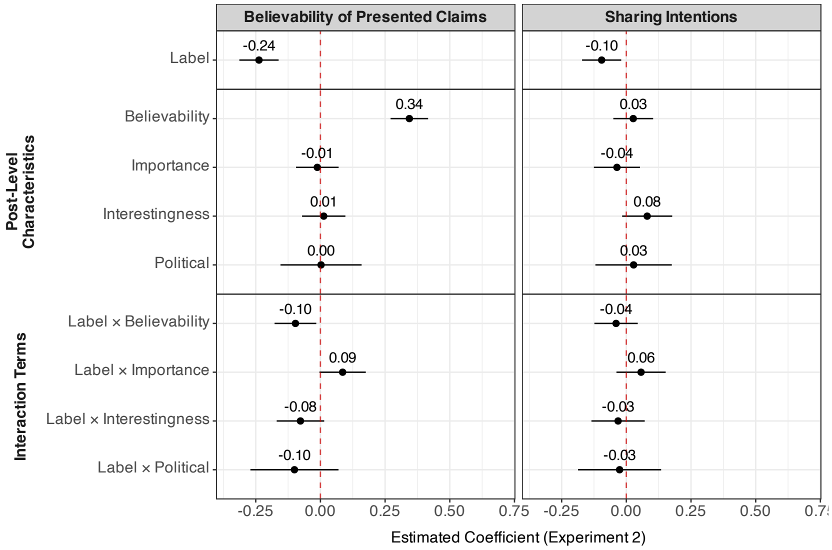

Figure S15: Estimated coefficients from models linearly interacting an indicator of assignment to a labeled versus unlabeled post with each of the individual- and post-level covariates (entered simultaneously) in each experiment. For readability, we display the results for the post-level variables here; results for respondent-level variables can be found in Figure S22.

Differences by Baseline Beliefs

As shown in Figure S15, we find in both studies that labeling has a marginally larger effect on beliefs (i.e., more strongly reduces people's belief in the presented content) for posts that are, all else equal, more plausible absent labeling. To further illustrate this point, Figure S16 visualizes the estimated effect of viewing a labeled versus unlabeled version of a post across different levels of baseline believability, using the `marginaleffects` package in `R` [10]. In both studies, labeling has a negligible effect on viewers' beliefs for the most implausible stimuli—likely because people did not need a label to recognize the claims as suspect. By contrast, labeling has a much larger, negative effect on beliefs in cases where respondents, on average, thought the presented events actually took place, suggesting that labeling may be a more valuable tool when posts would otherwise be convincing.

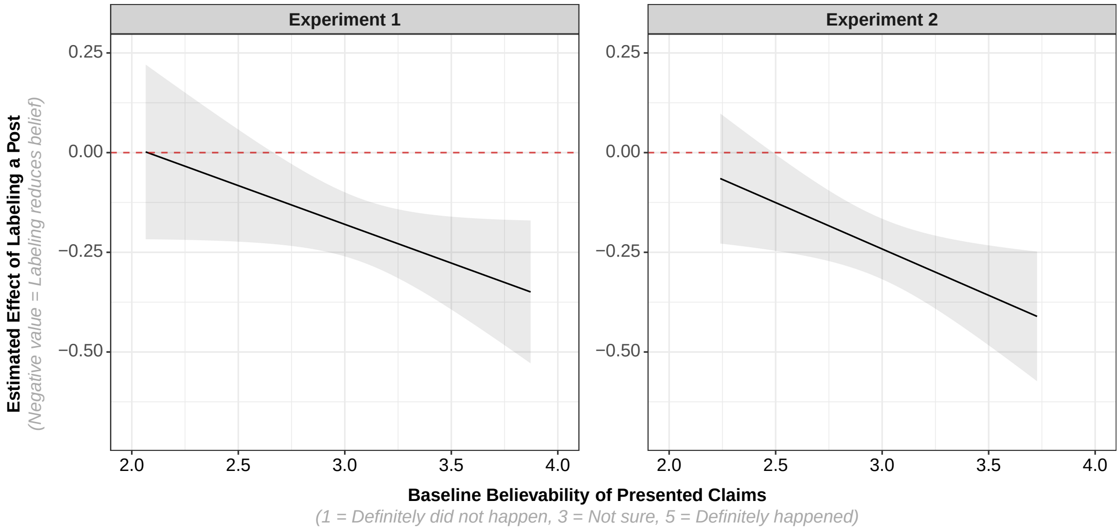

Figure S16: Differences in the estimated effect of labeling on belief in the post's claims, based on the baseline believability of the presented content. Believability is measured using ratings from respondents in the *control group* of each study; scores range from 1-5, where lower ratings indicate less belief in the claims associated with the AI-generated image. Estimates and 95% confidence intervals are calculated using the `marginaleffects` package in `R`.

In Figure S17, we repeat this analysis, this time using external evaluations of each post derived from the rating task summarized in Section 3.1. For Experiment 1, we find a similar pattern of results: labeling is predicted to have a significantly larger effect on beliefs for posts whose content appears more credible at baseline. However, for Experiment 2, this relationship no longer emerges. One possible explanation for this deviation is that the survey sample for Experiment 2 may have differed from the sample for the ratings task (and Experiment 1; see Table S1 in Section 1.1), resulting in divergent interpretations of the believability of the presented stimuli.

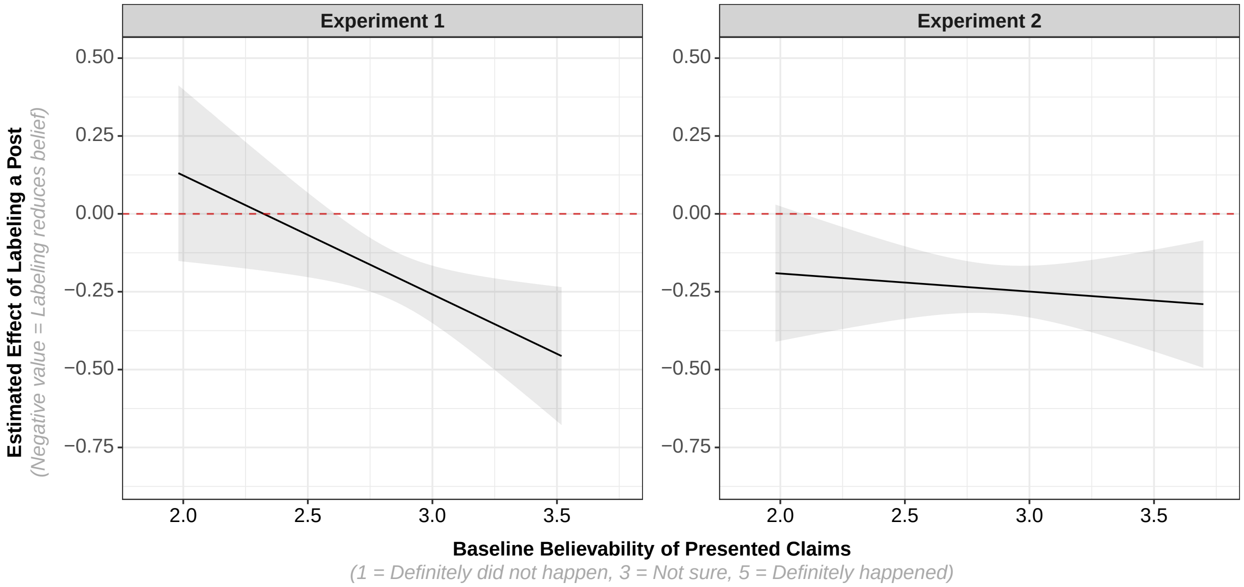

Figure S17: Differences in the estimated effect of labeling on belief in the post's claims, based on the baseline believability of the presented content. Believability is measured using responses to the external *ratings task* summarized in SI Appendix, Section 3.1. Estimates and 95% confidence intervals are calculated using the `marginaleffects` package in `R`.

Figures S18 and S19 break up the overall results in Figure S16 by the individual labels (for Experiments 1 and 2, respectively). With the exception of the "Artificial" label in Experiment 1, the results for each of the labels mirror the broader findings, in that labeling is expected to have a larger effect as baseline believability increases. However, we have relatively little data with which to detect heterogeneity at this level of granularity; future work should seek to replicate these patterns using larger samples of respondents, images, and/or labels.

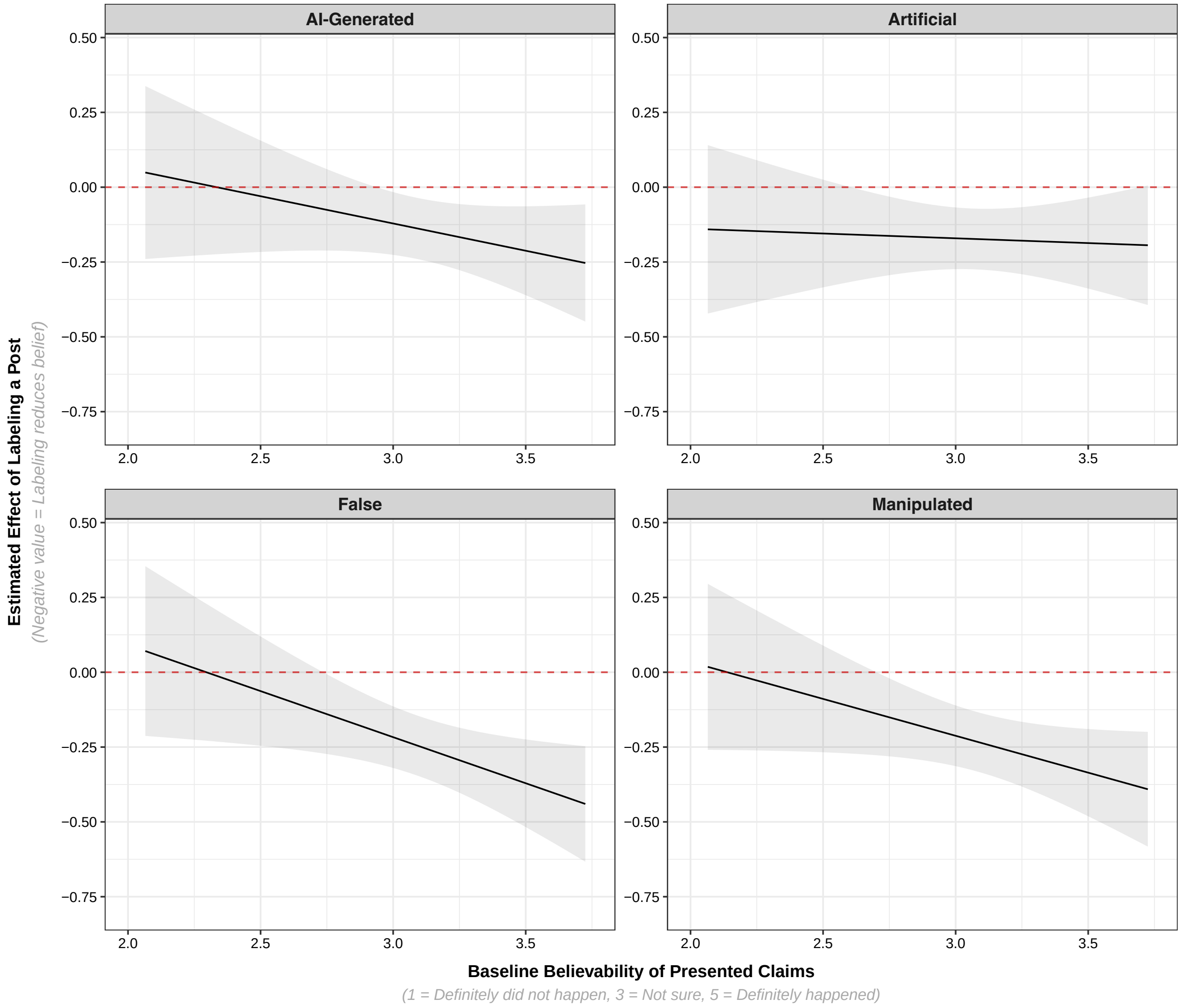

Figure S18: Differences in the estimated effect of different labels on belief in the post's claims, based on the baseline believability of the presented content in *Experiment 1*. Believability is measured using ratings from respondents in the *control group* of each study. Estimates and 95% confidence intervals are calculated using the `marginaleffects` package in `R`.

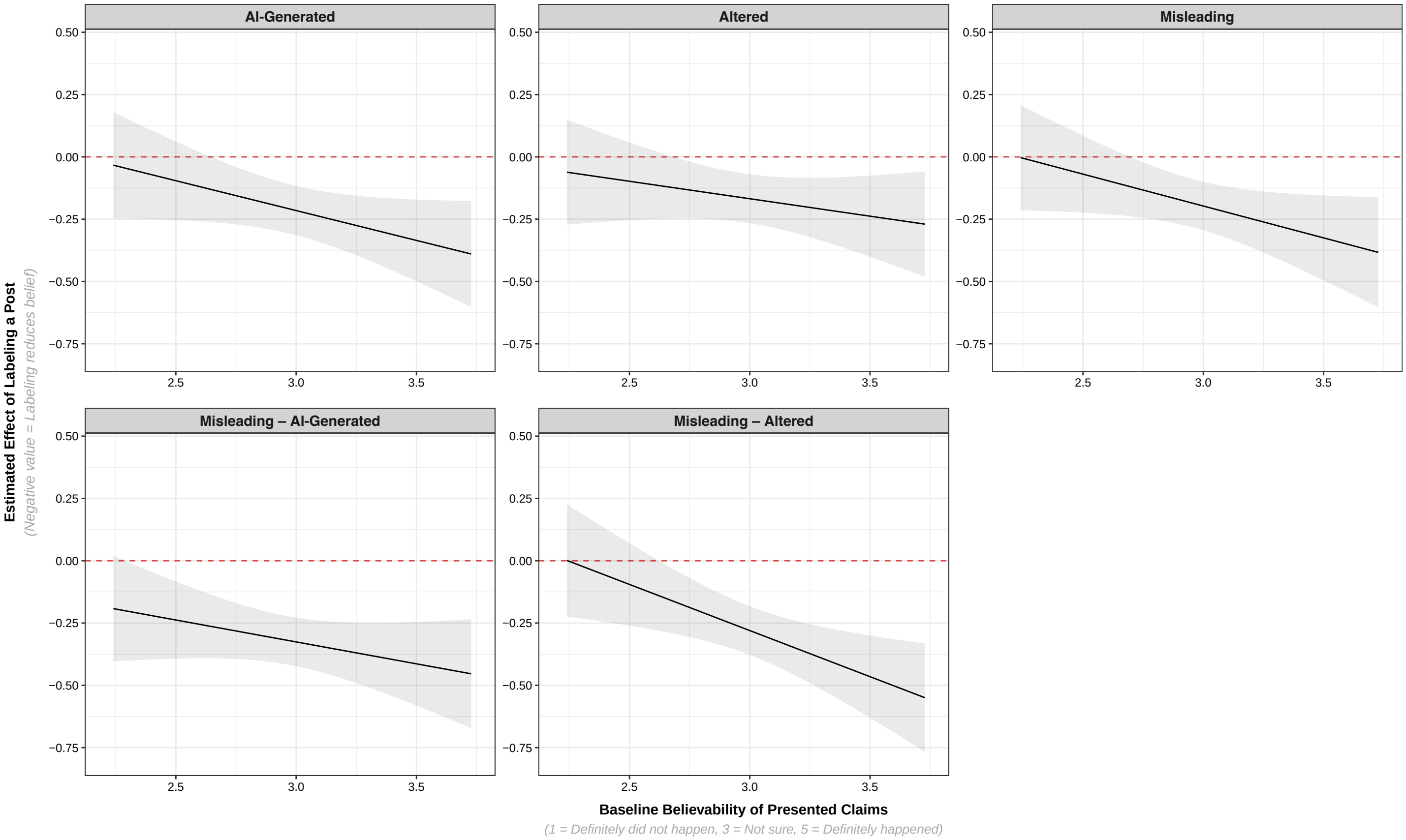

Figure S19: Differences in the estimated effect of different labels on belief in the post's claims, based on the baseline believability of the presented content in *Experiment 2*. Baseline believability is measured using ratings from respondents in the *control group* of each study. Estimates and 95% confidence intervals are calculated using the `marginaleffects` package in `R`.

Differences between Political and Non-Political Stimuli

Much of the prevailing discourse around AI-generated misinformation focuses on the potential for this technology to be used in the political domain. As such, it is important to understand whether the effects of labeling diverge for political versus non-political stimuli. Figure S20 plots the marginal effects of labeling for these two categories of content, controlling for the other individual- and post-level covariates described in the model specification above, in order to account for variability in the baseline believability, interestingness, and societal importance of political vs. non-political content. In Figure S21, we also replicate these results without incorporating other control variables.

Overall, across both sets of analyses, we find minimal evidence that the effects of labeling are weaker for the political versus non-political posts in our stimulus set. On the contrary, the effects, if anything, trend slightly *larger* in some cases for political versus non-political stimuli, though the observed differences are relatively small and not statistically significant. For both types of posts, exposure to labeling (of any kind) significantly reduces belief in AI-generated misinformation. Likewise, the impact of labeling on sharing intentions is directionally negative in all cases, though the effects are not always discernible from zero across studies. However, it is important to note that we examined a small number of stimuli here; in Experiment 1, 9 out of the 14 posts were classified as political, along with 16 of the 29 posts in Experiment 2.

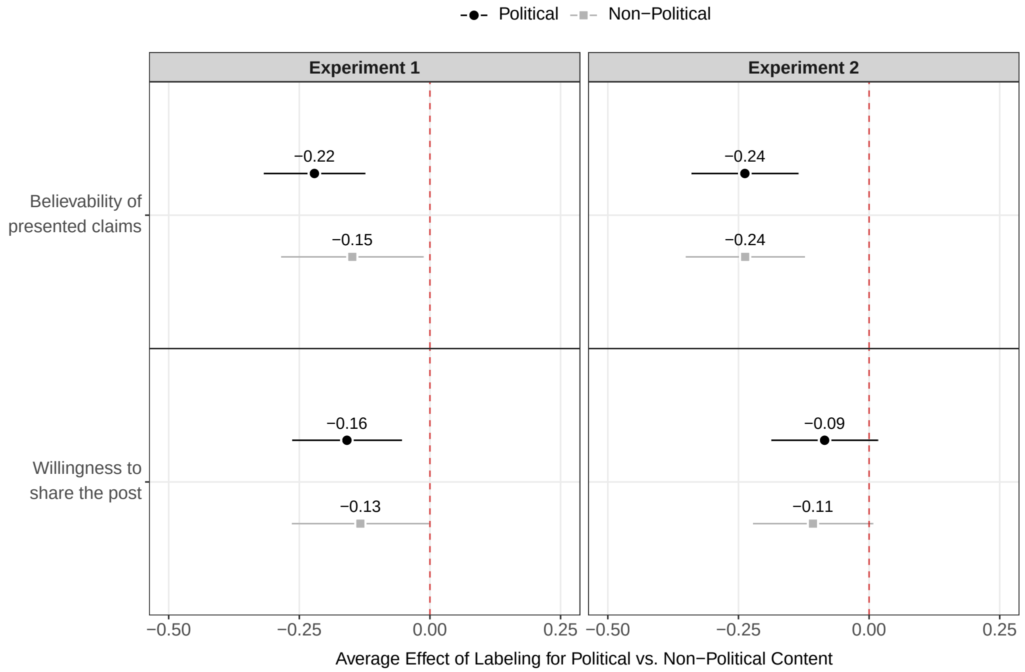

Figure S20: Estimated effect of labeling on beliefs and sharing intentions for political versus non-political posts. Posts are coded as political or non-political based on responses to the external ratings task (SI Appendix, Section 3.1). Estimated effects come from the *full model* (controlling for other individual- and post-level covariates) and are calculated using the `marginaleffects` package in `R`. 95% confidence intervals are displayed.

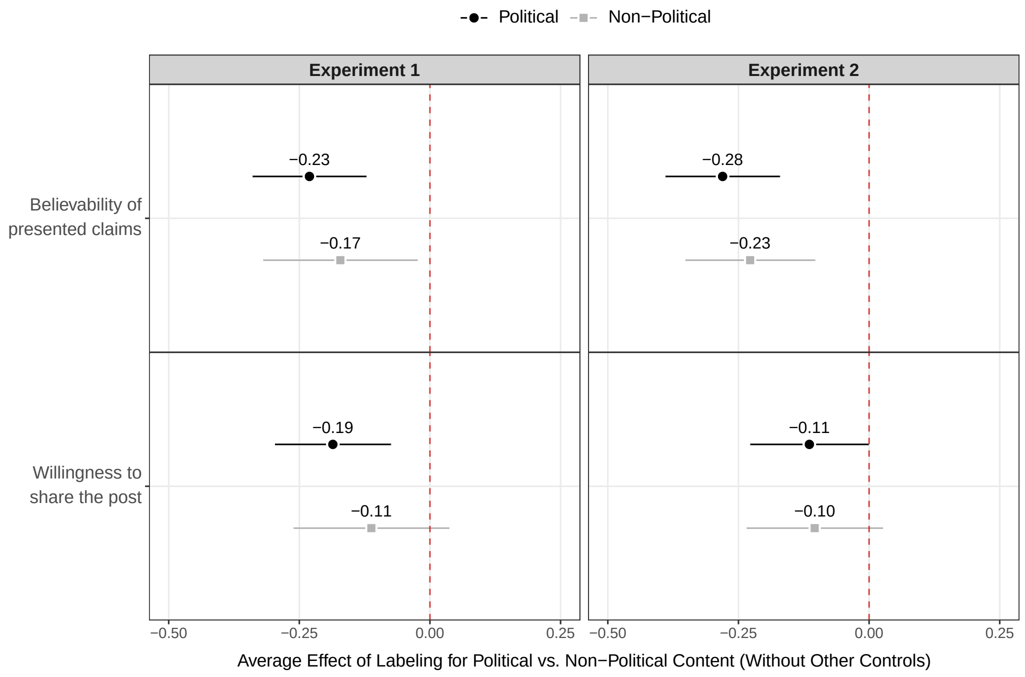

Figure S21: Estimated effect of labeling on beliefs and sharing intentions for political versus non-political posts. Posts are coded as political or non-political based on responses to the external ratings task (SI Appendix, Section 3.1). Estimated effects come from a linear regression model interacting the treatment indicator with an indicator of whether the post was political (*omitting* other individual- and post-level covariates). 95% confidence intervals are displayed.

3.3 Individual-Level Moderators

As part of the exploratory analyses specified in the pre-analysis plan, we also examined several respondent-level attributes that could potentially moderate the impact of labels on viewers' beliefs and behavior. All of these variables were collected pre-treatment. For our main model specifications, we treat each of our variables as continuous, with the exception of social media sharing, which we dichotomize. However, to probe potential non-linearities in the relationship between the moderator and treatment variables [11], we also bin several variables into tertiles and estimate treatment effects separately within each tertile.

- `age`: respondent age (in years), collected by Lucid prior to the survey.
- `diglit`: digital literacy, measured as the proportion of correct responses to five factual questions assessing knowledge about computers, technology, and social media.
- `party`: party identification, measured as a seven-point scale, where 1 = strong Democrat, 4 = Independent, and 7 = strong Republican.
- `survey_attent`: the proportion of correct responses to two instructional manipulation checks [12] embedded among the pre-treatment covariates.
- `share`: whether respondents said they would consider sharing any news on social media (e.g., about politics, sports, celebrities, or other topics).
  - Note that approximately two-thirds of respondents indicated that they would be willing to share at least one type of information on social media and are therefore treated as "sharers" for these exploratory analyses; however, future work should seek to measure sharing behavior in a more granular way—ideally using actual vs. self-reported measures of prior social media engagement.

Figure S22 summarizes the estimated coefficients on both the base and interaction terms, based on the model specification described in the prior section. In all cases, positive (negative) values for the base terms indicate that a given variable is positively (negatively) correlated with beliefs/sharing intentions in the control group. In contrast, because the overall effects of labeling are negative for both beliefs and sharing intentions, positive (negative) values for the interaction terms indicate that the effects of labeling are predicted to be smaller (larger) as that attribute increases (e.g., among older or more Republican respondents). The measure of social media use (`share`) is split into sharers versus non-sharers and is center-coded (i.e., de-meaned). The remaining variables are standardized, such that the mean of each variable is set to zero.

When examining the associations between individual characteristics and beliefs/sharing intentions in the control group, we find in both experiments that older and more digitally literate respondents, all else equal, tend to express less belief in and likelihood of sharing posts containing AI-generated images. In addition, respondents who regularly share content on social media are, in fact, substantially more likely to say they would share the presented content and are slightly, albeit not significantly, more likely to express belief in the post's main claims. However, across studies we find inconsistent evidence that these individual-level characteristics moderate the effects of labeling on viewers' beliefs and engagement intentions. Below, we explore each of these comparisons in more detail.

(a) Experiment 1

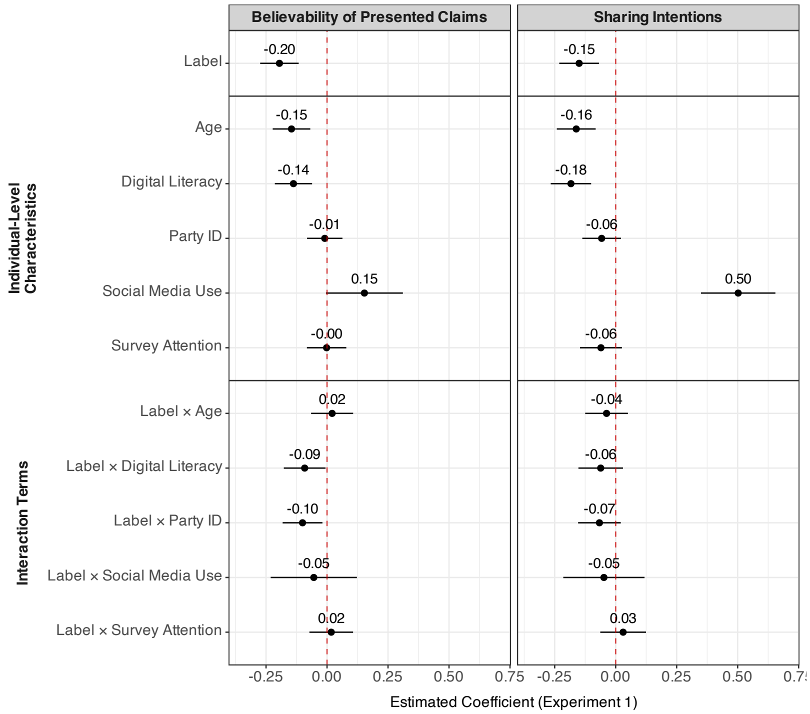

(b) Experiment 2

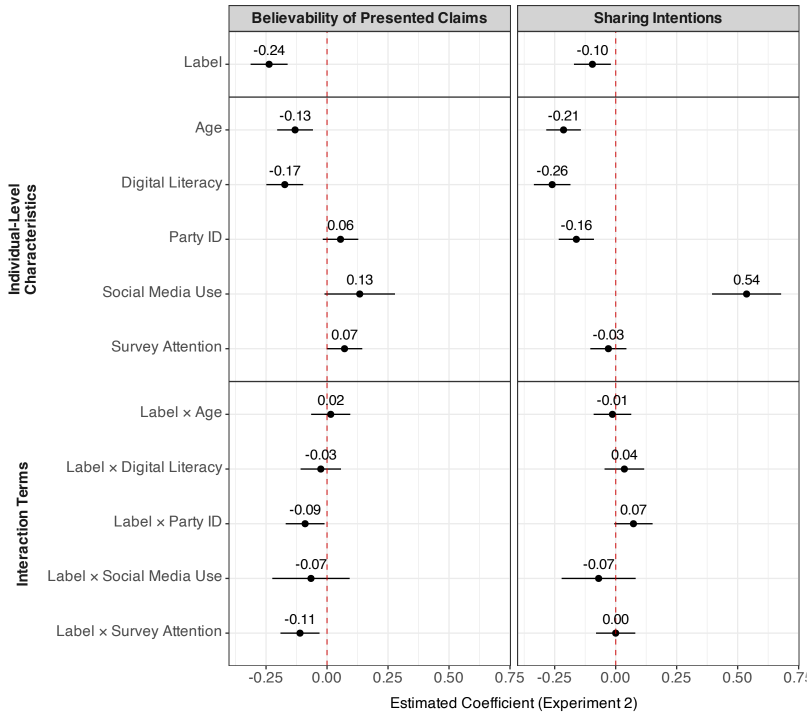

Figure S22: Estimated coefficients from models linearly interacting an indicator of assignment to a labeled versus unlabeled post with each of the individual- and post-level covariates (entered simultaneously) in each experiment. For readability, we display the results for the respondent-level variables here; results for the post-level variables can be found in Figure S15.

Tables S10 through S13 break apart the results from the combined model in Figure S22 into separate models for each moderator variable. We input social media sharing as a dummy variable taking on a value of 1 if a respondent indicated that they ever share news on social media, 0 otherwise; the base term on the treatment indicator therefore corresponds to the estimated effect of labeling among non-sharers. We standardize the remaining variables, such that they have a mean 0 and standard deviation of 1. In all cases, we include stimulus fixed effects and report heteroskedastic-robust standard errors ("HC2" variant).

	**Age**	**Digital Literacy**	**Party**	**Attentiveness**	**Sharing**
Label	−0.204*	−0.199*	−0.209*	−0.206*	−0.165*
	(0.041)	(0.041)	(0.042)	(0.042)	(0.070)
Moderator	−0.148*	−0.125*	−0.022	−0.086*	0.253*
	(0.036)	(0.037)	(0.039)	(0.039)	(0.078)
Label × Moderator	0.011	−0.097*	−0.117*	0.002	−0.057
	(0.041)	(0.042)	(0.043)	(0.044)	(0.087)
Num. Obs.	3,080	3,080	3,080	3,080	3,080
R^2	0.127	0.149	0.123	0.115	0.117
R^2 Adj.	0.122	0.145	0.119	0.110	0.112

+ p < 0.1, * p < 0.05

Table S10: Summary of individual-level moderation for the *belief* outcome in *Experiment 1*. Estimates come from linear regression models interacting the treatment indicator (`label`) with the relevant moderator variable. For social media sharing, the moderator is coded as 1 if respondents say they share news on social media, 0 otherwise. The remaining outcomes are standardized to have a mean of 0 and standard deviation of 1. All models include stimulus fixed effects.

	**Age**	**Digital Literacy**	**Party**	**Attentiveness**	**Sharing**
Label	−0.244*	−0.242*	−0.246*	−0.244*	−0.235*
	(0.040)	(0.040)	(0.040)	(0.040)	(0.063)
Moderator	−0.125*	−0.144*	0.055	−0.012	0.161*
	(0.037)	(0.039)	(0.039)	(0.038)	(0.074)
Label × Moderator	0.002	−0.057	−0.105*	−0.105*	−0.020
	(0.041)	(0.042)	(0.042)	(0.041)	(0.081)
Num. Obs.	4,187	4,188	4,185	4,188	4,184
R^2	0.113	0.136	0.101	0.110	0.103
R^2 Adj.	0.107	0.129	0.094	0.103	0.096

+ p < 0.1, * p < 0.05

Table S11: Summary of individual-level moderation for the *belief* outcome in *Experiment 2*. Estimates come from linear regression models interacting the treatment indicator (`label`) with the relevant moderator variable. For social media sharing, the moderator is coded as 1 if respondents say they share news on social media, 0 otherwise. The remaining outcomes are standardized to have a mean of 0 and standard deviation of 1. All models include stimulus fixed effects.

	Age	Digital Literacy	Party	Attentiveness	Sharing
Label	−0.151*	−0.143*	−0.159*	−0.152*	−0.139*
	(0.044)	(0.044)	(0.045)	(0.044)	(0.059)
Moderator	−0.237*	−0.177*	−0.092*	−0.183*	0.624*
	(0.036)	(0.042)	(0.043)	(0.042)	(0.075)
Label × Moderator	−0.034	−0.064	−0.087+	0.017	−0.016
	(0.040)	(0.046)	(0.048)	(0.046)	(0.082)
Num. Obs.	3,209	3,209	3,209	3,209	3,209
R^2	0.088	0.071	0.045	0.047	0.099
R^2 Adj.	0.083	0.066	0.041	0.042	0.095

+ $p < 0.1$, * $p < 0.05$

Table S12: Summary of individual-level moderation for the *sharing intentions* outcome in *Experiment 1*. Estimates come from linear regression models interacting the treatment indicator (`label`) with the relevant moderator variable. For social media sharing, the moderator is coded as 1 if respondents say they share news on social media, 0 otherwise. The remaining outcomes are standardized to have a mean of 0 and standard deviation of 1. All models include stimulus fixed effects.

	Age	Digital Literacy	Party	Attentiveness	Sharing
Label	−0.103*	−0.104*	−0.120*	−0.102*	−0.110*
	(0.042)	(0.042)	(0.043)	(0.043)	(0.053)
Moderator	−0.265*	−0.249*	−0.172*	−0.156*	0.622*
	(0.037)	(0.040)	(0.042)	(0.041)	(0.073)
Label × Moderator	−0.014	0.036	0.037	0.008	−0.012
	(0.040)	(0.044)	(0.046)	(0.044)	(0.078)
Num. Obs.	4,313	4,314	4,311	4,314	4,309
R^2	0.097	0.068	0.041	0.043	0.105
R^2 Adj.	0.090	0.062	0.034	0.036	0.099

+ $p < 0.1$, * $p < 0.05$

Table S13: Summary of individual-level moderation for the *sharing intentions* outcome in *Experiment 2*. Estimates come from linear regression models interacting the treatment indicator (`label`) with the relevant moderator variable. For social media sharing, the moderator is coded as 1 if respondents say they share news on social media, 0 otherwise. The remaining outcomes are standardized to have a mean of 0 and standard deviation of 1. All models include stimulus fixed effects.

Differences by Age

In our primary model specifications, summarized in Figure S22, we treat age as a continuous variable. To assess possible non-linearities in the interaction effects, we group respondents into equally sized age tertiles and estimate the effect of exposure to labeling within each tertile. Figure S23 plots the results for both studies. Overall, we find some evidence of heterogeneity by age, though there are notable discrepancies across studies. In Experiment 1, labeling tends to have the largest observed effects on self-reported sharing among middle-aged respondents—a pattern that persists for all four measures of engagement intentions. However, these patterns are less apparent for the belief outcome and less visible in Experiment 2.

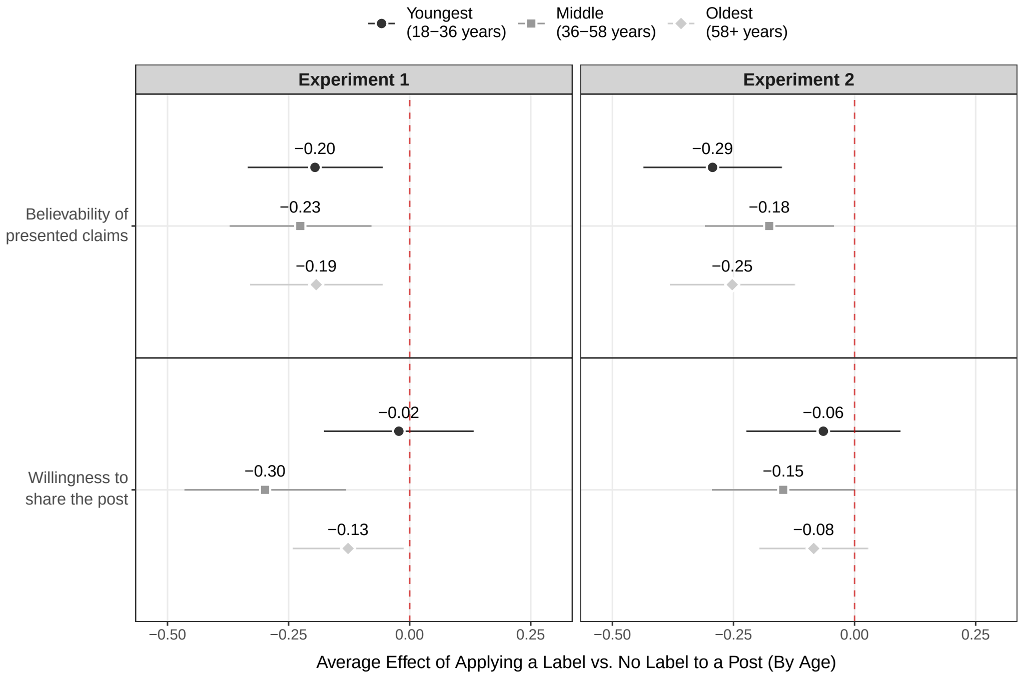

Figure S23: Average treatment effect of assignment to a labeled versus unlabeled post, disaggregated by *age* tertiles. Effects are expressed in units of standard deviation and are estimated using linear regression models with stimulus fixed effects that interact the treatment indicator with a categorical indicator of respondent age. 95% confidence intervals are based on robust standard errors.

Differences by Digital Literacy

Figure S24 disaggregates the overall labeling effects by digital literacy, as indexed by performance on a close-ended test assessing knowledge about computers, technology, and social media. Overall, we find in Experiment 1 that the effects of labeling on beliefs and behavior tend to be slightly smaller (and not distinguishable from zero) among respondents who exhibit the lowest levels of digital literacy. One possible implication of these results is that labeling may have a diminished impact among those who need it most—that is, people who might already be more susceptible to AI-generated misinformation absent intervention. Importantly, however, this attenuated effect of labeling for less digitally literate respondents is not visible for sharing intentions in Experiment 2.

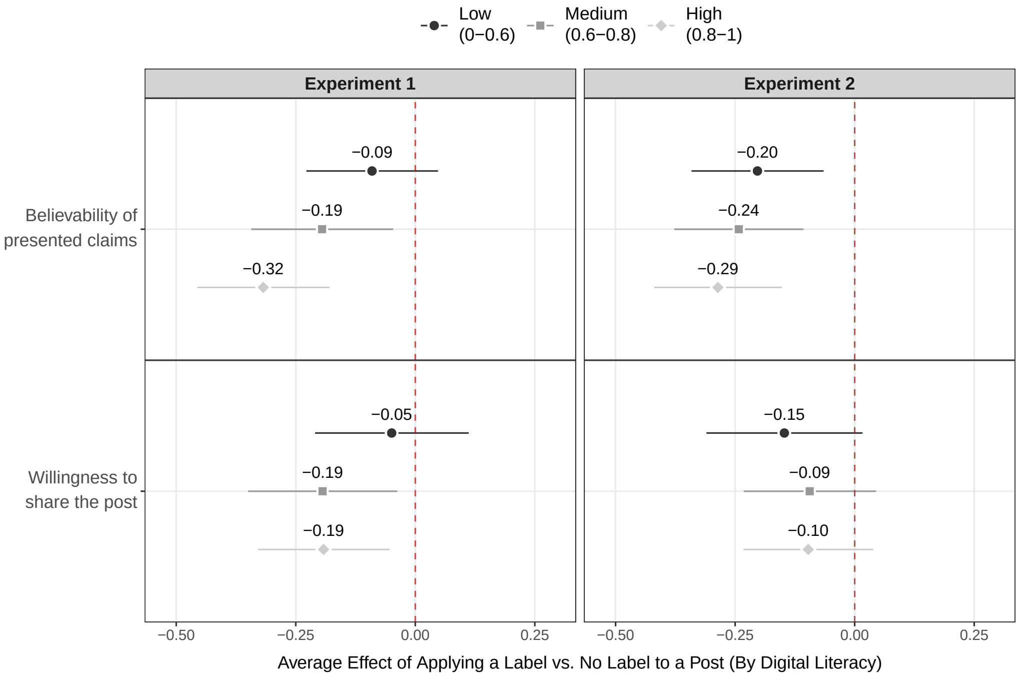

Figure S24: Average treatment effect of assignment to a labeled versus unlabeled post, disaggregated by *digital literacy* tertiles. Effects are expressed in units of standard deviation and are estimated using linear regression models with stimulus fixed effects that interact the treatment indicator with a categorical indicator of digital literacy. 95% confidence intervals are based on robust standard errors. Digital literacy scores range from 0-1, corresponding to the proportion of correct answers respondents gave on a five-item digital literacy test.

Differences by Attentiveness

Figure S25 disaggregates labeling effects by survey attentiveness. To measure attentiveness, we embedded two screeners throughout the pre-treatment demographics block; as such, attentiveness takes on a value of `0` if respondents answered neither item correctly (Exp. 1: n = 609; Exp. 2: n = 721), `0.5` if they answered only one item correctly (Exp. 1: n = 1,468; Exp. 2: n = 1,922), and `1` if they answered both correctly (Exp. 1: n = 1,146; Exp. 2: n = 1,713). The first, relatively simple screener asked respondents to select two pre-specified options from a set of five scale points ostensibly pertaining to political interest, and the second, more complex screener asked respondents to select two specific news outlets from a list of 18 options (for exact wording, see Section 1.4). In total, 78% of respondents in Experiment 1 and 80% of respondents in Experiment 2 correctly answered the first item, compared to 36% and 39%, respectively, for the second item.

As shown in the left-hand panel of this figure, we find in Experiment 1 that the effects of labeling are visible across all attentiveness strata. This result is notable, in that it suggests that labeling may still confer benefits among people who are not paying close attention to their online media environments. In Experiment 2, however, labeling has no discernible effect on beliefs among the least attentive respondents but is expected to have a larger impact as attentiveness increases. Future work should continue to explore the consequences of labeling in environments where such warning messages may be less eye-catching.

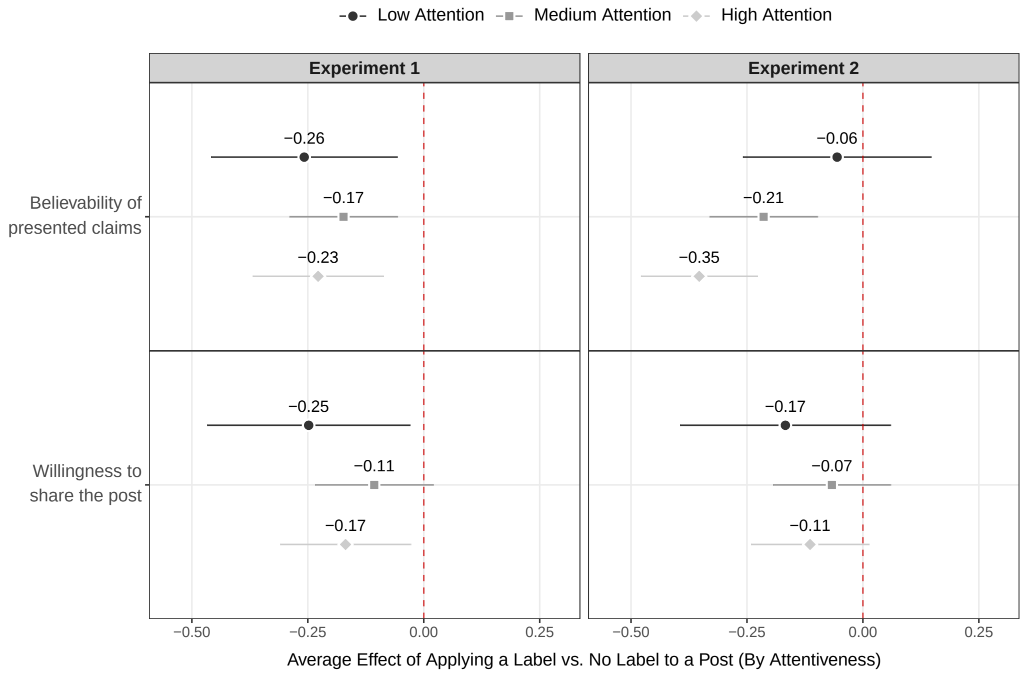

Figure S25: Average treatment effect of assignment to a labeled versus unlabeled post, disaggregated by *attentiveness.* Effects are expressed in units of standard deviation and are estimated using linear regression models with stimulus fixed effects that interact the treatment indicator with a categorical indicator of attentiveness. 95% confidence intervals are based on robust standard errors. Attentiveness takes on a value from 0-1, representing the proportion of correct responses to two attention checks.

Differences by Partisanship

Finally, Figure S26 and S27 plot partisan differences in labeling effects. For both of these plots, we treat "leaners" as partisans, though the results are substantively similar when focusing just on self-identified partisans. This exploratory analysis aims to build on prior work assessing differences in receptivity to misinformation interventions, including warning labels, along partisan lines. Prior work documents sharp partisan differences in baseline preferences for content moderation [13, 14]. However, it remains unclear whether warning labels exert varying effects on Republicans and Democrats. Whereas some prior research finds that fact-checking labels have little discernible impact among Republicans [9, 15, 16], other work suggests these labels can shift the beliefs and/or behavior of right-leaning consumers ([17, 18]; for a review, see [19]).

When it comes to AI labels, mirroring the results in Figure S22, we find in both studies that labeling has a larger effect on beliefs for Republican versus Democratic respondents, though the patterns for sharing intentions are inconsistent across studies. Notably, these results pool responses across the full stimulus set—which included both political *and* non-political posts. When we further break apart the results, we find that the stronger effects among Republicans are not just visible for political stimuli, suggesting that the observed partisan differences may be driven by factors other than the content of the presented posts. As a further caveat, because we do not directly manipulate partisan identity here, any partisan asymmetries may be attributable to demographic and/or social characteristics that vary across groups and should be interpreted with caution. Further disentangling these partisan differences—and replicating them across more diverse media—is therefore an important avenue for future research.

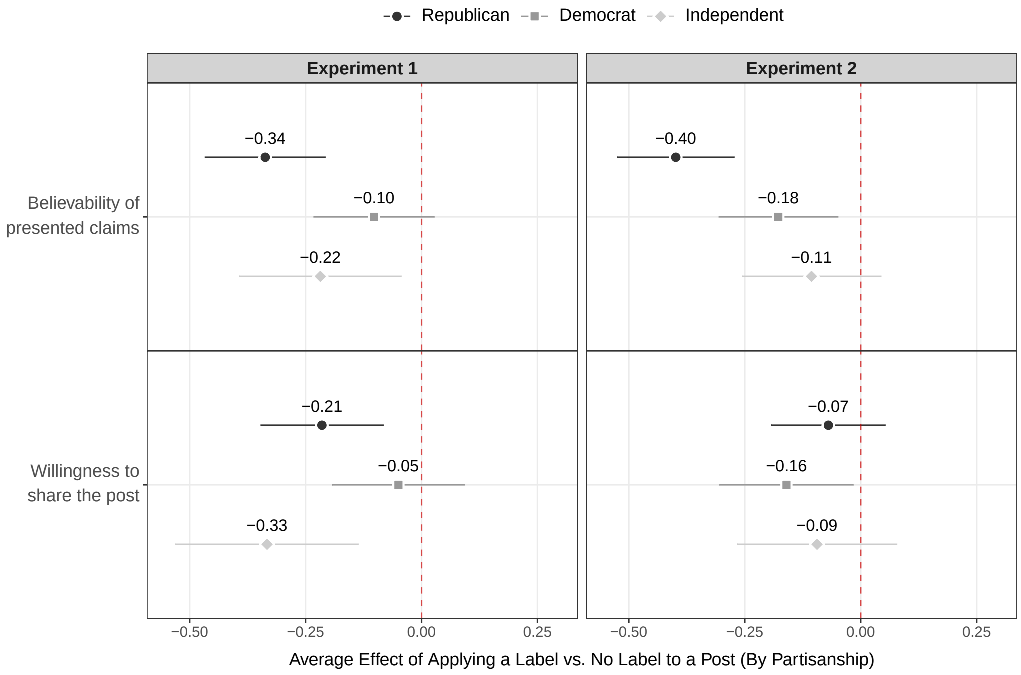

Figure S26: Average treatment effect of assignment to a labeled versus unlabeled post, disaggregated by *partisanship*. Effects are expressed in units of standard deviation and are estimated using linear regression models with stimulus fixed effects that interact the treatment indicator with a categorical indicator of respondent partisanship. 95% confidence intervals are based on robust standard errors.

(a) Experiment 1

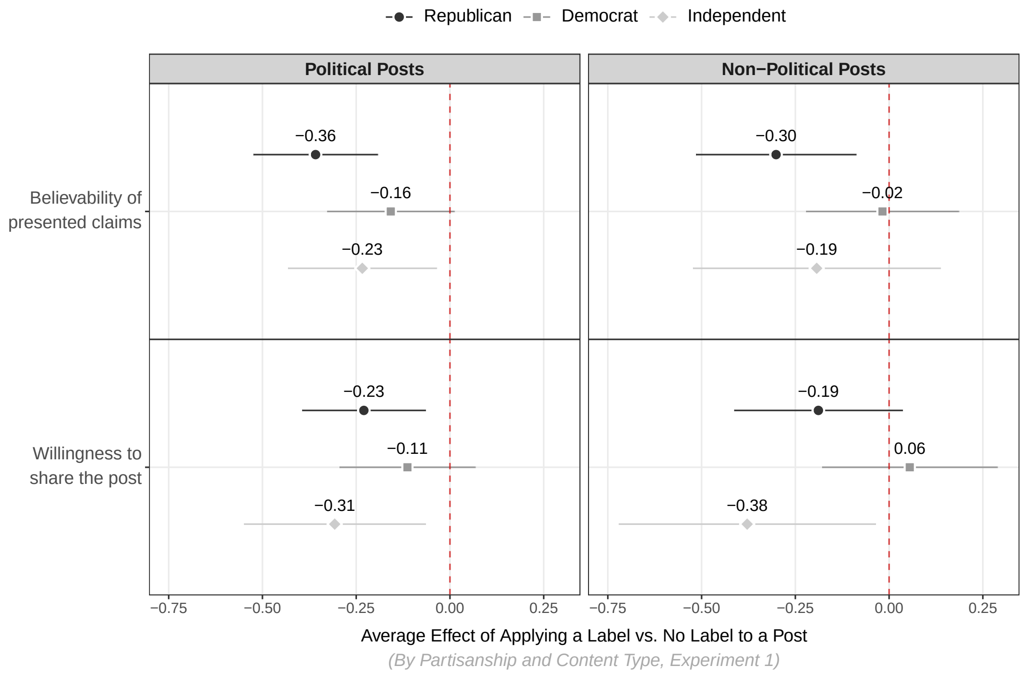

(b) Experiment 2

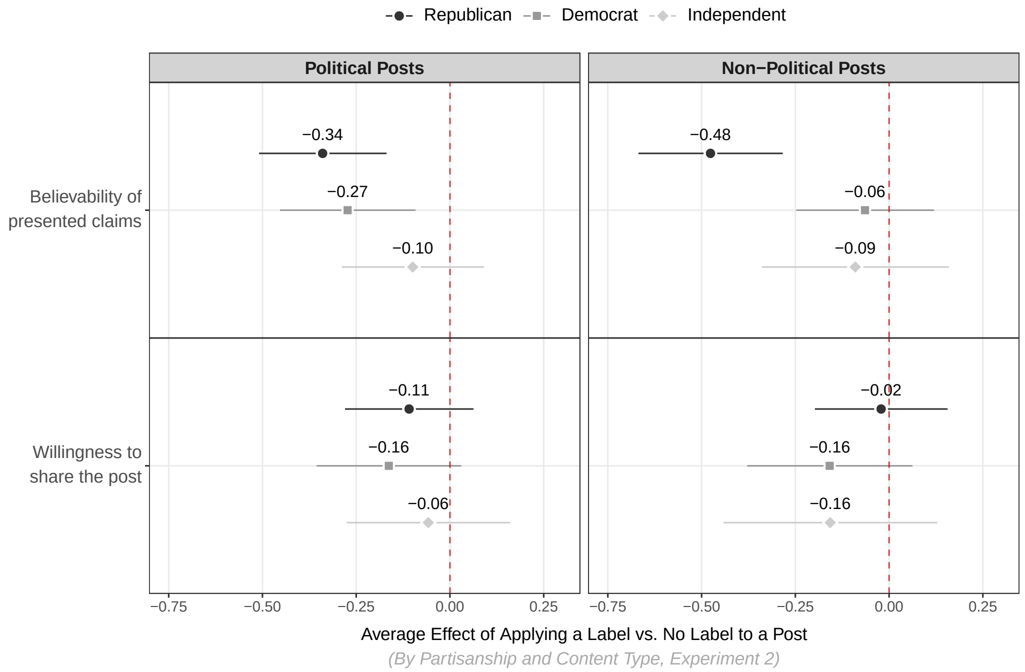

Figure S27: Average treatment effect of assignment to a labeled versus unlabeled post, disaggregated by *partisanship* and *content type* (political versus non-political posts). Effects are expressed in units of standard deviation and are estimated using linear regression models with stimulus fixed effects that interact the treatment indicator with a categorical indicator of respondent partisanship. 95% confidence intervals are based on robust standard errors.

4 Robustness Checks

4.1 Multi-Level Models

To assess the robustness of our results to alternative model specifications, we also estimated a series of Bayesian linear mixed-effect models using the brms package in R [20]. Each model allows both the intercept and treatment effects to vary across *posts*. The prior distributions for all parameters are vague and weakly informative. In all cases, we obtain satisfactory convergence ($\hat{R}$ values not exceeding 1.005, effective sample sizes >1000, and no divergent transitions during sampling), and trace plots indicate that the chains are sufficiently well mixed. For each experiment, we examine two quantities of interest. First, mirroring the results presented in SI Appendix, Section 2.1, we assess the effect of exposure to a label of *any* kind on viewers' beliefs and engagement intentions. To do so, we construct a dummy variable indicating whether respondents were shown a label of any kind (label), taking on a value of 1 if respondents were assigned to one of the labeling conditions, 0 if they were assigned to the unlabeled control group.

```
library(tidyverse)
library(brms)

# Overall effect pooling the labeling conditions
fit_pooled <- brm(data = df,
                  family = gaussian,
                  formula = outcome ~ 1 + label + (1 + label | image_id),
                  prior = c(prior(normal(3, 1.5), class = Intercept),
                            prior(normal(0, 2),   class = b),
                            prior(exponential(1), class = sd),
                            prior(exponential(1), class = sigma),
                            prior(lkj(2),         class = cor)),
                  iter = 5000, warmup = 1000, chains = 4, cores = 4,
                  seed = 531, control = list(adapt_delta = 0.99))
```

To replicate the results in the main text, we also fit analogous models estimating the effects of each label, relative to the unlabeled control group. The variable cond corresponds to a factor, with the control group set as the baseline category (analogous to inputting separate dummy variables for assignment to each of the distinct labels). The model structure otherwise remains unchanged from the previous specification.

```
# Effect of each label, relative to unlabeled control group
fit_disagg <- brm(data = df,
                  family = gaussian,
                  formula = outcome ~ 1 + cond + (1 + cond | image_id),
                  prior = c(prior(normal(3, 1.5), class = Intercept),
                            prior(normal(0, 2),   class = b),
                            prior(exponential(1), class = sd),
                            prior(exponential(1), class = sigma),
                            prior(lkj(2),         class = cor)),
```

```
                      iter = 5000, warmup = 1000, chains = 4, cores = 4,
                      seed = 531, control = list(adapt_delta = 0.99))
```

Finally, following the approach described in SI Appendix, Section 2.3, we estimate models for Experiment 2 that interact indicators of exposure to process and veracity cues. We focus on three key variables: (i) `ai_cent`, indicating whether respondents were informed that the content was AI-generated, (ii) `alt_cent`, indicating whether respondents were informed that the content was altered, and (iii) `mislead_cent`, indicating whether respondents were informed that the content could mislead people. The two process cues (`ai_cent` and `alt_cent`) are mutually exclusive, in that respondents could at most receive one of these signals. In all cases, we center-code the variables in order to facilitate interpretation of the resulting parameter values.

```
# Construct center-coded dummy variables
df <- df %>%
  # 1/0 dummy coding
  mutate(ai = case_when(str_detect(cond, "ai") ~ 1, TRUE ~ 0),
         alt = case_when(str_detect(cond, "altered") ~ 1, TRUE ~ 0),
         mislead = case_when(str_detect(cond, "mislead") ~ 1, TRUE ~ 0)) %>%
  # Center-code dummy variables
  mutate(ai_cent = scale(ai, center = T, scale = F),
         alt_cent = scale(alt, center = T, scale = F),
         mislead_cent = scale(mislead, center = T, scale = F))

# Interacting process and veracity cues (Study 2 only)
fit_int <- brm(data = df,
               family = gaussian,
               formula = outcome ~ 1 +
                 (ai_cent + alt_cent) * mislead_cent +
                 (1 + (ai_cent + alt_cent) * mislead_cent | image_id),
               prior = c(prior(normal(3, 1.5), class = Intercept),
                         prior(normal(0, 2),   class = b),
                         prior(exponential(1), class = sd),
                         prior(exponential(1), class = sigma),
                         prior(lkj(2),         class = cor)),
               iter = 5000, warmup = 1000, chains = 4, cores = 4,
               seed = 531, control = list(adapt_delta = 0.99))
```

In all cases, we allow both the intercept and treatment effect parameters to vary across posts, designated using the term `image_id`. However, it is important to recognize that each post was viewed a relatively small number of times; in Experiment 1 approximately 230 respondents, on average, saw each post (divided across five experimental conditions), while in Experiment 2 each stimulus was assigned to roughly 150 respondents (allocated to six experimental conditions). As such, estimates of post-level treatment effects based solely on the available data for each post (e.g., from separate OLS models for each stimulus) are likely to be imprecise and may overfit the data. Multi-level modeling, by contrast, induces some regularization of these individual estimates toward the population mean, which can improve the out-of-sample accuracy of these estimates on average [21].

Overall, we obtain nearly identical results when using hierarchical versus pooled regression models to estimate population-level treatment effects. As shown in Figure S28, exposure to a label of any kind decreases respondents' belief in the post's claims and likelihood of sharing, "liking"/favoriting, or seeking out additional information about the posts. Figure S29 likewise breaks apart the results in each experiment for each labeling condition; as this plot indicates, we again observe very similar patterns of treatment effects across model specifications, suggesting that our conclusions are robust to different analytical strategies. Finally, Table S14 reports the estimated parameters (posterior median and 95% HPDI) from analogous models interacting the two indicators of process and veracity cues.

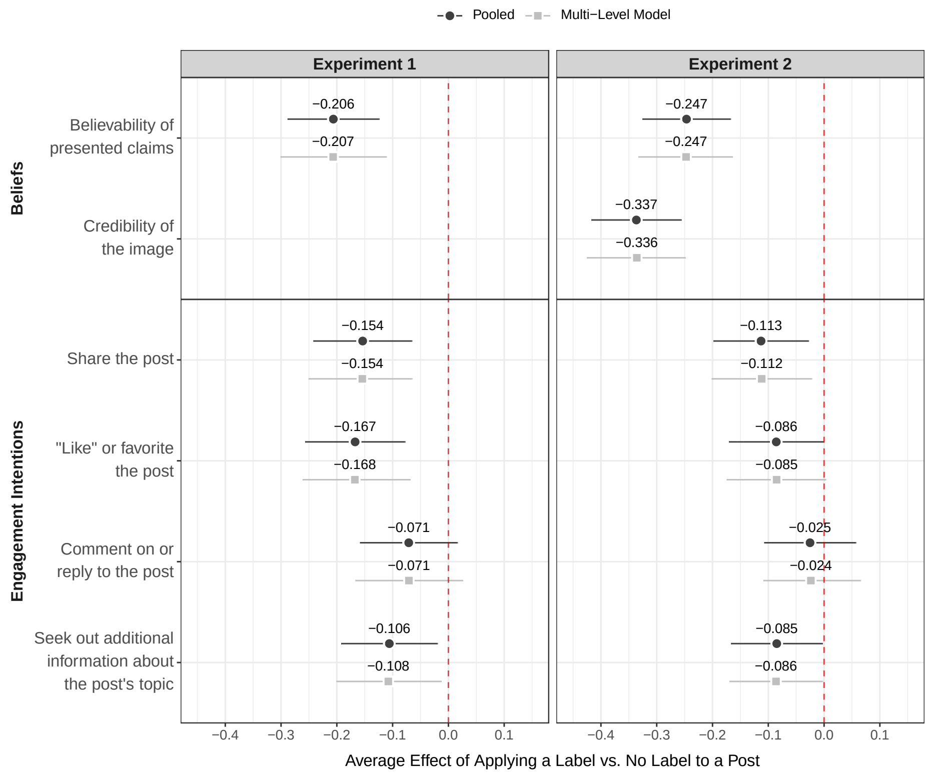

Figure S28: Average treatment effect of assignment to a labeled versus unlabeled post, estimated either using linear regression models with stimulus fixed effects (pooled) or Bayesian linear mixed-effect models with stimulus random effects (multi-level models). Effects are expressed in units of standard deviation. For the pooled models, 95% confidence intervals are based on robust standard errors. For the multi-level models, we report the posterior median and 95% highest posterior density interval. In all cases, negative values indicate that exposure to labeling decreased respondents' belief in or likelihood of engaging with their assigned post.

(a) Experiment 1

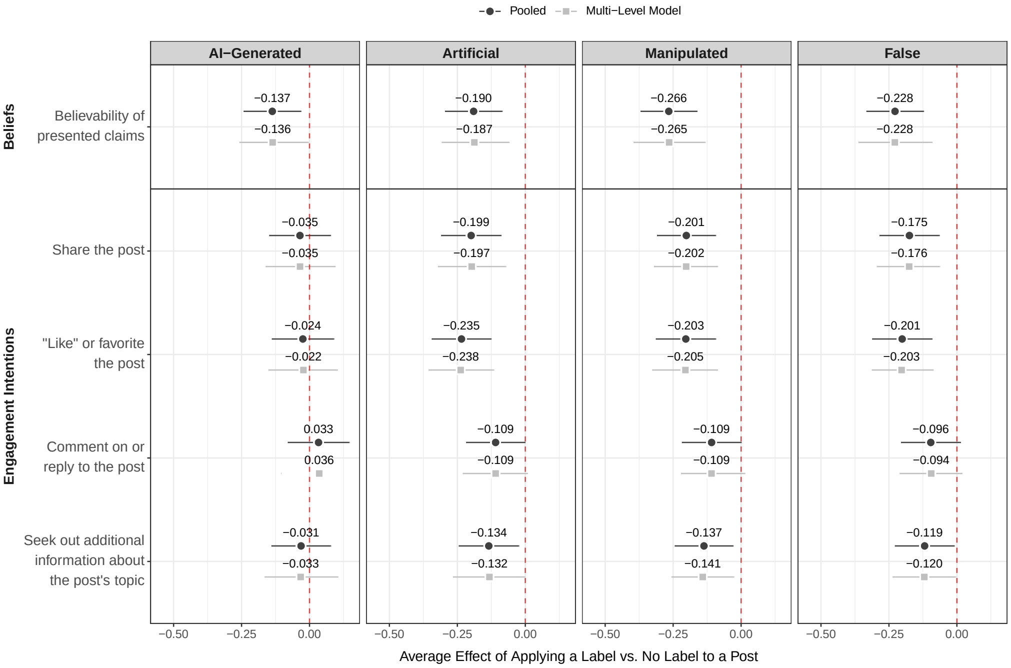

(b) Experiment 2

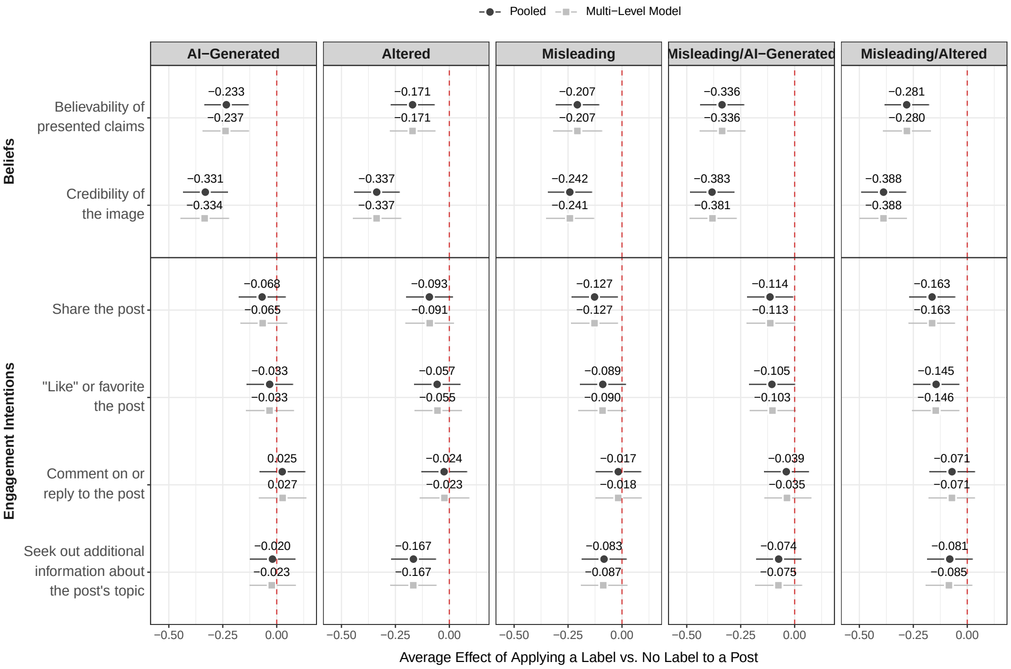

Figure S29: Average treatment effect of assignment to each label, relative to the control group, estimated either using linear regression models with stimulus fixed effects (pooled) or Bayesian linear mixed-effect models with stimulus random effects (multi-level models). Effects are expressed in units of standard deviation. For the pooled models, 95% confidence intervals are based on robust standard errors. For the multi-level models, we report the posterior median and 95% highest posterior density interval. In all cases, negative values indicate that exposure to labeling decreased respondents' belief in or likelihood of engaging with their assigned post.

	Beliefs	Credibility	Share	Like/Favorite	Comment/Reply	Seek Info
Intercept	2.196	2.156	1.422	1.421	1.449	1.608
	[2.075, 2.320]	[2.062, 2.258]	[1.368, 1.478]	[1.363, 1.485]	[1.399, 1.500]	[1.535, 1.678]
AI-Generated	-0.183	-0.235	-0.024	-0.023	0.004	-0.005
	[-0.258, -0.108]	[-0.317, -0.159]	[-0.109, 0.060]	[-0.101, 0.052]	[-0.072, 0.082]	[-0.081, 0.068]
Altered	-0.120	-0.240	-0.062	-0.055	-0.039	-0.081
	[-0.197, -0.047]	[-0.316, -0.164]	[-0.140, 0.013]	[-0.131, 0.022]	[-0.121, 0.036]	[-0.157, -0.006]
Misleading	-0.137	-0.112	-0.081	-0.084	-0.044	-0.017
	[-0.195, -0.075]	[-0.178, -0.050]	[-0.146, -0.017]	[-0.150, -0.018]	[-0.109, 0.020]	[-0.078, 0.045]
AI-Generated × Misleading	0.108	0.193	0.082	0.020	-0.047	0.032
	[-0.040, 0.266]	[0.043, 0.346]	[-0.073, 0.232]	[-0.136, 0.174]	[-0.202, 0.106]	[-0.136, 0.187]
Altered × Misleading	0.096	0.191	0.056	0.002	-0.032	0.168
	[-0.062, 0.252]	[0.038, 0.345]	[-0.104, 0.199]	[-0.151, 0.157]	[-0.187, 0.123]	[0.022, 0.315]
Num. Obs.	4,188	4,160	4,314	4,323	4,325	4,335

Table S14: Estimates from Bayesian multi-level models regressing each outcome variable on dummy variables for *process* and *veracity* cues. The posterior median and 95% highest posterior density intervals are reported; our conclusions are substantively unchanged if we instead use the posterior mean and 95% credible intervals. All dummy variables are center-coded to facilitate interpretation of the coefficients. The dependent variables are standardized by dividing responses by the standard deviation of the outcome scale.

4.2 Balance and Attrition Checks

Balance Checks

Figure S30 plots average values for (pre-treatment) covariates across treatment conditions and studies, and Tables S15 and S16 summarize the output of F-tests assessing balance across treatment conditions (for Experiments 1 and 2, respectively). For the most part, we observe rough covariance balance across conditions, as evidenced by F-tests that fail to reject the null hypothesis that mean covariate values are the same across conditions—with some exceptions. As a robustness check, we therefore re-estimate in Figures S31 and S32 our main treatment effects of interest from Figures 2 and 3, now controlling for these covariates, and find that the results remain substantively unchanged under this alternative model specification. For simplicity, we use the following shorthand for different variables:

- `female`: Whether a respondent self-identifies as a woman, versus a man or another gender identity (excluding those who prefer not to say)
- `age`: Self-reported age (in years)
- `race_bin`: Whether a respondent identifies as white (monoracial), versus another racial identity
- `educ_bin`: Whether a respondent has a college degree or higher, versus some college or less
- `diglit`: Digital literacy (score from 0-1)
- `screen_score`: Survey attentiveness (score from 0-1)
- `share_any`: Whether a respondent said they would share at least one type of news (about politics, sports, celebrities, science/technology, and/or business), versus would not share any of these types of content
- `party_rep`: Whether a respondent self-identifies as a Republican (including "leaners"), versus a Democrat or Independent
- `mobile_bin`: Whether a respondent took the survey using a mobile phone, versus a computer or tablet

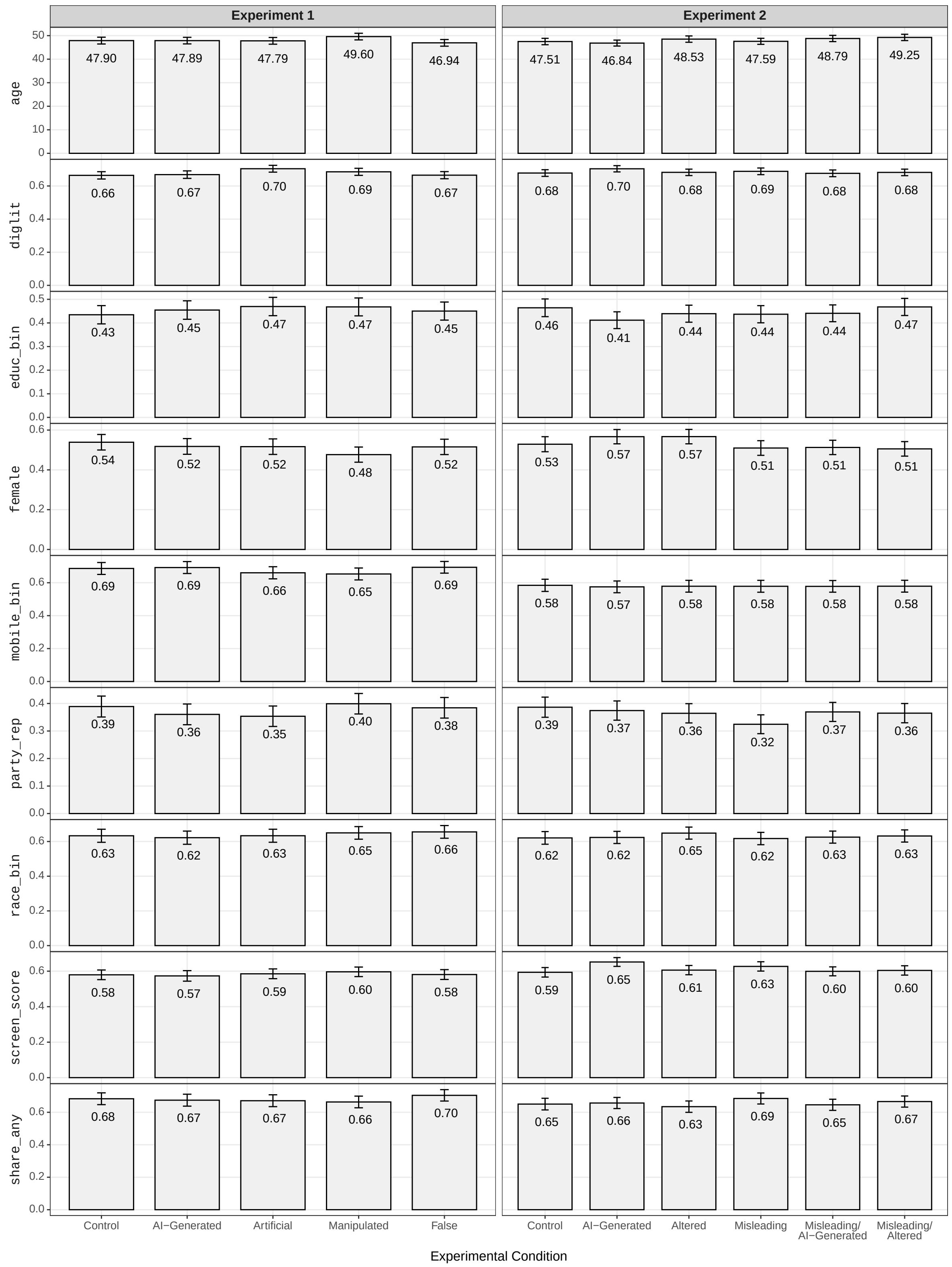

Figure S30: Mean covariate values across experimental conditions and studies. 95% confidence intervals on the mean are displayed. Descriptions of each variable are included above, and exact question wording is available in SI Appendix, Section 1.4. Note that device use (`mobile_bin`) was measured based on metadata stored in the web browser respondents used to take the survey.

Covariate	Res.Df	Df	F	Pr(>F)	Study
female	3,190	4	1.33	0.26	Experiment 1
age	3,218	4	1.82	0.12	Experiment 1
race_bin	3,216	4	0.52	0.72	Experiment 1
educ_bin	3,218	4	0.53	0.72	Experiment 1
diglit	**3,218**	**4**	**2.57**	**0.04**	**Experiment 1**
screen_score	3,218	4	0.37	0.83	Experiment 1
share_any	3,218	4	0.75	0.56	Experiment 1
party_rep	3,218	4	1.04	0.39	Experiment 1
mobile_bin	3,218	4	1.06	0.37	Experiment 1

Table S15: Heteroskedastic-robust F-tests assessing differences in mean covariate values across conditions (Experiment 1).

Covariate	Res.Df	Df	F	Pr(>F)	Study
female	**4,309**	**5**	**2.36**	**0.04**	**Experiment 2**
age	4,349	5	1.88	0.09	Experiment 2
race_bin	4,348	5	0.40	0.85	Experiment 2
educ_bin	4,350	5	1.24	0.29	Experiment 2
diglit	4,350	5	1.05	0.39	Experiment 2
screen_score	**4,350**	**5**	**2.87**	**0.01**	**Experiment 2**
share_any	4,345	5	1.00	0.42	Experiment 2
party_rep	4,347	5	1.40	0.22	Experiment 2
mobile_bin	4,350	5	0.03	1.00	Experiment 2

Table S16: Heteroskedastic-robust F-tests assessing differences in mean covariate values across conditions (Experiment 2).

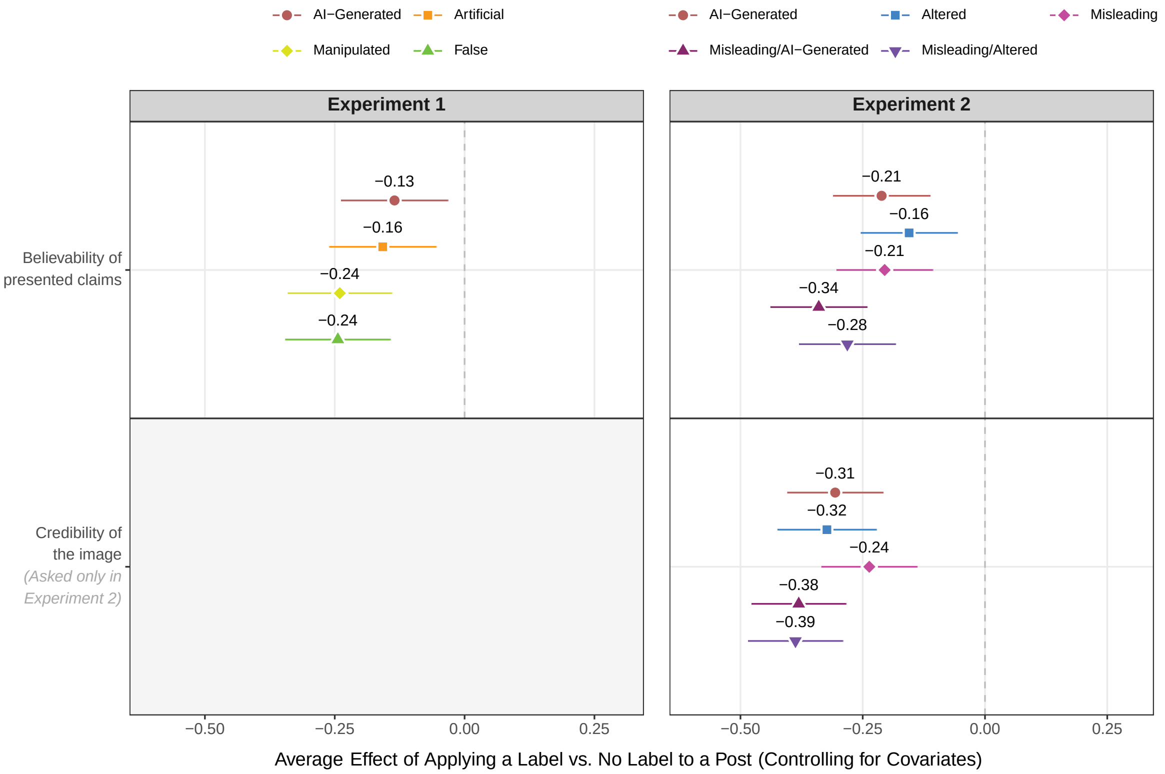

Figure S31: Average treatment effect of assignment to a labeled versus unlabeled post on *beliefs*, disaggregated by labeling condition and *controlling for pre-treatment covariates*. Effects are expressed in units of standard deviation and are estimated using linear regression models with stimulus fixed effects. 95% confidence intervals are based on robust standard errors. In all cases, negative values indicate that respondents assigned to a given label were less likely to believe the presented information, compared to respondents in the control group.

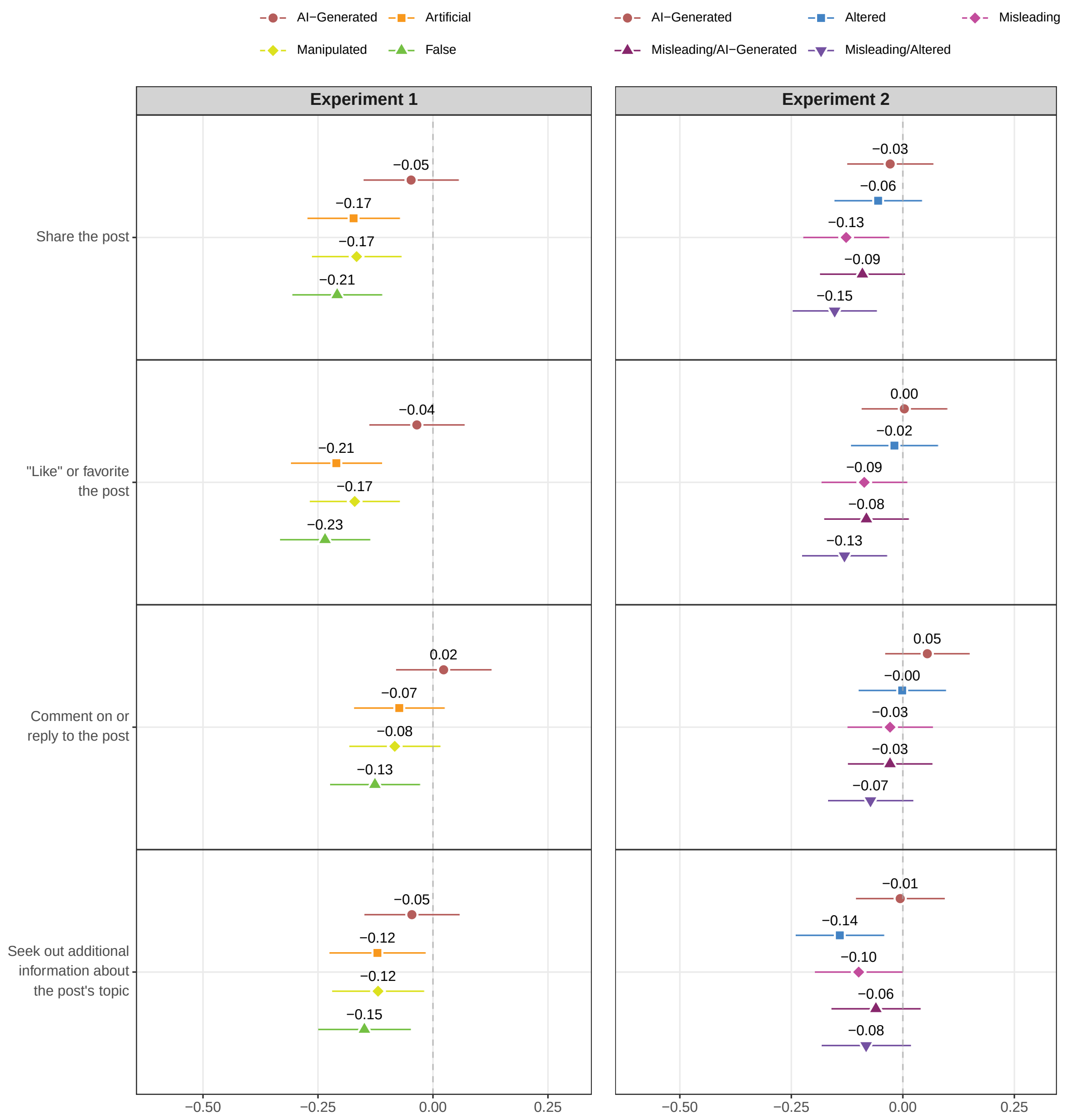

Figure S32: Average treatment effect of assignment to a labeled versus unlabeled post on *engagement intentions*, disaggregated by labeling condition and *controlling for pre-treatment covariates*. Effects are expressed in units of standard deviation and are estimated using linear regression models with stimulus fixed effects. 95% confidence intervals are based on robust standard errors. In all cases, negative values indicate that respondents assigned to a given label were less likely to say they would engage with the presented information, compared to respondents in the control group.

Attrition Checks

In this section, we examine whether respondents' likelihood of dropping out of our experiments varied based on their treatment status. Given the structure of our design, our surveys included a large number of post-treatment items, spread out across several question blocks. We therefore focus on three distinct points in the survey where attrition may have occurred:

- **Engagement intentions:** respondents were asked to complete the engagement intentions items immediately after viewing their assigned post (and, where applicable, the associated label).
  - To investigate attrition rates immediately post-exposure to the treatment, we therefore construct a dummy variable taking on a value of `1` if respondents failed to complete *any* of the four engagement intentions outcomes, `0` otherwise.
- **Beliefs:** respondents' beliefs about their assigned post were then collected after a brief distractor task lasting several minutes (on average).
  - To test for differential missingness on the belief outcome, we create a dummy variable taking on a value of `1` if a respondent did not report their beliefs, `0` otherwise.
- **Total drop-out:** finally, we examine whether overall rates of survey completion vary across experimental conditions (coded as `1` if a respondent did not finish the survey, `0` if they finished the survey).

Experiment 1

Table S17 summarizes drop-out rates for Experiment 1. Overall, we find minimal evidence of differential attrition across experimental conditions in this study, based on heteroskedastic-robust F-tests that fail to reject the null hypothesis that the attrition rate is equal across conditions. When it comes to engagement intentions, the amount of non-response is quite low in absolute terms and does not meaningfully vary across conditions ($df = 4$, F-statistic = 0.46, $p =$ 0.76). Rates of missingness unsurprisingly increase at later points in the survey, though we again do not detect differential attrition when examining the belief outcome ($df = 4$, F-statistic = 0.20, $p = 0.94$) or when looking at respondents who did versus did not reach the end of the survey (df = 4, F-statistic = 0.37, $p = 0.83$).

		Engagement Intentions		*Beliefs*		*Total Drop-out*	
**Condition**	**# Total**	**# Drop**	**Prop. Drop**	**# Drop**	**Prop. Drop**	**# Drop**	**Prop. Drop**
`control`	642	7	0.011	36	0.056	45	0.070
`ai`	633	6	0.009	31	0.049	47	0.074
`artificial`	646	7	0.011	37	0.057	52	0.080
`false`	660	7	0.011	38	0.058	56	0.085
`manipulated`	681	12	0.018	40	0.059	57	0.084

Table S17: Summary of drop-out rates in *Experiment 1.* Respondents were assigned to labeling conditions using simple randomization (with equal probabilities); deviations in total counts reflect sampling variability. We calculate drop-out at three points in the survey: (i) prior to completing at least one engagement intention item, (ii) prior to completing the belief outcome, and (iii) prior to submission.

Experiment 2

Table S18 summarizes these same patterns for Experiment 2. Notably, we observe variable rates of drop-out across conditions immediately post-treatment for the engagement intentions items, with approximately twice as many instances of drop-out in the "Misleading/Altered" condition, relative to the other five groups. Given the relatively small number of missing cases, these differences between conditions are not detectable using heteroskedastic-robust F-tests ($df = 5$, F-statistic = 1.30, $p = 0.26$). Differential drop-out rates by condition are also not statistically distinguishable at subsequent points in the survey, including for the belief outcome ($df = 5$, F-statistic = 1.52, $p = 0.18$) or when examining final completion rates ($df = 5$, F-statistic = 1.38, $p = 0.23$).

Notably, higher rates of attrition are solely visible for the "Misleading/Altered" condition, whereas our primary quantities of interest (particularly for engagement intentions) center on the AI-specific process labels. In addition, it is important to note that the amount of attrition immediately post-exposure is relatively small in absolute terms (1.6% of total observations), with only 13 cases separating the conditions with the most and least drop-out. As a result, it is unlikely that the behavioral results in Experiment 2 are driven by differential attrition across labeling groups.

		Engagement Intentions		*Beliefs*		*Total Drop-out*	
**Condition**	**# Total**	**# Drop**	**Prop. Drop**	**# Drop**	**Prop. Drop**	**# Drop**	**Prop. Drop**
`control`	694	11	0.016	38	0.055	53	0.076
`ai`	751	10	0.013	40	0.053	68	0.091
`altered`	739	9	0.012	44	0.060	79	0.107
`misleading`	729	9	0.012	34	0.047	61	0.084
`misleading_ai`	762	11	0.014	30	0.039	73	0.096
`misleading_altered`	750	22	0.029	51	0.068	81	0.108

Table S18: Summary of drop-out rates in *Experiment 2*. Respondents were assigned to labeling conditions using simple randomization (with equal probabilities); deviations in total counts reflect sampling variability. We calculate drop-out at three points in the survey: (i) prior to completing at least one engagement intention item, (ii) prior to completing the belief outcome, and (iii) prior to submission.

Nevertheless, to assess the robustness of our results to differential attrition, we impute outcome values for cases of non-response using ratings from the unlabeled control group. This marks a slight departure from our pre-analysis plan, where we indicated we would construct Lee bounds; we opt for this alternative approach in light of the relatively low volume of missing data and the presence of differential attrition for only one, auxiliary treatment arm. Specifically, we model responses in the control group as a function of age, digital literacy, education, attentiveness, device (i.e., mobile versus desktop), and assigned post (e.g., `trump_arrest` or `hybrid_turtle`). We exclude several of the individual-level moderators described in Section 3.3 (partisanship and social media use) due to missingness in these variables. Theoretically, this approach offers a conservative estimate of the intent-to-treat effect, assuming no backfire among treated respondents who failed to complete a given item. Figure S33 summarizes the results of this robustness check; given the small amount of missing data, we find that this imputation strategy has minimal impact on our treatment effect estimates.

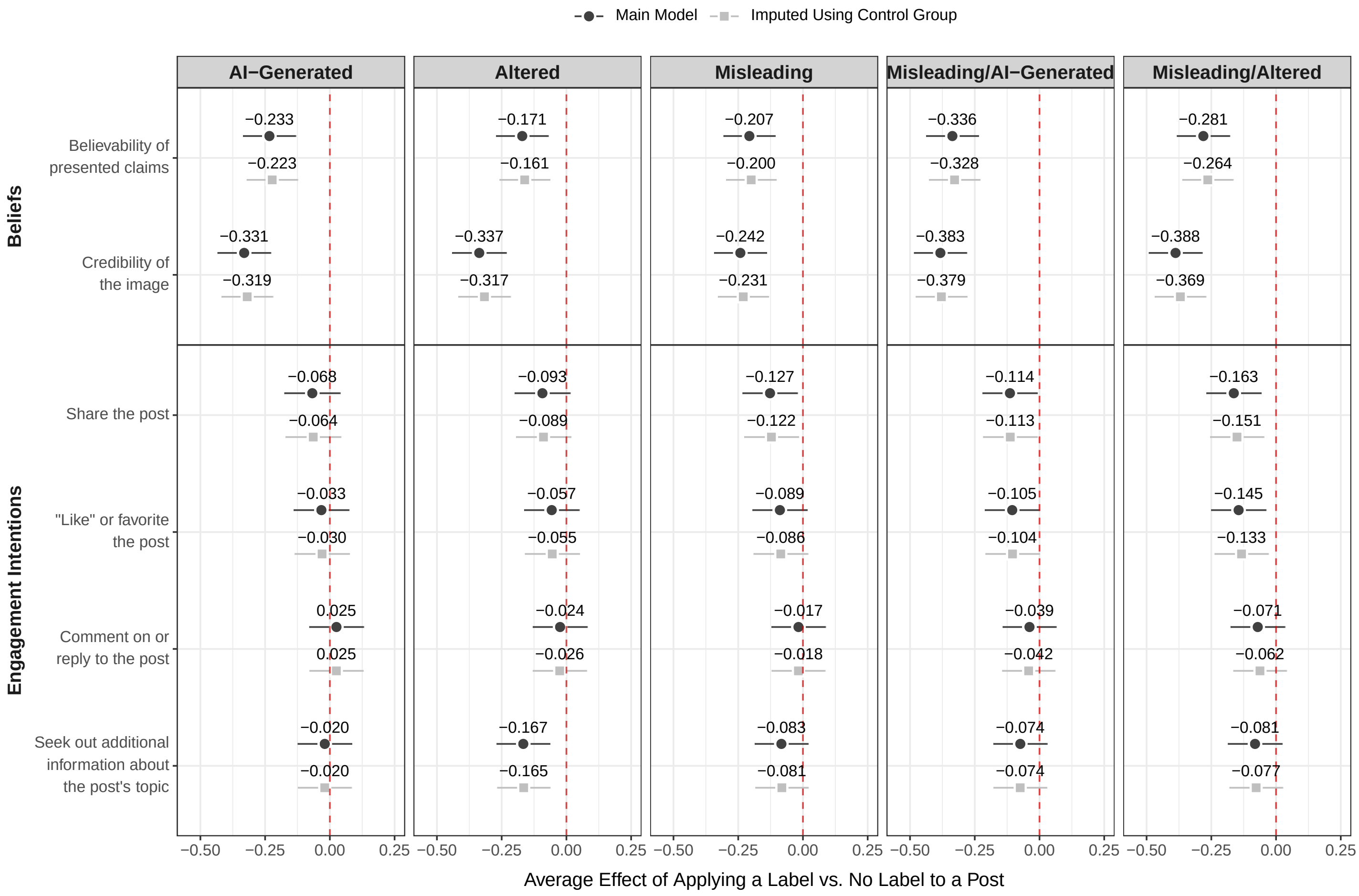

Figure S33: Estimated treatment effects pre- and post-imputation. To impute values for missing outcomes, we generate predictions from linear models regressing responses within the control group on a series of pre-treatment covariates, under an assumption of no backfire among missing cases. The outcome variables in all cases are treated as continuous; the results are substantively identical if we constrain the Likert-scale measures (all except image credibility) to take on an integer value from 1-5.

4.3 Correction for Multiple Comparisons

Given our goal of comparing different labeling strategies—both to each other and to an unlabeled benchmark—each of our experiments included a large number of treatment arms (five in Experiment 1, six in Experiment 2) and outcome variables. Following our pre-analysis plan, we therefore report both unadjusted p-values from our main model specifications and p-values that correct for multiple comparisons. Specifically, we control the false discovery rate (FDR) using the Benjamini-Hochberg procedure [22] and the family-wise error rate (FWER) using the Holm-Bonferroni method [23]. The FWER corresponds to the probability of erroneously rejecting *any* null hypotheses across all tests—that is, the probability of committing at least one Type I error across any of our comparisons—whereas the FDR corresponds to the expected proportion of Type I errors among all discoveries (i.e., statistically significant tests). For the sake of transparency, we report adjusted p-values for both of these methods, though FWER-adjusted p-values may be overly conservative for complex research designs with many treatment arms and dependent variables [24].

Throughout, we focus on two sets of outcome variables: *beliefs* and *engagement intentions*. The belief outcomes include respondents' stated belief in the post's core claims and, in Experiment 2, the perceived credibility of the image. The engagement intentions items include self-reported sharing, "liking"/favoriting, commenting/replying, and information-seeking. We do not examine in this section any variables that were solely used for exploratory analyses, including the outcomes related to label evaluations. For each experiment, we adjust for three sets of comparisons together: (i) our pre-registered aggregate comparison of respondents assigned to one of the treatment arms (i.e., a labeled post) versus the unlabeled control group (see Section 2.1), (ii) a comparison of each label's individual performance versus the unlabeled control group, and (iii) a comparison of each label to the standard "AI-Generated" label.

In general, when it comes to key outcome variables associated with the persuasiveness of AI-generated misinformation, the results presented in the main text appear largely robust to adjustments controlling the FDR. However, especially given the large number of comparisons, we cannot always detect relatively small differences in the behavioral impact of various labeling strategies, particularly in Experiment 2.

Aggregate Effects of Labeling

Figure S34 plots the p-values for our aggregate analyses assessing the impact of exposure to labeling of any kind. As shown in the top row of this figure, we continue to find strong evidence in both studies that labeling reduces the believability of presented content, even after accounting for multiple testing. In addition, after controlling the FDR we still find in Experiment 1 that labeling significantly decreases respondents' stated willingness to share, "like"/favorite, and seek out additional information about posts containing AI-generated media, though the last result does not persist after applying the more conservative Holm-Bonferroni correction. In Experiment 2, the effect of labeling on sharing intentions remains significant after controlling the FDR, though we can no longer detect an effect of labeling on other types of behavior after making either type of adjustment.

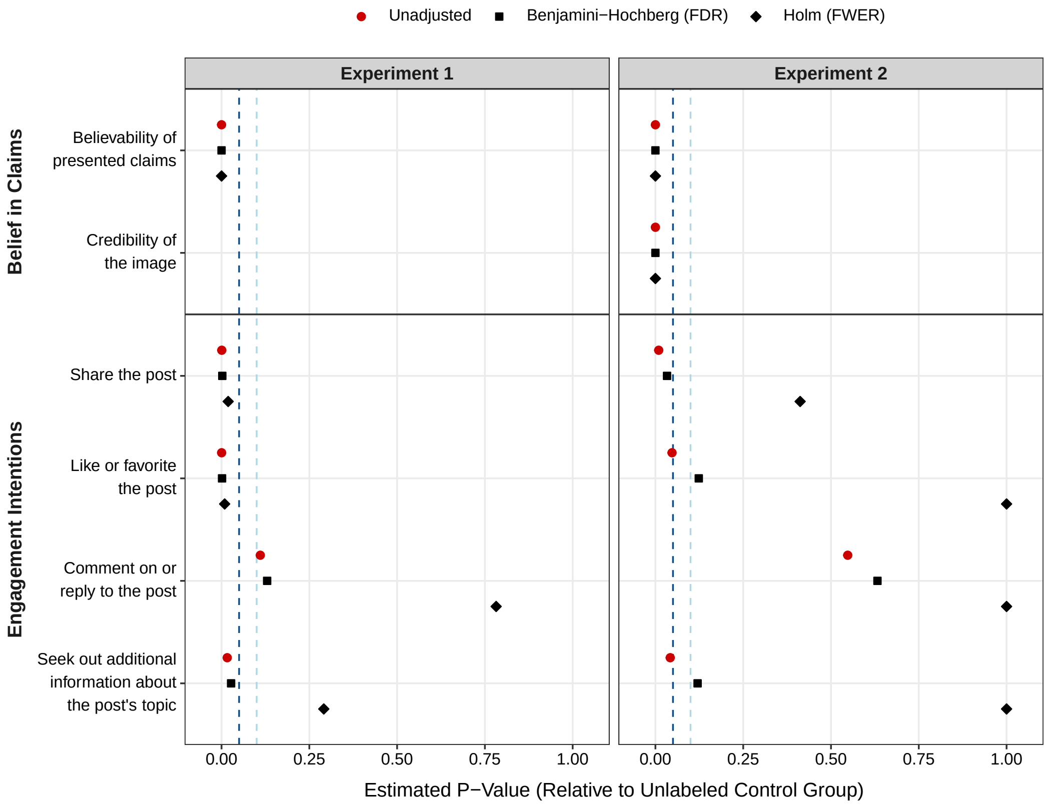

Figure S34: Adjusted and unadjusted p-values for the effect of assignment to a labeled versus unlabeled version of a post. The dark blue hashed line denotes $p = 0.05$, and the light blue hashed line denotes $p = 0.10$. We present results from two correction methods—one controlling the FDR (using the Benjamini-Hochberg step-up procedure) and one controlling the FWER (using the Holm-Bonferroni method). The perceived credibility of the image was only measured in Experiment 2.

Differences Across Labels

Figure S35 reports unadjusted and adjusted p-values when comparing each of the different labeling conditions to the unlabeled control group. When it comes to beliefs, our results are largely robust to adjustments for multiple comparisons: across experiments, respondents are significantly less likely to express belief in the post's claims or ascribe credibility to the image after viewing a labeled versus unlabeled version of the post. The sole exception to this is the "AI-Generated" label in Experiment 1, whose effect on beliefs is no longer distinguishable from zero after applying the Holm-Bonferroni correction. When it comes to engagement intentions, controlling the FDR renders a number of the results statistically insignificant, particularly for the sharing and "liking"/favoriting outcomes in Experiment 2.

Figure S36 instead compares respondents assigned to the "AI-Generated" label versus another label. As shown in the top panel of this figure, our substantive conclusions in Experiment 1 about differences between AI and non-AI labels are largely unchanged after controlling the FDR; on the whole, the AI label continues to have significantly smaller effects on on-platform behavior (including self-reported sharing, "liking"/favoriting, and commenting/replying), compared to other labels, even after applying the Benjamini-Hochberg correction.

(a) Experiment 1

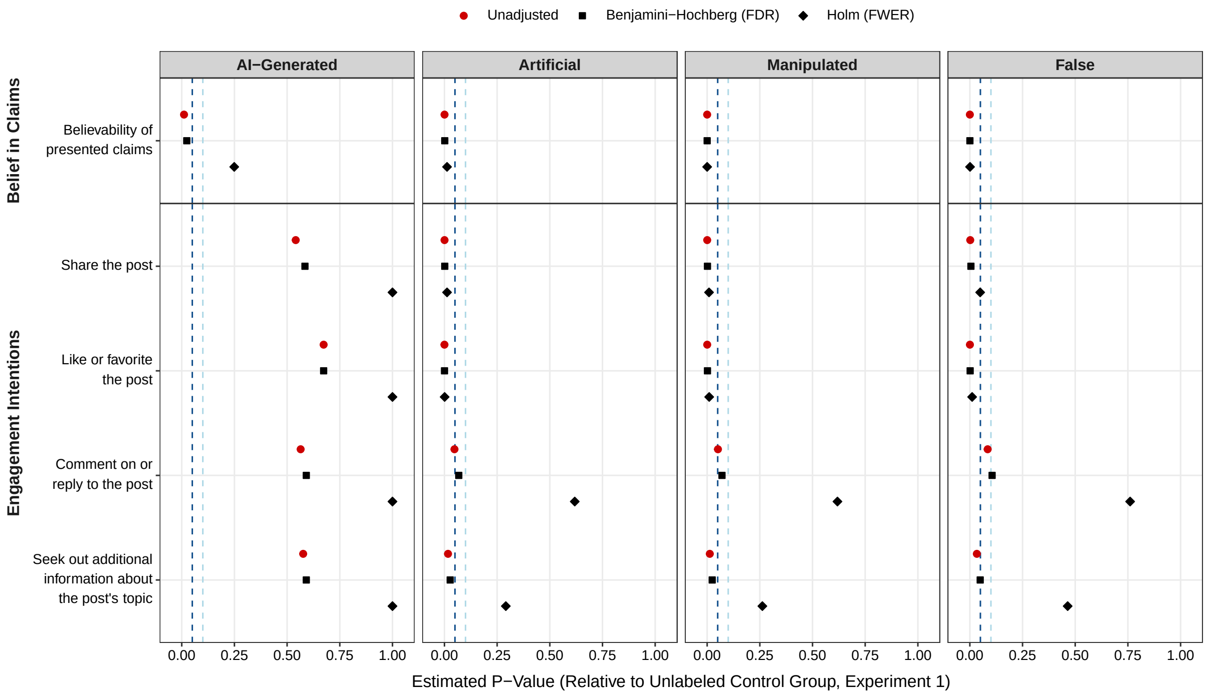

(b) Experiment 2

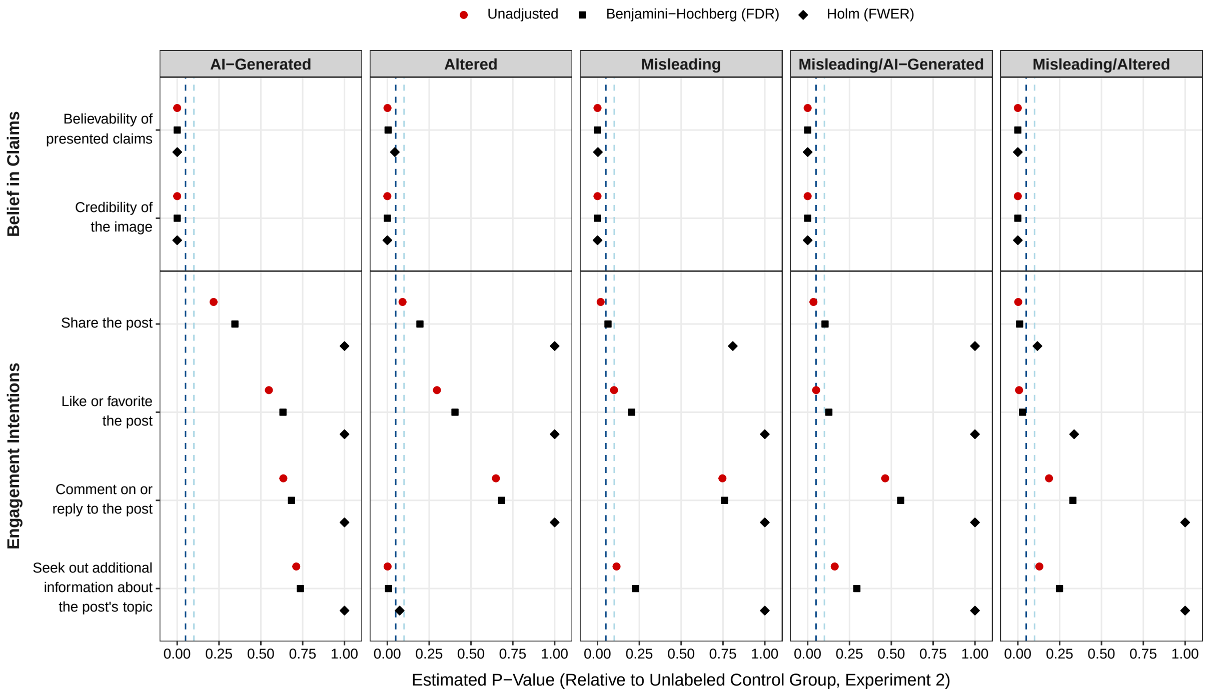

Figure S35: Adjusted and unadjusted p-values for the effect of assignment to a labeled versus unlabeled version of a post, disaggregated by labeling condition. The dark blue hashed line denotes $p = 0.05$, and the light blue hashed line denotes $p = 0.10$. We present results from two correction methods—one controlling the FDR (using the Benjamini-Hochberg step-up procedure) and one controlling the FWER (using the Holm-Bonferroni method). The perceived credibility of the image was only measured in Experiment 2.

(a) Experiment 1

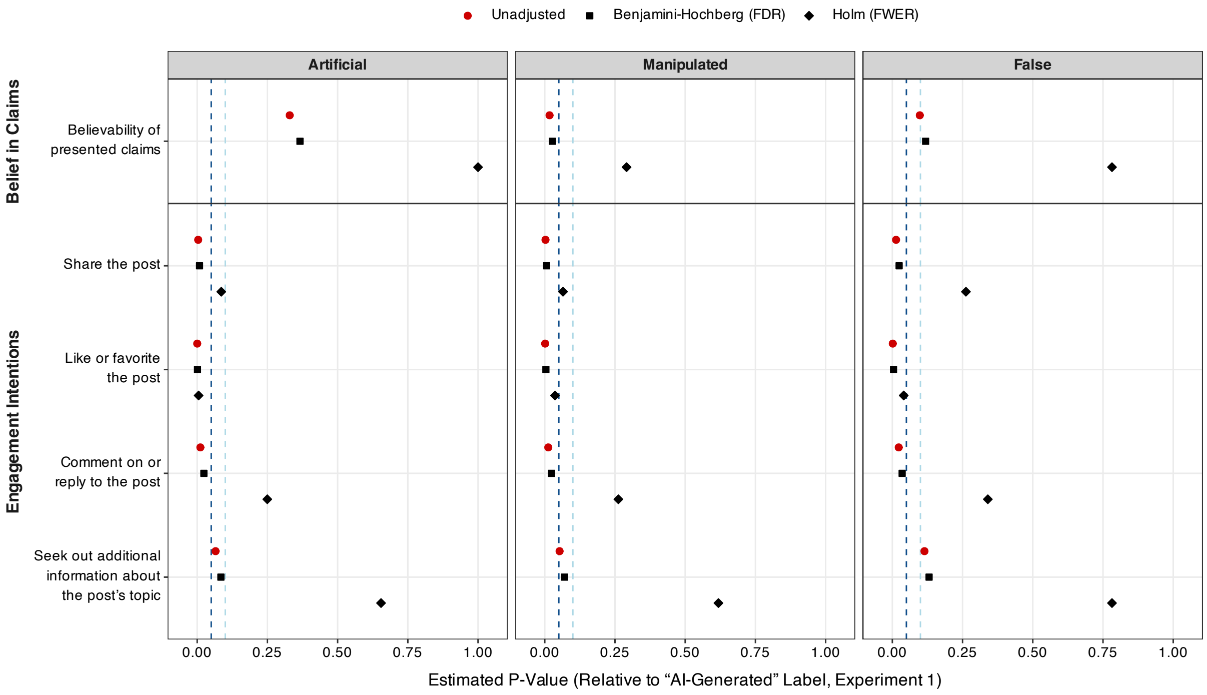

(b) Experiment 2

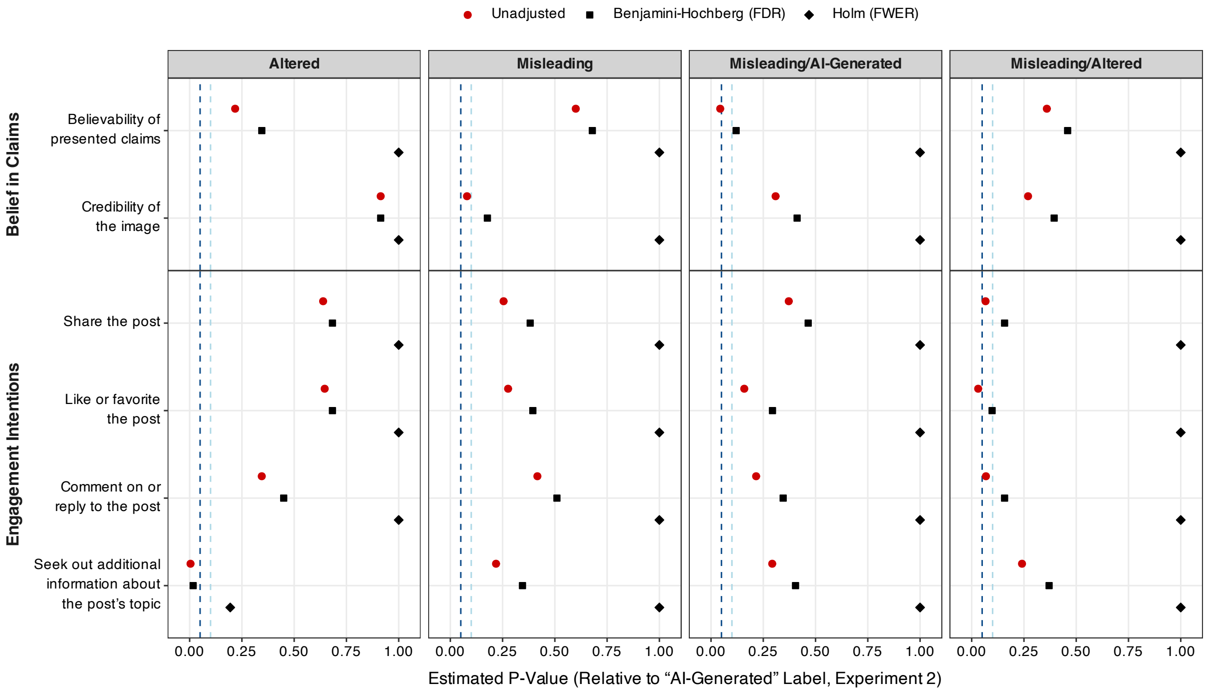

Figure S36: Adjusted and unadjusted p-values for the effect of assignment to the "AI-Generated" label in each study, relative to another label. The dark blue hashed line denotes $p = 0.05$, and the light blue hashed line denotes $p = 0.10$. We present results from two correction methods—one controlling the FDR (using the Benjamini-Hochberg step-up procedure) and one controlling the FWER (using the Holm-Bonferroni method). The perceived credibility of the image was only measured in Experiment 2.

Supplemental References

1. Epstein, Z., Fang, M. C., Arechar, A. A. & Rand, D. *What label should be applied to content produced by generative AI?* 2023. https://psyarxiv.com/v4mfz/.
2. Clayton, K., Blair, S., Busam, J. A., Forstner, S., Glance, J., Green, G., Kawata, A., Kovvuri, A., Martin, J., Morgan, E., Sandhu, M., Sang, R., Scholz-Bright, R., Welch, A. T., Wolff, A. G., Zhou, A. & Nyhan, B. Real Solutions for Fake News? Measuring the Effectiveness of General Warnings and Fact-Check Tags in Reducing Belief in False Stories on Social Media. *Political Behavior* **42,** 1073–1095 (2020).
3. Guo, C., Zheng, N. & Guo, C. Seeing is Not Believing: A Nuanced View of Misinformation Warning Efficacy on Video-Sharing Social Media Platforms. *Proceedings of the ACM on Human-Computer Interaction* **7,** 294:1–294:35 (2023).
4. Epstein, Z., Foppiani, N., Hilgard, S., Sharma, S., Glassman, E. & Rand, D. Do Explanations Increase the Effectiveness of AI-Crowd Generated Fake News Warnings? *Proceedings of the International AAAI Conference on Web and Social Media* **16,** 183–193 (2022).
5. Renault, T., Amariles, D. R. & Troussel, A. *Collaboratively adding context to social media posts reduces the sharing of false news* (2024). http://arxiv.org/abs/2404.02803.
6. Sharevski, F., Devine, A., Jachim, P. & Pieroni, E. *Meaningful Context, a Red Flag, or Both? Preferences for Enhanced Misinformation Warnings Among US Twitter Users* in *Proceedings of the 2022 European Symposium on Usable Security* (ACM, Karlsruhe Germany, 2022), 189–201. https://dl.acm.org/doi/10.1145/3549015.3555671.
7. Garrett, R. K. & Poulsen, S. Flagging Facebook Falsehoods: Self-Identified Humor Warnings Outperform Fact Checker and Peer Warnings. *Journal of Computer-Mediated Communication* **24,** 240–258 (2019).
8. Jia, C., Boltz, A., Zhang, A., Chen, A. & Lee, M. K. Understanding Effects of Algorithmic vs. Community Label on Perceived Accuracy of Hyper-partisan Misinformation. *Proceedings of the ACM on Human-Computer Interaction* **6,** 371:1–371:27 (2022).
9. Yaqub, W., Kakhidze, O., Brockman, M. L., Memon, N. & Patil, S. *Effects of Credibility Indicators on Social Media News Sharing Intent* in *Proceedings of the 2020 CHI Conference on Human Factors in Computing Systems* (Association for Computing Machinery, New York, NY, USA, 2020), 1–14. https://dl.acm.org/doi/10.1145/3313831.3376213.
10. Arel-Bundock, V., Greifer, N. & Heiss, A. How to Interpret Statistical Models Using marginaleffects in R and Python. *Journal of Statistical Software.*
11. Hainmueller, J., Mummolo, J. & Xu, Y. How Much Should We Trust Estimates from Multiplicative Interaction Models? Simple Tools to Improve Empirical Practice. *Political Analysis* **27,** 163–192 (2019).
12. Berinsky, A. J., Margolis, M. F. & Sances, M. W. Separating the Shirkers from the Workers? Making Sure Respondents Pay Attention on Self-Administered Surveys. *American Journal of Political Science* **58,** 739–753 (2014).

13. Appel, R. E., Pan, J. & Roberts, M. E. Partisan conflict over content moderation is more than disagreement about facts. *Science Advances* **9,** eadg6799 (2023).

14. Kozyreva, A., Herzog, S. M., Lewandowsky, S., Hertwig, R., Lorenz-Spreen, P., Leiser, M. & Reifler, J. Resolving content moderation dilemmas between free speech and harmful misinformation. *Proceedings of the National Academy of Sciences* **120,** e2210666120 (2023).

15. Jennings, J. & Stroud, N. J. Asymmetric adjustment: Partisanship and correcting misinformation on Facebook. *New Media & Society* **25,** 1501–1521 (2023).

16. Lees, J., McCarter, A. & Sarno, D. M. Twitter's Disputed Tags May Be Ineffective at Reducing Belief in Fake News and Only Reduce Intentions to Share Fake News Among Democrats and Independents. *Journal of Online Trust and Safety* **1** (2022).

17. Martel, C. & Rand, D. G. Fact-checker warning labels are effective even for those who distrust fact-checkers. *Nature Human Behaviour,* 1–11 (2024).

18. Porter, E. & Wood, T. J. Political Misinformation and Factual Corrections on the Facebook News Feed: Experimental Evidence. *The Journal of Politics* **84,** 1812–1817 (2022).

19. Martel, C. & Rand, D. G. Misinformation warning labels are widely effective: A review of warning effects and their moderating features. *Current Opinion in Psychology* **54,** 101710 (2023).

20. Bürkner, P.-C. brms: An R Package for Bayesian Multilevel Models Using Stan. *Journal of Statistical Software* **80,** 1–28 (2017).

21. McElreath, R. *Statistical Rethinking: A Bayesian Course with Examples in R and STAN* 2nd edition (Chapman & Hall/CRC, Boca Raton, FL, 2020).

22. Benjamini, Y. & Hochberg, Y. Controlling the False Discovery Rate: A Practical and Powerful Approach to Multiple Testing. *Journal of the Royal Statistical Society: Series B (Methodological)* **57,** 289–300 (1995).

23. Holm, S. A Simple Sequentially Rejective Multiple Test Procedure. *Scandinavian Journal of Statistics* **6,** 65–70 (1979).

24. Michler, J. D. & Josephson, A. *Recent Developments in Inference: Practicalities for Applied Economics* 2021. http://arxiv.org/abs/2107.09736 (2024).
